# Access to a Labile Monomeric Magnesium Radical by Ball‐Milling

**DOI:** 10.1002/anie.202200511

**Published:** 2022-02-18

**Authors:** Dawid Jędrzkiewicz, Jonathan Mai, Jens Langer, Zachary Mathe, Neha Patel, Serena DeBeer, Sjoerd Harder

**Affiliations:** ^1^ Inorganic and Organometallic Chemistry Universität Erlangen-Nürnberg Egerlandstrasse 1 91058 Erlangen Germany; ^2^ Max Planck Institute for Chemical Energy Conversion Stiftstr. 34–36 45472 Mülheim an der Ruhr Germany

**Keywords:** DFT Calculations, EPRSpectroscopy, Low-Valent Complexes, Magnesium, Radicals

## Abstract

In order to isolate a monometallic Mg radical, the precursor (Am)MgI⋅(CAAC) (**1**) was prepared (Am=*t*BuC(N‐DIPP)_2_, DIPP=2,6‐diisopropylphenyl, CAAC=cyclic (alkyl)(amino)carbene). Reduction of a solution of **1** in toluene with the reducing agent K/KI led to formation of a deep purple complex that rapidly decomposed. Ball‐milling of **1** with K/KI gave the low‐valent Mg^I^ complex (Am)Mg⋅(CAAC) (**2**) which after rapid extraction with pentane and crystallization was isolated in 15 % yield. Although a benzene solution of **2** decomposes rapidly to give Mg(Am)_2_ (**3**) and unidentified products, the radical is stable in the solid state. Its crystal structure shows planar trigonal coordination at Mg. The extremely short Mg−C distance of 2.056(2) Å indicates strong Mg−CAAC bonding. Calculations and EPR measurements show that most of the spin density is in a π* orbital located at the C−N bond in CAAC, leading to significant C−N bond elongation. This is supported by calculated NPA charges in **2**: Mg +1.73, CAAC −0.82. Similar metal‐to‐CAAC charge transfer was calculated for M^0^(CAAC)_2_ and [M^I^(CAAC)_2_^+^] (M=Be, Mg, Ca) complexes in which the metal charges range from +1.50 to +1.70. Although the spin density of the radical is mainly located at the CAAC ligand, complex **2** reacts as a low‐valent Mg^I^ complex: reaction with a I_2_ solution in toluene gave (Am)MgI⋅(CAAC) (**1**) as the major product.

## Graphical abstract

Ball‐milling of a magnesium iodide precursor with K/KI gave a mononuclear Mg radical which is stabilized by a CAAC ligand. Although stable in the crystalline state, the complex decomposed rapidly in solution. According to calculations and EPR spectroscopy, most of the spin density is localized on the CAAC ligand.

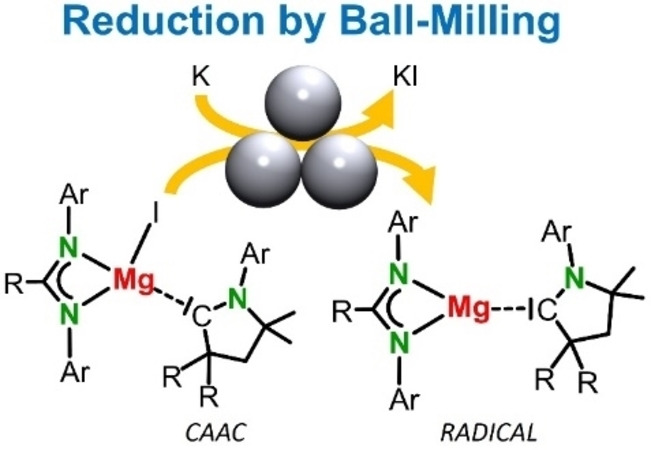

Prior to the isolation of stable molecular Mg^I^ complexes (**I**, **II** in Scheme 1),^[1]^ the low‐valent chemistry of Mg was restricted to unstable XMg⋅ radical species (X=H, Me, NC) detected in extraterrestial space or in a low‐temperature matrix.[^2^, ^3^, ^4^] These unstable halogenide radicals XMg⋅ (X=Cl, Br, I) received increased attention as intermediates during Grignard formation^[5]^ or highly reactive reagents for C−C bond coupling. The unusually high reducing power of Mg^0^/MgI_2_ mixtures^[6]^ is explained by formation of the IMg⋅ radical, the active reducing agent in the pinacol coupling.^[7]^ Like in **I** and **II**, these radicals have a strong tendency to form Mg−Mg bonds. Coupling of *in situ* prepared ClMg⋅ radicals to ClMg−MgCl is exothermic (calculated: −47 kcal mol^−1^) and occurs even below −60 °C.[^8^, ^9^] The Mg−Mg bond in **I** is described as a 2*s* ^1^–2*s* ^1^ interaction (HOMO) of circa 40–45 kcal mol^−1^.[^10^, ^11^, ^12^] In contrast to rapid disproportionation of Mg_2_Cl_2_ in MgCl_2_/Mg^0^(s) (Δ*H*=−22 kcal mol^−1^),^[8]^ the Mg−Mg bond in **I** is kinetically stabilized by bulky β‐diketiminate (BDI) ligands. Complex **I** is the first molecular complex with a Non‐Nuclear‐Attractor (NNA) directly on the Mg−Mg axis.^[13]^ This NNA functions as an electron reservoir for application of these dinuclear Mg^I^ complexes as soft reducing agents.^[14]^ Isolation of the much more reactive Mg^I^ radicals was recently formulated as one of the main future challenges.[^15^, ^16^]

**Scheme 1:**
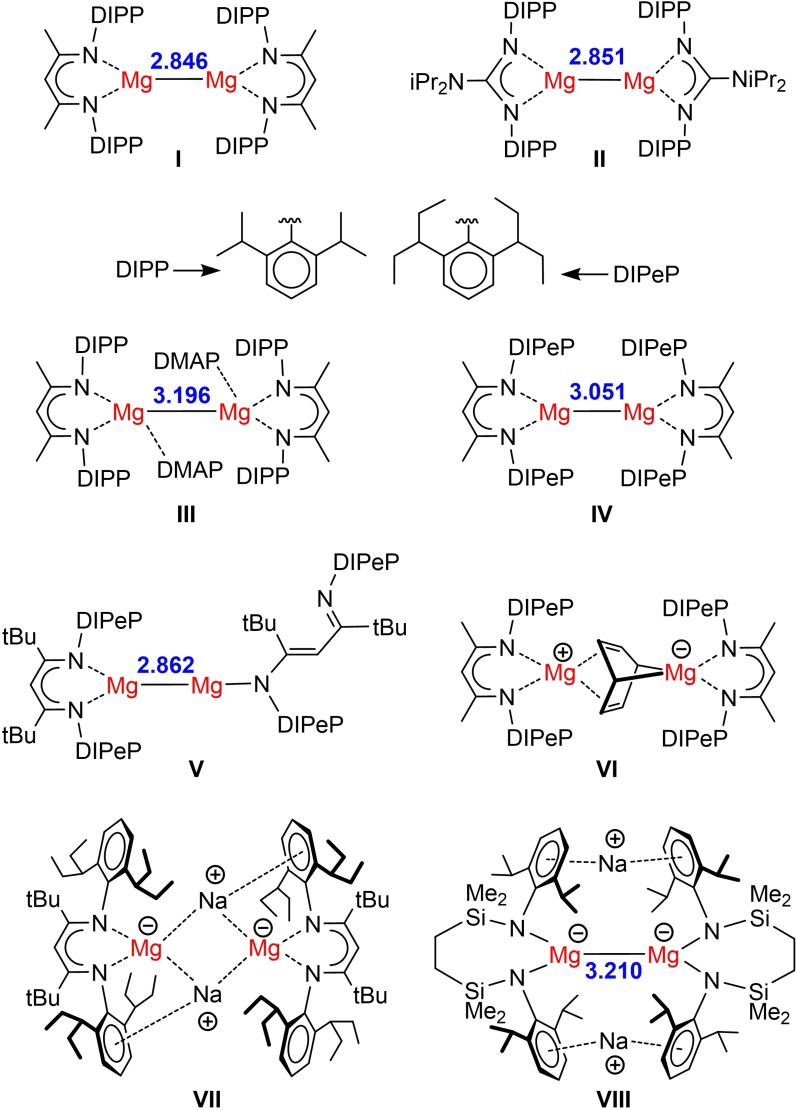
Low‐valent Mg complexes. In blue: Mg−Mg distance in Å. DMAP=4‐dimethylaminopyridine.

Solvation of the Mg centers in **I** by 4‐dimethylamino‐pyridine (**III**) did not cleave the Mg−Mg bond,^[17]^ but considerable Mg−Mg bond lengthening demonstrates its high flexibility.^[13]^ Attempts to cleave the Mg−Mg bond by increasing the ligand bulk led to significant stretching (**IV**)^[18]^ or Mg−N bond scission (**V**).^[12]^ Stabilization of *in situ* formed (BDI)Mg⋅ with the chelating ligand *N*,*N*,*N*′,*N*′‐tetramethylethylenediamine resulted in attack of the aromatic solvent (**VI**).^[18]^ Similar destruction of the aromatic solvent was found when the Mg−Mg bond was cleaved by UV‐irradiation.^[19]^ An attempt to trap the Mg^I^ radical in the cleft of a bulky carbazole ligand gave ligand reduction.^[20]^ Using a superbulky BDI ligand, we found that *in situ* formed (BDI)Mg⋅ is easily over‐reduced to give an unique closed‐shell Mg^0^ complex (**VII**).^[21]^ More recently, Hill and co‐workers reported a dianionic Mg^I^ complex with a very long, but intact Mg−Mg bond (**VIII**).^[22]^

These observations set the scene for the challenging isolation of highly reactive Mg^I^ radicals. Key to the isolation of such a species is a cyclic (alkyl)(amino)carbene ligand (CAAC), which is well‐known for stabilization of low‐valent metals.[^23^, ^24^, ^25^, ^26^] The Be‐CAAC contacts in Be^0^(CAAC)_2_, [Be^I^(CAAC)_2_^+^] and (CAAC‐H)Be^I^(CAAC) are described as a synergistic bond involving C→Be σ‐donation and strong Be→C π‐backdonation; CAAC‐H = CAAC+H^−^.[^24^, ^25^, ^26^] For this reason, we prepared a magnesium iodide precursor with a CAAC ligand (**1** in Scheme 2, Figure 1a). As known CAAC ligands are bulky, a relatively open Mg complex with an amidinate ligand (Am) of moderate bulk was chosen. Attempts to reduce (Am)MgI⋅(CAAC) (**1**) in toluene solution with a slight excess of K/KI^[27]^ led to an immediate color change to deep purple, indicative of the expected radical (Am)Mg⋅(CAAC) (**2**). However, rapid fading of the color suggested that the radical decomposed to closed‐shell products, also at low temperature. Earlier attempts to isolate a Mg^0^(CAAC)_2_ complex under low temperature conditions led to oxidative addition of Mg^0^ to a C−C bond in the CAAC ligand.^[28]^ This clearly illustrates the much higher reactivity of the more electropositive Mg^0^ species when compared to Be^0^(CAAC)_2_ which is stable up to 180–190 °C.^[24]^ The instability of **2** motivated a ball‐milling approach, an increasingly popular technique that recently also entered group 2 metal chemistry.[^29^, ^30^] Highly concentrated solid‐solid reactions are much faster than solution reactions and have the advantage to deliver the product in the solid state, in which it is “frozen” from further decomposition.

**Scheme 2:**
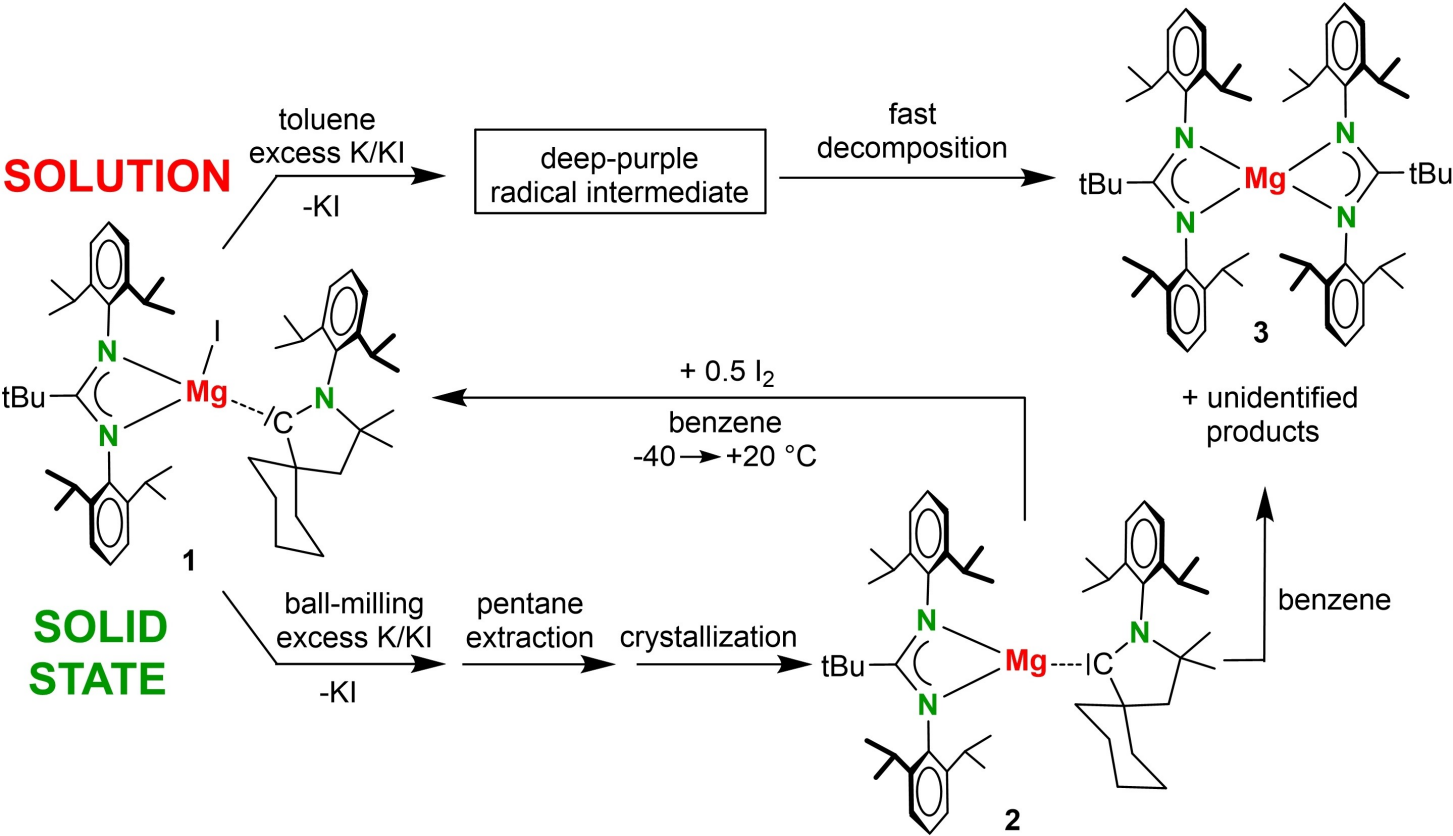
Top: Attempted reduction of **1** in toluene solution. Bottom: Synthesis of **2** by ball‐milling in the solid state and oxidation to **1** by addition of I_2_.

**Figure 1:**
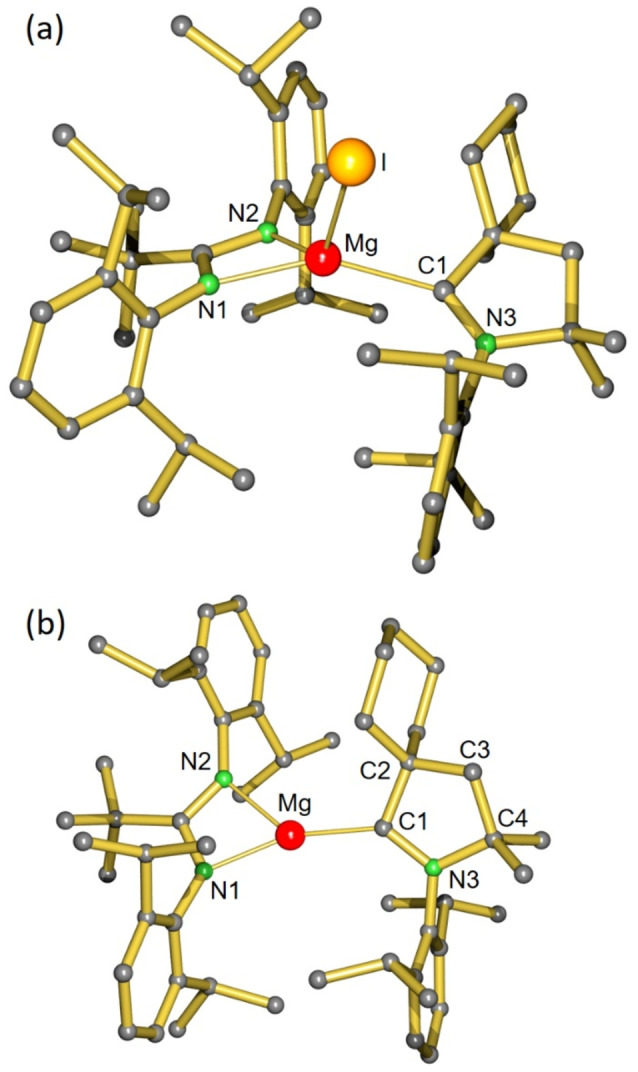
a) Crystal structure of **1**; H atoms omitted for clarity. Selected bond lengths [Å] and angles [°]: Mg–N1 2.104(2), Mg–N2 2.081(2), Mg–C1 2.264(2), Mg–I 2.7204(8), N1‐Mg‐N2 63.9(1), N1‐Mg‐C1 151.3(1), N2‐Mg‐C1 116.5(1), N1‐Mg‐I 108.3(1), N2‐Mg‐I 126.7(1). b) Crystal structure of **2**; H atoms omitted for clarity. Selected bond lengths [Å] and angles [°]: Mg–N1 2.037(1), Mg–N2 2.074(1), Mg–C1 2.056(2), N1‐Mg‐N2 64.9(1), N1‐Mg‐C1 159.5(1), N2‐Mg‐C1 135.4(1).

Ball‐milling the Mg iodide precursor **1** with K/KI led within two hours to formation of a deep purple powder which was extracted with pentane. Although the in situ formed product dissolves reasonably well in pentane, there is an immediate onset of crystallization. “Black” crystals of **2** were isolated in a yield of 15 %. The crystal structure of **2** (Figure 1b) revealed a mononuclear low‐valent Mg complex with a *N*,*N*‐chelating amidinate ligand and a nearly coplanar C‐bound CAAC ligand: N2−Mg−C1−C2=24.7(2)°. The metal coordination geometry is trigonal‐planar (sum of valence angles=359.9°). Differences in bulk between the DIPP and cyclohexyl groups in CAAC cause a large N1−Mg−C1 angle of 159.5(1)° and a small N2−Mg−C1 angle of 135.4(1)°. Although the Mg−N distances (average: 2.056 Å) are in a similar range as those in other three‐coordinate Mg amidinate complexes (2.012(1)–2.083(2) Å),[^31^, ^32^] the Mg−C bond length of 2.056(2) Å is extremely short. It is not only substantially shorter than that in **1** (2.265(2) Å), in which Mg is four‐coordinate, but also shorter than those in other three‐coordinate (R_2_N)_2_Mg⋅⋅⋅CAAC complexes (2.194(2)–2.299(2) Å) in which CAAC is only a strong σ‐donor.[^33^, ^34^] Like in low‐valent Be complexes,[^24^, ^25^, ^26^] the exceptionally short Mg−CAAC bond in **2** indicates significant π‐backbonding. A similar conclusion was reached for low‐valent Al^II^ and Ga^II^ radicals stabilized by CAAC.^[35]^

Although **2** crystallized from pentane, the product does not redissolve in this solvent. It is very well soluble in aromatics or ethers, forming intensely colored dark‐violet solutions. The broad UV/vis signal at 575 nm is at equal wavelength as that for Be^0^(CAAC)_2_.^[24]^ The ^1^H NMR spectrum of **2** in C_6_D_6_ consists of a set of broad signals, typical for paramagnetic complexes. The rapidly fading color indicates that the complex in solution decomposes at room temperature within one hour. Upon decomposition, the broad ^1^H NMR signals transformed in sharp signals among which those of the homoleptic Mg amidinate complex **3** (Figure S19). Complex **3** was isolated in 66 % yield and was fully characterized (Figure S13–S18 and S30) but further identification of decomposition products failed.

The structure of **2** calculated at the B3PW91/def2TZVP//def2SVP level (including GD3BJ dispersion correction) is in excellent agreement with the crystal structure (Figure S24). Notably, the exceptional short Mg−C distance of 2.056(2) Å was reproduced by calculation (2.035 Å). Geometry optimization without dispersion correction gave a Mg−C distance of 2.090 Å, indicating that its shortness can only partially be explained by ligand‐ligand attraction. It is more likely inherent to the interaction between the Mg radical species (Am)Mg⋅ and CAAC (Scheme 3a). Formation of complex **2** (Δ*H*=−53.5 kcal mol^−1^) is competitive with formation of a Mg−Mg bond (Δ*H*=−55.8 kcal mol^−1^), supporting the idea that Mg−Mg bond formation can be inhibited by a strong CAAC ligand. Atoms‐In‐Molecules (AIM) analysis shows a bond‐critical‐point (BCP) on the Mg−C axis with an electron density *ρ* of +0.060 e B^−3^ and a Laplacian **∇^2^** *ρ*(*r*) of +0.309 e B^−5^ (Scheme 3b). These values are higher than those for the Mg−N bonds. A small positive electron density and a larger positive Laplacian are indicators for electrostatic rather than covalent bonding. The contour plot of the Laplacian for (Am)Mg⋅ shows a concentration of electrons on the open coordination site of Mg. Bonding with CAAC results in depletion of electron density at Mg, but the lone‐pair of electrons at the carbene C atom is clearly polarized towards Mg when compared to free CAAC.

**Scheme 3:**
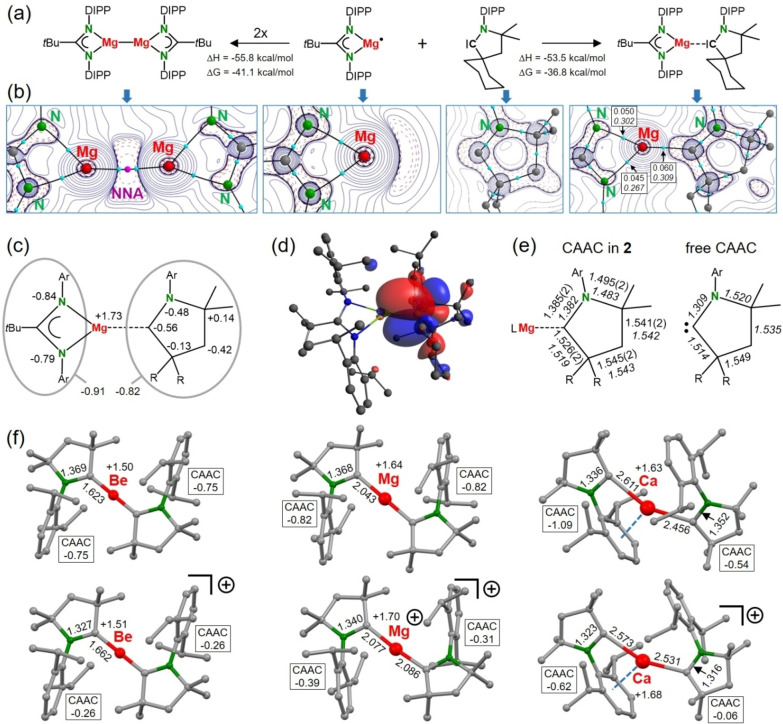
a) Enthalpies and free energies (298 K) for the dimerization of (Am)Mg⋅ and for complexation with CAAC; B3PW91/def2TZVP//def2SVP. b) Contour plots of the Laplacian **∇^2^** *ρ* showing areas of electron density concentration (dashed lines) and depletion (solid lines). The BCP's are shown in blue and the NNA in pink. Boxed numbers show the electron density *ρ* and the Laplacian **∇^2^** *ρ* (italic) in the BCP. c) NPA charges in (Am)Mg⋅(CAAC) (**2**). d) SOMO in (Am)Mg⋅(CAAC) (**2**). e) The geometry of CAAC in **2** compared to that of free CAAC. Experimental bond lengths in Å (italic numbers show calculated values). f) Calculated structures and NPA charges for M^0^(CAAC)_2_ and [M^I^(CAAC)_2_^+^] (M=Be, Mg, Ca).

These observations indicate considerable charge transfer from Mg to CAAC. Calculated NPA charges for **2** support this conclusion (Scheme 3c). The charge on Mg is +1.73, while amidinate and CAAC ligands carry charges of −0.91 and −0.82, respectively. Most of the electron density on the CAAC ligand is concentrated in the C−N unit. The SOMO in **2** is bonding with respect to Mg and C and has π* character at the C−N bond (Scheme 3d). This results in significant C−N bond elongation from 1.309 Å in free CAAC to 1.385(2) Å in **2**; see Scheme 3e. The other bonds in the CAAC ring are much less affected.

Charge transfer to CAAC and concomittant C−N bond elongation is not uncommon. In fact, it is the rationale for its property to stabilize low‐valent metals.^[36]^ Also in Be^0^(CAAC)_2_ and [Be^I^(CAAC)_2_^+^] considerable charge transfer from Be to the CAAC ligands was observed, but atomic charges have not been discussed.[^24^, ^25^, ^26^] For comparison, we calculated the NPA charges at the B3PW91/def2TZVP//def2SVP level (Scheme 3f). Be^0^(CAAC)_2_: Be+1.50, CAAC −0.75; [Be^I^(CAAC)_2_^+^]: Be+1.51, CAAC −0.26. The NPA charge on Be fits well with that calculated in BeMe_2_ (+1.49).^[37]^ For hypothetical complexes of the more electropositive Mg, a slightly larger charge transfer was calculated. Mg^0^(CAAC)_2_: Mg+1.64, CAAC −0.82; [Mg^I^(CAAC)_2_^+^]: Mg+1.70, CAAC −0.39/−0.31. Note that the charge of +1.73 on Mg in **2** is comparable and that C−N bond lengthening is proportional to the negative charge on CAAC. Similar complexes with the even more reducing metal Ca optimized to structures in which one of the aryl groups interacts with low‐valent Ca. In the extreme case of Ca^0^(CAAC)_2_, up to 1.09 *e* was transferred to CAAC of which 0.69 *e* is located on the aryl ring. Larger metals like Ca seem to prefer metal‐arene bonding like in Ca^0^(benzene)_3_.^[38]^

Electron transfer from metal to the CAAC ligand is supported by a spin density analysis. For the metals in [Be^I^(CAAC)_2_^+^], (CAAC‐H)Be(CAAC), Mulliken spin densities of 0.38 and 0.23 were reported (BP86‐D3(BJ)/def2SVP).[^25^, ^26^] Using the same method, the spin density on Mg in **2** is 0.19. However, with the more flexible basis set def2TZVP, all values drop considerably to 0.11, 0.07 and 0.11, respectively. Metal spin densities from a Natural‐Bond‐Orbital (NBO) analysis are less basis set dependend and gave for [Be^I^(CAAC)_2_^+^], (CAAC‐H)Be(CAAC) and **2** values of 0.11, 0.06 and 0.03, respectively (Table S3).

Electron paramagnetic resonance (EPR) spectra of **2** were measured at X‐band and simulated using an effective spin hamiltonian with hyperfine couplings in agreement with DFT calculations (Figure 2 and S20).[^39^, ^40^] The isotropic spectrum in a toluene solution at room temperature is dominated by a ^14^N hyperfine coupling pattern, with additional weak hyperfine satellites. According to DFT calculations, there is nontrivial hyperfine coupling at three positions: Mg (^25^Mg, I=5/2, 10.0 % nat. abund.), C1 (^13^C, I=1/2, 1.1 % nat. abund.) and N3 (^14^N, I=1, 99.6 % nat. abund.). The spectrum of **2** is well‐simulated using hyperfine coupling constants of ^14^N: 0.48 (0.37), ^25^Mg: 0.16 (0.25), ^13^C: 1.60 (1.46) (mT; DFT values are given parenthetically). Hyperfine coupling is a sensitive probe of spin localization, and the good agreement between the DFT and simulated couplings is interpreted as strong experimental support for the calculated spin density, which is mainly on CAAC (Tables S2 and S3), and the overall DFT electronic structure description of **2**. While the unpaired electron is mainly localized at the CAAC ligand, EPR shows that Mg clearly participates in the covalent bonding structure. Diamagnetic Be^0^(CAAC)_2_ is EPR‐silent,^[24]^ and the unpaired electron in the Be^I^(CAAC)_2_^+^ cation showed coupling to the two ^14^N nuclei in the CAAC ligands (0.34 mT) but not to ^9^Be (I=3/2, 100 % nat. abund.),^[25]^ whereas (CAAC‐H)Be(CAAC) shows coupling to ^9^Be (0.41 mT).^[26]^ The EPR signal for a recently reported Al^II^ radical stabilized by CAAC was interpreted as hyperfine coupling to ^14^N of CAAC (0.53 mT) and ^27^Al (0.95 mT, I=5/2, 100 % nat. abund.).^[35]^

**Figure 2:**
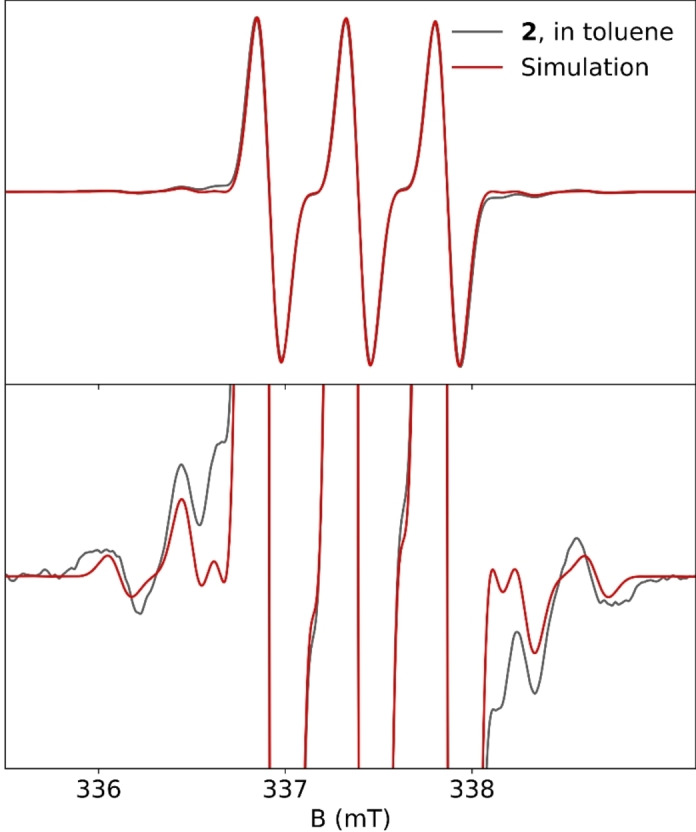
X‐band EPR spectrum of **2** in dilute toluene solution at 302 K (black), together with a spin hamiltonian simulation (red) at full‐scale (top) and enlarged 20× (bottom); see the Supporting Information for experimental and computational details.

Complex **2** can formally be seen as being constructed from a neutral (Am)Mg^I^ radical and a CAAC ligand. Charge analysis and EPR spectra demonstrate that a large part of the spin density is on the CAAC ligand and one could break‐down **2** also into a (Am)Mg^II^ cation and a CAAC radical anion. Neither formal assignment captures the real electronic structure, and metal oxidation states in such contexts are poorly defined. There are numerous examples where redox states are not clearly defined,[^41^, ^42^, ^43^] and a discussion on the metal oxidation state is often meaningless.^[44]^ Assuming the unpaired electron is involved in bonding and following formal IUPAC rules (bonding electrons are assigned to the most electronegative partner),^[45]^ the metal oxidation state in (Am)Mg⋅(CAAC) (**2**), but also that in Be^0^(CAAC)_2_ or the Be^I^(CAAC)_2_^+^ ion, should formally be +II, a value close to the calculated NPA charges in Scheme 3. Regardless of the assignment of the unpaired electron, these electron‐rich complexes react as reducing agents. Reactivity studies of **2** are limited by its poor stability in solution but preliminary investigations demonstrate that **2** reacts with I_2_ as a Mg^I^ synthon to give educt **1** (Scheme 2, Figure S23).

In summary, essential to the isolation of the strongly coloured low‐valent Mg radical **2** are its stabilization by a CAAC ligand and the application of ball‐milling in the reduction step. The Mg−CAAC bond of 2.056(2) Å is exceptionally short when compared to other Mg−CAAC bonds (2.19–2.30 Å), indicating strong bonding. EPR studies and DFT calculations show that the SOMO is mainly located at a π* orbital of the C−N bond in CAAC, resulting in significant C−N bond elongation. The NPA charge of +1.73 on Mg is similar to charges calculated for Mg in the hitherto unknown species Mg^0^(CAAC)_2_ (+1.64) or [Mg^I^(CAAC)_2_^+^] (+1.70). It is only slightly higher than the charge on Be in Be^0^(CAAC)_2_ (+1.50) and [Be^I^(CAAC)_2_^+^] (+1.51), both species with metal→CAAC π‐backdonation and C−N bond elongation. Complex **2** decomposes rapidly in solution, giving the homoleptic *bis*‐amidinate Mg complex Mg(Am)_2_ (**3**) and unidentified products related to decomposition of the CAAC ligand. Although the spin density is mainly located on the CAAC ligand, **2** reacts like a low‐valent Mg complex. In addition to comprehensive investigations on **2**, we are actively pursuing further applications of ball‐milling as a strategy to isolate low‐valent main group metal complexes.

## Conflict of interest

The authors declare no conflict of interest.

## Supporting information

As a service to our authors and readers, this journal provides supporting information supplied by the authors. Such materials are peer reviewed and may be re‐organized for online delivery, but are not copy‐edited or typeset. Technical support issues arising from supporting information (other than missing files) should be addressed to the authors.

Supporting Information

Supporting Information

Supporting Information

## Data Availability

The data that support the findings of this study are available in the Supporting Information of this article.
