# Supplementary material for: Access to a Labile Monomeric Magnesium Radical by Ball‐Milling
Source: Angew Chem Int Ed Engl. 2022 Feb 18;61(15):e202200511. doi: 10.1002/anie.202200511 (PMC9306460; doi:10.1002/anie.202200511)

checkCIF/PLATON report

Structure factors have been supplied for datablock(s) hasj211027c

THIS REPORT IS FOR GUIDANCE ONLY. IF USED AS PART OF A REVIEW PROCEDURE FOR PUBLICATION, IT SHOULD NOT REPLACE THE EXPERTISE OF AN EXPERIENCED CRYSTALLOGRAPHIC REFEREE.

No syntax errors found. CIF dictionary Interpreting this report

Datablock: hasj211027c

Bond precision: C-C = 0.0039 A Wavelength=1.54184

Cell:	a=10.51945(12)	b=23.6504(3)	c=22.9991(3)
	alpha=90	beta=97.9283(12)	gamma=90
Temperature:	160 K		

	Calculated	Reported
Volume	5667.24(12)	5667.24(12)
Space group	P 21/n	P 1 21/n 1
Hall group	-P 2yn	-P 2yn
Moiety formula	2(C52 H78 I Mg N3), C7 H8, 2(C6 H6)	C52 H78 I Mg N3, C6 H6, 0.5(C7 H8)
Sum formula	C123 H176 I2 Mg2 N6	C61.50 H88 I Mg N3
Mr	2041.12	1020.55
Dx,g cm-3	1.196	1.196
Z	2	4
Mu (mm-1)	4.829	4.829
F000	2172.0	2172.0
F000'	2176.09	
h,k,lmax	13,29,28	12,28,28
Nref	11189	10896
Tmin,Tmax	0.442,0.632	0.164,1.000
Tmin'	0.042	

Correction method= # Reported T Limits: Tmin=0.164 Tmax=1.000
AbsCorr = GAUSSIAN

Data completeness= 0.974 Theta(max)= 72.362

R(reflections)= 0.0398(9723) wR2(reflections)= 0.1076(10896)

S = 1.027 Npar= 772

The following ALERTS were generated. Each ALERT has the format
test-name_ALERT_alert-type_alert-level.
Click on the hyperlinks for more details of the test.

Alert level C

```
PLAT250_ALERT_2_C Large U3/U1 Ratio for Average U(i,j) Tensor ....        2.1 Note
PLAT250_ALERT_2_C Large U3/U1 Ratio for Average U(i,j) Tensor ....        2.4 Note
PLAT260_ALERT_2_C Large Average Ueq of Residue Including       C59      0.152 Check
PLAT911_ALERT_3_C Missing FCF Refl Between Thmin & STh/L=    0.600         42 Report
```

Alert level G

```
PLAT002_ALERT_2_G Number of Distance or Angle Restraints on AtSite         37 Note
PLAT003_ALERT_2_G Number of Uiso or Uij Restrained non-H Atoms ...         30 Report
PLAT042_ALERT_1_G Calc. and Reported Moiety Formula Strings Differ     Please Check
PLAT045_ALERT_1_G Calculated and Reported Z Differ by a Factor ...       0.50 Check
PLAT063_ALERT_4_G Crystal Size Possibly too Large for Beam Size ..       0.63 mm
PLAT172_ALERT_4_G The CIF-Embedded .res File Contains DFIX Records          1 Report
PLAT174_ALERT_4_G The CIF-Embedded .res File Contains FLAT Records          2 Report
PLAT176_ALERT_4_G The CIF-Embedded .res File Contains SADI Records          6 Report
PLAT178_ALERT_4_G The CIF-Embedded .res File Contains SIMU Records          4 Report
PLAT186_ALERT_4_G The CIF-Embedded .res File Contains ISOR Records          1 Report
PLAT300_ALERT_4_G Atom Site Occupancy of C59        Constrained at        0.5 Check
PLAT300_ALERT_4_G Atom Site Occupancy of C60        Constrained at        0.5 Check
PLAT300_ALERT_4_G Atom Site Occupancy of C61        Constrained at        0.5 Check
PLAT300_ALERT_4_G Atom Site Occupancy of C62        Constrained at        0.5 Check
PLAT300_ALERT_4_G Atom Site Occupancy of C63        Constrained at        0.5 Check
PLAT300_ALERT_4_G Atom Site Occupancy of C64        Constrained at        0.5 Check
PLAT300_ALERT_4_G Atom Site Occupancy of C65        Constrained at        0.5 Check
PLAT300_ALERT_4_G Atom Site Occupancy of H60        Constrained at        0.5 Check
PLAT300_ALERT_4_G Atom Site Occupancy of H61        Constrained at        0.5 Check
PLAT300_ALERT_4_G Atom Site Occupancy of H62        Constrained at        0.5 Check
PLAT300_ALERT_4_G Atom Site Occupancy of H63        Constrained at        0.5 Check
PLAT300_ALERT_4_G Atom Site Occupancy of H64        Constrained at        0.5 Check
PLAT300_ALERT_4_G Atom Site Occupancy of H65A       Constrained at        0.5 Check
PLAT300_ALERT_4_G Atom Site Occupancy of H65B       Constrained at        0.5 Check
PLAT300_ALERT_4_G Atom Site Occupancy of H65C       Constrained at        0.5 Check
PLAT301_ALERT_3_G Main Residue  Disorder .............(Resd  1  )        14% Note
PLAT302_ALERT_4_G Anion/Solvent/Minor-Residue Disorder (Resd  2  )       100% Note
PLAT302_ALERT_4_G Anion/Solvent/Minor-Residue Disorder (Resd  3  )       100% Note
PLAT302_ALERT_4_G Anion/Solvent/Minor-Residue Disorder (Resd  4  )       100% Note
PLAT304_ALERT_4_G Non-Integer Number of Atoms in ..... (Resd  2  )       7.50 Check
PLAT304_ALERT_4_G Non-Integer Number of Atoms in ..... (Resd  3  )       7.55 Check
PLAT304_ALERT_4_G Non-Integer Number of Atoms in ..... (Resd  4  )       4.45 Check
PLAT412_ALERT_2_G Short Intra XH3 .. XHn     H5A      ..H17A     .       2.14 Ang.
                                                      x,y,z  =      1_555 Check
PLAT412_ALERT_2_G Short Intra XH3 .. XHn     H14C     ..H5AA     .       2.04 Ang.
                                                      x,y,z  =      1_555 Check
PLAT412_ALERT_2_G Short Intra XH3 .. XHn     H25A     ..H32A     .       1.97 Ang.
                                                      x,y,z  =      1_555 Check
PLAT720_ALERT_4_G Number of Unusual/Non-Standard Labels .........         12 Note
PLAT789_ALERT_4_G Atoms with Negative _atom_site_disorder_group  #         15 Check
PLAT811_ALERT_5_G No ADDSYM Analysis: Too Many Excluded Atoms ....          ! Info
PLAT860_ALERT_3_G Number of Least-Squares Restraints ............        830 Note
PLAT912_ALERT_4_G Missing # of FCF Reflections Above STh/L=  0.600        242 Note
```

```
PLAT941_ALERT_3_G Average HKL Measurement Multiplicity ..........        1.9 Low
PLAT978_ALERT_2_G Number C-C Bonds with Positive Residual Density.         1 Info
```

```
   0 ALERT level A = Most likely a serious problem - resolve or explain
   0 ALERT level B = A potentially serious problem, consider carefully
   4 ALERT level C = Check. Ensure it is not caused by an omission or oversight
  42 ALERT level G = General information/check it is not something unexpected

   2 ALERT type 1 CIF construction/syntax error, inconsistent or missing data
   9 ALERT type 2 Indicator that the structure model may be wrong or deficient
   4 ALERT type 3 Indicator that the structure quality may be low
  30 ALERT type 4 Improvement, methodology, query or suggestion
   1 ALERT type 5 Informative message, check
```

It is advisable to attempt to resolve as many as possible of the alerts in all categories. Often the minor alerts point to easily fixed oversights, errors and omissions in your CIF or refinement strategy, so attention to these fine details can be worthwhile. In order to resolve some of the more serious problems it may be necessary to carry out additional measurements or structure refinements. However, the purpose of your study may justify the reported deviations and the more serious of these should normally be commented upon in the discussion or experimental section of a paper or in the "special_details" fields of the CIF. checkCIF was carefully designed to identify outliers and unusual parameters, but every test has its limitations and alerts that are not important in a particular case may appear. Conversely, the absence of alerts does not guarantee there are no aspects of the results needing attention. It is up to the individual to critically assess their own results and, if necessary, seek expert advice.

Publication of your CIF in IUCr journals

A basic structural check has been run on your CIF. These basic checks will be run on all CIFs submitted for publication in IUCr journals (*Acta Crystallographica*, *Journal of Applied Crystallography*, *Journal of Synchrotron Radiation*); however, if you intend to submit to *Acta Crystallographica Section C* or *E* or *IUCrData*, you should make sure that full publication checks are run on the final version of your CIF prior to submission.

Publication of your CIF in other journals

Please refer to the *Notes for Authors* of the relevant journal for any special instructions relating to CIF submission.

PLATON version of 13/07/2021; check.def file version of 13/07/2021

Datablock hasj211027c - ellipsoid plot

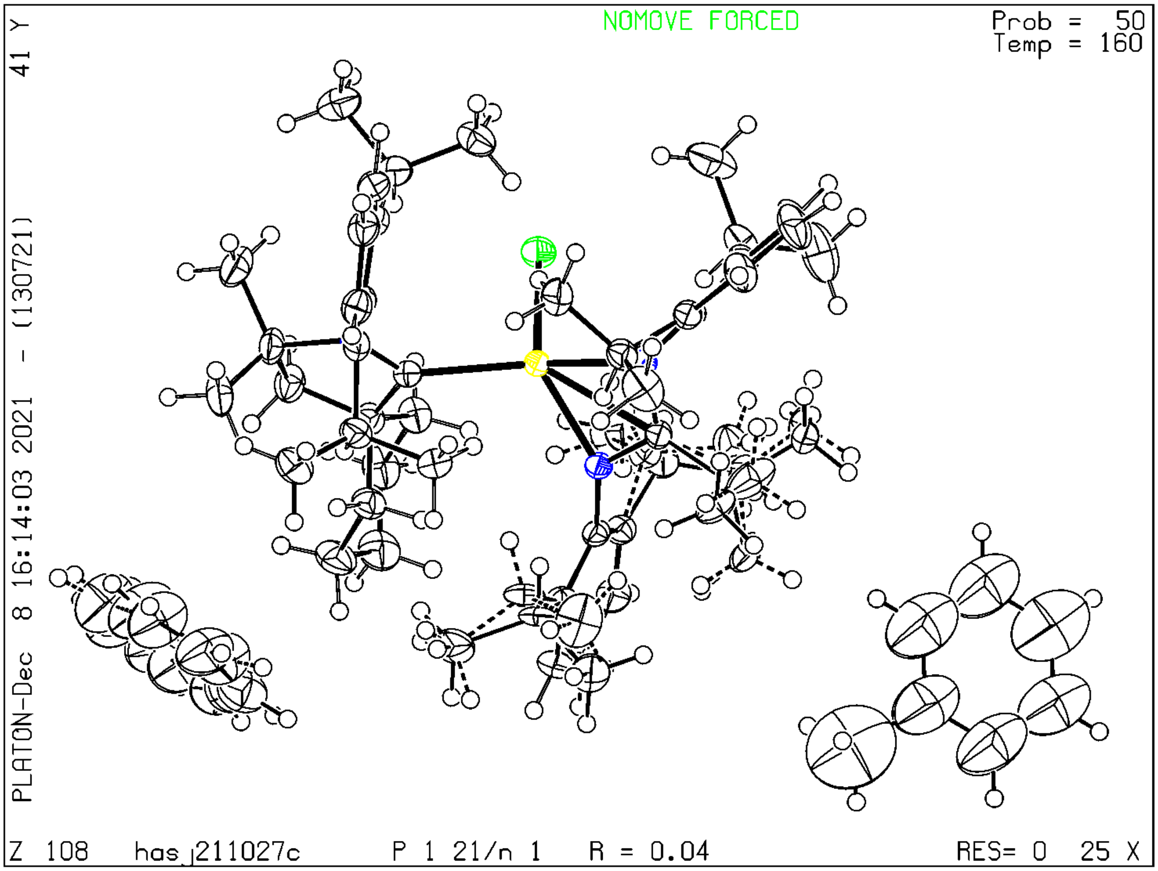

checkCIF/PLATON report

Structure factors have been supplied for datablock(s) hasj211029d

THIS REPORT IS FOR GUIDANCE ONLY. IF USED AS PART OF A REVIEW PROCEDURE FOR PUBLICATION, IT SHOULD NOT REPLACE THE EXPERTISE OF AN EXPERIENCED CRYSTALLOGRAPHIC REFEREE.

No syntax errors found.   CIF dictionary   Interpreting this report

Datablock: hasj211029d

```
Bond precision:   C-C = 0.0020 A          Wavelength=1.54184

Cell:              a=17.6227(3)      b=19.6722(4)      c=26.7713(5)
                   alpha=90          beta=90           gamma=90
Temperature:       100 K

                 Calculated                        Reported
Volume           9281.0(3)                         9281.0(3)
Space group      P b c a                           P b c a
Hall group       -P 2ac 2ab                        -P 2ac 2ab
Moiety formula   C52 H78 Mg N3                     C52 H78 Mg N3
Sum formula      C52 H78 Mg N3                     C52 H78 Mg N3
Mr               769.48                            769.48
Dx,g cm-3        1.101                             1.101
Z                8                                 8
Mu (mm-1)        0.591                             0.591
F000             3384.0                            3384.0
F000'            3393.05
h,k,lmax         21,24,33                          21,23,32
Nref             9177                              9019
Tmin,Tmax        0.908,0.953                       0.747,1.000
Tmin'            0.905

Correction method= # Reported T Limits: Tmin=0.747 Tmax=1.000
AbsCorr = GAUSSIAN

Data completeness= 0.983           Theta(max)= 72.314

                                                     wR2(reflections)=
R(reflections)= 0.0485( 7518)                        0.1277( 9019)

S = 1.049                    Npar= 522
```

The following ALERTS were generated. Each ALERT has the format
test-name_ALERT_alert-type_alert-level.
Click on the hyperlinks for more details of the test.

Alert level C

```
PLAT906_ALERT_3_C Large K Value in the Analysis of Variance ......      2.038 Check
PLAT911_ALERT_3_C Missing FCF Refl Between Thmin & STh/L=    0.600          8 Report
```

Alert level G

```
PLAT912_ALERT_4_G Missing # of FCF Reflections Above STh/L=  0.600        131 Note
PLAT941_ALERT_3_G Average HKL Measurement Multiplicity ..........         3.1 Low
PLAT978_ALERT_2_G Number C-C Bonds with Positive Residual Density.         15 Info
```

0 **ALERT level A** = Most likely a serious problem - resolve or explain
0 **ALERT level B** = A potentially serious problem, consider carefully
2 **ALERT level C** = Check. Ensure it is not caused by an omission or oversight
3 **ALERT level G** = General information/check it is not something unexpected

0 ALERT type 1 CIF construction/syntax error, inconsistent or missing data
1 ALERT type 2 Indicator that the structure model may be wrong or deficient
3 ALERT type 3 Indicator that the structure quality may be low
1 ALERT type 4 Improvement, methodology, query or suggestion
0 ALERT type 5 Informative message, check

It is advisable to attempt to resolve as many as possible of the alerts in all categories. Often the minor alerts point to easily fixed oversights, errors and omissions in your CIF or refinement strategy, so attention to these fine details can be worthwhile. In order to resolve some of the more serious problems it may be necessary to carry out additional measurements or structure refinements. However, the purpose of your study may justify the reported deviations and the more serious of these should normally be commented upon in the discussion or experimental section of a paper or in the "special_details" fields of the CIF. checkCIF was carefully designed to identify outliers and unusual parameters, but every test has its limitations and alerts that are not important in a particular case may appear. Conversely, the absence of alerts does not guarantee there are no aspects of the results needing attention. It is up to the individual to critically assess their own results and, if necessary, seek expert advice.

Publication of your CIF in IUCr journals

A basic structural check has been run on your CIF. These basic checks will be run on all CIFs submitted for publication in IUCr journals (*Acta Crystallographica*, *Journal of Applied Crystallography*, *Journal of Synchrotron Radiation*); however, if you intend to submit to *Acta Crystallographica Section C* or *E* or *IUCrData*, you should make sure that full publication checks are run on the final version of your CIF prior to submission.

Publication of your CIF in other journals

Please refer to the *Notes for Authors* of the relevant journal for any special instructions relating to CIF submission.

PLATON version of 13/07/2021; check.def file version of 13/07/2021

Datablock hasj211029d - ellipsoid plot

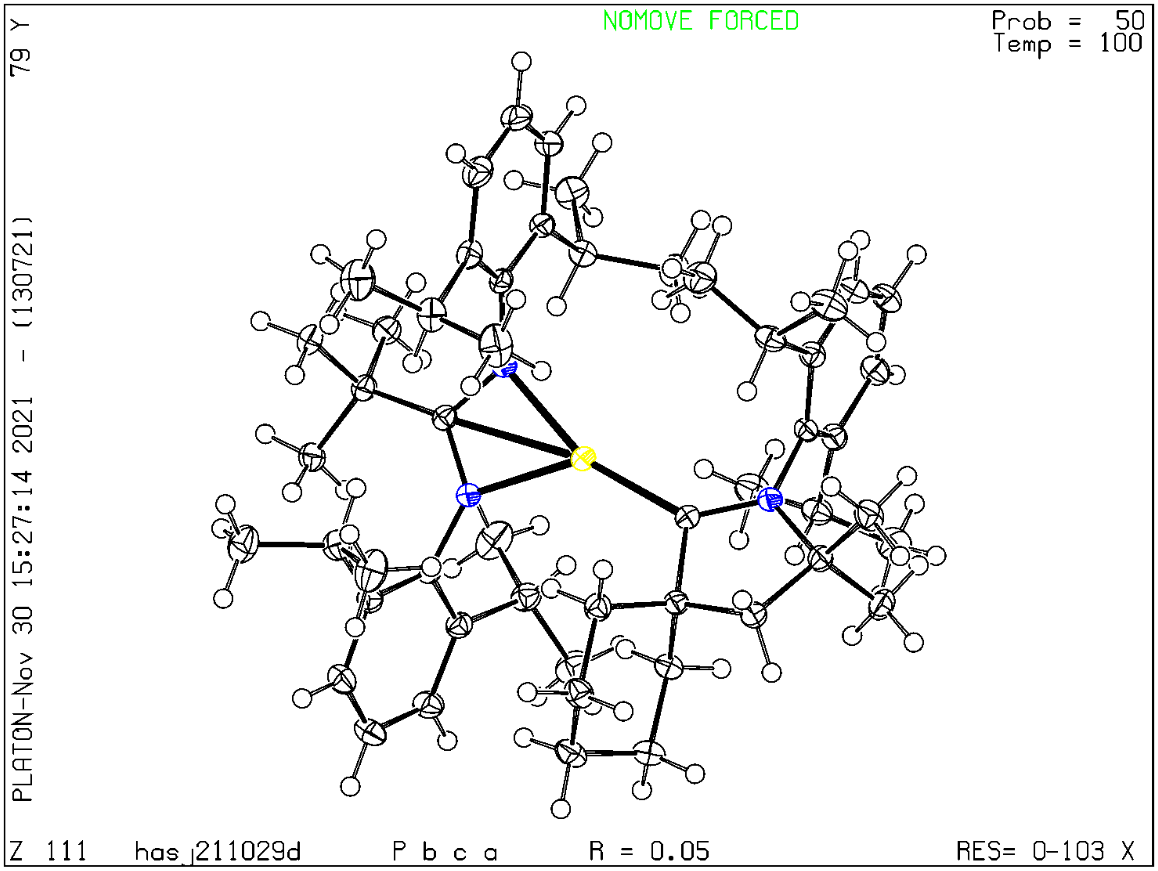

checkCIF/PLATON report

Structure factors have been supplied for datablock(s) hasj211201a_twin1_hklf5

THIS REPORT IS FOR GUIDANCE ONLY. IF USED AS PART OF A REVIEW PROCEDURE FOR PUBLICATION, IT SHOULD NOT REPLACE THE EXPERTISE OF AN EXPERIENCED CRYSTALLOGRAPHIC REFEREE.

No syntax errors found. CIF dictionary Interpreting this report

Datablock: hasj211201a_twin1_hklf5

```
Bond precision:   C-C = 0.0052 A          Wavelength=1.54184

Cell:            a=12.2331(5)       b=20.6715(7)      c=23.0680(11)
                 alpha=90.609(3)    beta=90.895(4)    gamma=98.901(3)
Temperature:     100 K

                  Calculated                          Reported
Volume            5761.9(4)                           5761.9(4)
Space group       P -1                                P -1
Hall group        -P 1                                -P 1
Moiety formula    C58 H86 Mg N4, C5 H12               C58 H86 Mg N4, C5 H12
Sum formula       C63 H98 Mg N4                       C63 H98 Mg N4
Mr                935.76                              935.76
Dx,g cm-3         1.079                               1.079
Z                 4                                   4
Mu (mm-1)         0.557                               0.557
F000              2064.0                              2064.0
F000'             2069.39
h,k,lmax          15,25,28                            15,25,28
Nref              22876                               25077
Tmin,Tmax         0.902,0.947                         0.916,0.961
Tmin'             0.877

Correction method= # Reported T Limits: Tmin=0.916 Tmax=0.961
AbsCorr = ANALYTICAL

Data completeness= 1.096           Theta(max)= 72.511

                                                        wR2(reflections)=
R(reflections)= 0.0605( 17948)                          0.1717( 25077)

S = 1.011                    Npar= 1433
```

The following ALERTS were generated. Each ALERT has the format
test-name_ALERT_alert-type_alert-level.
Click on the hyperlinks for more details of the test.

Alert level C

```
PLAT220_ALERT_2_C NonSolvent   Resd 1  C   Ueq(max)/Ueq(min) Range        4.6 Ratio
PLAT220_ALERT_2_C NonSolvent   Resd 2  C   Ueq(max)/Ueq(min) Range        3.3 Ratio
PLAT222_ALERT_3_C NonSolvent Resd 1  H   Uiso(max)/Uiso(min) Range        4.1 Ratio
PLAT230_ALERT_2_C Hirshfeld Test Diff for    C35      --C36      .        6.0 s.u.
PLAT340_ALERT_3_C Low Bond Precision on  C-C Bonds ...............    0.00521 Ang.
PLAT906_ALERT_3_C Large K Value in the Analysis of Variance ......      2.637 Check
PLAT911_ALERT_3_C Missing FCF Refl Between Thmin & STh/L=    0.600        147 Report
PLAT918_ALERT_3_C Reflection(s) with I(obs) much Smaller I(calc) .          2 Check
```

Alert level G

```
PLAT002_ALERT_2_G Number of Distance or Angle Restraints on AtSite         41 Note
PLAT003_ALERT_2_G Number of Uiso or Uij Restrained non-H Atoms ...         33 Report
PLAT072_ALERT_2_G SHELXL First  Parameter in WGHT  Unusually Large       0.11 Report
PLAT176_ALERT_4_G The CIF-Embedded .res File Contains SADI Records          3 Report
PLAT178_ALERT_4_G The CIF-Embedded .res File Contains SIMU Records          5 Report
PLAT187_ALERT_4_G The CIF-Embedded .res File Contains RIGU Records          6 Report
PLAT301_ALERT_3_G Main Residue  Disorder ..............(Resd  1  )        14% Note
PLAT301_ALERT_3_G Main Residue  Disorder ..............(Resd  2  )         3% Note
PLAT302_ALERT_4_G Anion/Solvent/Minor-Residue Disorder (Resd  4  )       100% Note
PLAT302_ALERT_4_G Anion/Solvent/Minor-Residue Disorder (Resd  5  )       100% Note
PLAT304_ALERT_4_G Non-Integer Number of Atoms in ..... (Resd  4  )       8.74 Check
PLAT304_ALERT_4_G Non-Integer Number of Atoms in ..... (Resd  5  )       8.26 Check
PLAT412_ALERT_2_G Short Intra XH3 .. XHn     H61A     ..H74F     .       2.05 Ang.
                                                       x,y,z  =     1_555 Check
PLAT412_ALERT_2_G Short Intra XH3 .. XHn     H90C     ..H127     .       2.09 Ang.
                                                       x,y,z  =     1_555 Check
PLAT412_ALERT_2_G Short Intra XH3 .. XHn     H107     ..H12D     .       2.14 Ang.
                                                       x,y,z  =     1_555 Check
PLAT412_ALERT_2_G Short Intra XH3 .. XHn     H49      ..H55C     .       2.14 Ang.
                                                       x,y,z  =     1_555 Check
PLAT412_ALERT_2_G Short Intra XH3 .. XHn     H49      ..H55F     .       2.09 Ang.
                                                       x,y,z  =     1_555 Check
PLAT860_ALERT_3_G Number of Least-Squares Restraints .............        834 Note
PLAT870_ALERT_4_G ALERTS Related to Twinning Effects Suppressed ..          ! Info
PLAT910_ALERT_3_G Missing # of FCF Reflection(s) Below Theta(Min).          2 Note
PLAT912_ALERT_4_G Missing # of FCF Reflections Above STh/L=  0.600        744 Note
PLAT931_ALERT_5_G CIFcalcFCF  Twin Law ( 0 0 1)         Est.d BASF       0.23 Check
PLAT933_ALERT_2_G Number of OMIT Records in Embedded .res File ...          7 Note
PLAT941_ALERT_3_G Average HKL Measurement Multiplicity ...........        1.1 Low
```

0 **ALERT level A** = Most likely a serious problem - resolve or explain
0 **ALERT level B** = A potentially serious problem, consider carefully
8 **ALERT level C** = Check. Ensure it is not caused by an omission or oversight
24 **ALERT level G** = General information/check it is not something unexpected

0 ALERT type 1 CIF construction/syntax error, inconsistent or missing data

```
 12 ALERT type 2 Indicator that the structure model may be wrong or deficient
 10 ALERT type 3 Indicator that the structure quality may be low
  9 ALERT type 4 Improvement, methodology, query or suggestion
  1 ALERT type 5 Informative message, check
```

It is advisable to attempt to resolve as many as possible of the alerts in all categories. Often the minor alerts point to easily fixed oversights, errors and omissions in your CIF or refinement strategy, so attention to these fine details can be worthwhile. In order to resolve some of the more serious problems it may be necessary to carry out additional measurements or structure refinements. However, the purpose of your study may justify the reported deviations and the more serious of these should normally be commented upon in the discussion or experimental section of a paper or in the "special_details" fields of the CIF. checkCIF was carefully designed to identify outliers and unusual parameters, but every test has its limitations and alerts that are not important in a particular case may appear. Conversely, the absence of alerts does not guarantee there are no aspects of the results needing attention. It is up to the individual to critically assess their own results and, if necessary, seek expert advice.

Publication of your CIF in IUCr journals

A basic structural check has been run on your CIF. These basic checks will be run on all CIFs submitted for publication in IUCr journals (*Acta Crystallographica*, *Journal of Applied Crystallography*, *Journal of Synchrotron Radiation*); however, if you intend to submit to *Acta Crystallographica Section C* or *E* or *IUCrData*, you should make sure that full publication checks are run on the final version of your CIF prior to submission.

Publication of your CIF in other journals

Please refer to the *Notes for Authors* of the relevant journal for any special instructions relating to CIF submission.

PLATON version of 13/07/2021; check.def file version of 13/07/2021

Datablock hasj211201a_twin1_hklf5 - ellipsoid plot

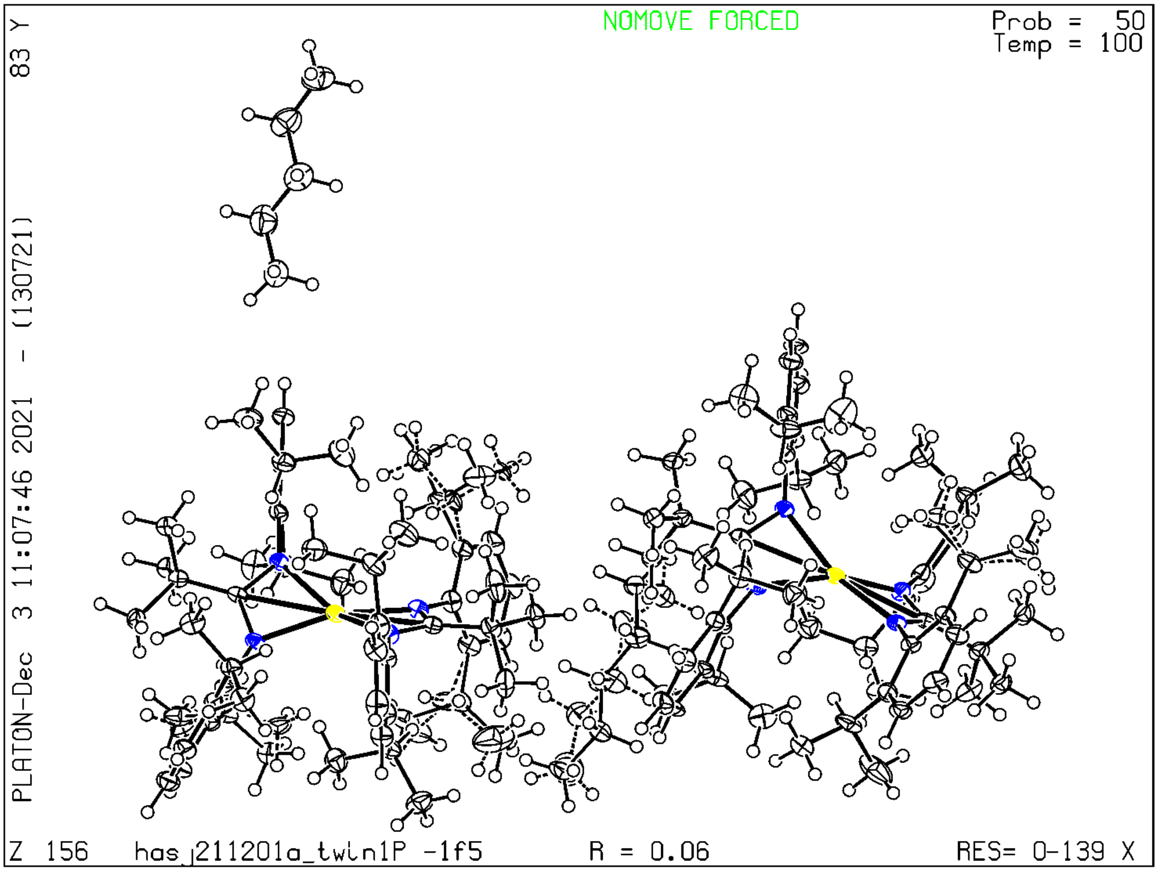

checkCIF/PLATON report

Structure factors have been supplied for datablock(s) hasj211119a

THIS REPORT IS FOR GUIDANCE ONLY. IF USED AS PART OF A REVIEW PROCEDURE FOR PUBLICATION, IT SHOULD NOT REPLACE THE EXPERTISE OF AN EXPERIENCED CRYSTALLOGRAPHIC REFEREE.

No syntax errors found. CIF dictionary Interpreting this report

Datablock: hasj211119a

```
Bond precision:   C-C = 0.0035 A          Wavelength=0.71073

Cell:           a=9.9240(3)        b=12.6128(4)      c=15.1493(5)
                alpha=104.763(3)   beta=107.472(3)   gamma=104.936(3)
Temperature:    100 K

                 Calculated                       Reported
Volume           1630.16(11)                      1630.17(9)
Space group      P -1                             P -1
Hall group       -P 1                             -P 1
Moiety formula   C58 H86 I2 Mg2 N4, C6 H14        C58 H86 I2 Mg2 N4, C6 H14
Sum formula      C64 H100 I2 Mg2 N4               C64 H100 I2 Mg2 N4
Mr               1227.90                          1227.89
Dx,g cm-3        1.251                            1.251
Z                1                                1
Mu (mm-1)        1.022                            1.022
F000             642.0                            642.0
F000'            641.29
h,k,lmax         13,17,21                         13,17,21
Nref             9309                             8212
Tmin,Tmax        0.621,0.699                      0.295,1.000
Tmin'            0.479

Correction method= # Reported T Limits: Tmin=0.295 Tmax=1.000
AbsCorr = GAUSSIAN

Data completeness= 0.882            Theta(max)= 29.767

R(reflections)= 0.0334( 7525)                      wR2(reflections)=
                                                   0.0888( 8212)
S = 1.066                   Npar= 337
```

The following ALERTS were generated. Each ALERT has the format
test-name_ALERT_alert-type_alert-level.
Click on the hyperlinks for more details of the test.

Alert level C

```
PLAT094_ALERT_2_C Ratio of Maximum / Minimum Residual Density ....       2.73 Report
```

Alert level G

```
PLAT002_ALERT_2_G Number of Distance or Angle Restraints on AtSite          3 Note
PLAT063_ALERT_4_G Crystal Size Possibly too Large for Beam Size ..       0.71 mm
PLAT152_ALERT_1_G The Supplied and Calc. Volume s.u. Differ by ...          2 Units
PLAT154_ALERT_1_G The s.u.'s on the Cell Angles are Equal ..(Note)      0.003 Degree
PLAT172_ALERT_4_G The CIF-Embedded .res File Contains DFIX Records          1 Report
PLAT176_ALERT_4_G The CIF-Embedded .res File Contains SADI Records          1 Report
PLAT860_ALERT_3_G Number of Least-Squares Restraints ............           4 Note
PLAT910_ALERT_3_G Missing # of FCF Reflection(s) Below Theta(Min).          3 Note
PLAT912_ALERT_4_G Missing # of FCF Reflections Above STh/L=  0.600       1094 Note
PLAT941_ALERT_3_G Average HKL Measurement Multiplicity ..........         3.5 Low
PLAT978_ALERT_2_G Number C-C Bonds with Positive Residual Density.          6 Info
```

0 **ALERT level A** = Most likely a serious problem - resolve or explain
0 **ALERT level B** = A potentially serious problem, consider carefully
1 **ALERT level C** = Check. Ensure it is not caused by an omission or oversight
11 **ALERT level G** = General information/check it is not something unexpected

2 ALERT type 1 CIF construction/syntax error, inconsistent or missing data
3 ALERT type 2 Indicator that the structure model may be wrong or deficient
3 ALERT type 3 Indicator that the structure quality may be low
4 ALERT type 4 Improvement, methodology, query or suggestion
0 ALERT type 5 Informative message, check

It is advisable to attempt to resolve as many as possible of the alerts in all categories. Often the minor alerts point to easily fixed oversights, errors and omissions in your CIF or refinement strategy, so attention to these fine details can be worthwhile. In order to resolve some of the more serious problems it may be necessary to carry out additional measurements or structure refinements. However, the purpose of your study may justify the reported deviations and the more serious of these should normally be commented upon in the discussion or experimental section of a paper or in the "special_details" fields of the CIF. checkCIF was carefully designed to identify outliers and unusual parameters, but every test has its limitations and alerts that are not important in a particular case may appear. Conversely, the absence of alerts does not guarantee there are no aspects of the results needing attention. It is up to the individual to critically assess their own results and, if necessary, seek expert advice.

Publication of your CIF in IUCr journals

A basic structural check has been run on your CIF. These basic checks will be run on all CIFs submitted for publication in IUCr journals (*Acta Crystallographica*, *Journal of Applied Crystallography*, *Journal of Synchrotron Radiation*); however, if you intend to submit to *Acta Crystallographica Section C* or *E* or *IUCrData*, you should make sure that full publication checks are run on the final version of your CIF prior to submission.

Publication of your CIF in other journals

Please refer to the *Notes for Authors* of the relevant journal for any special instructions relating to CIF submission.

PLATON version of 13/07/2021; check.def file version of 13/07/2021

Datablock hasj211119a - ellipsoid plot

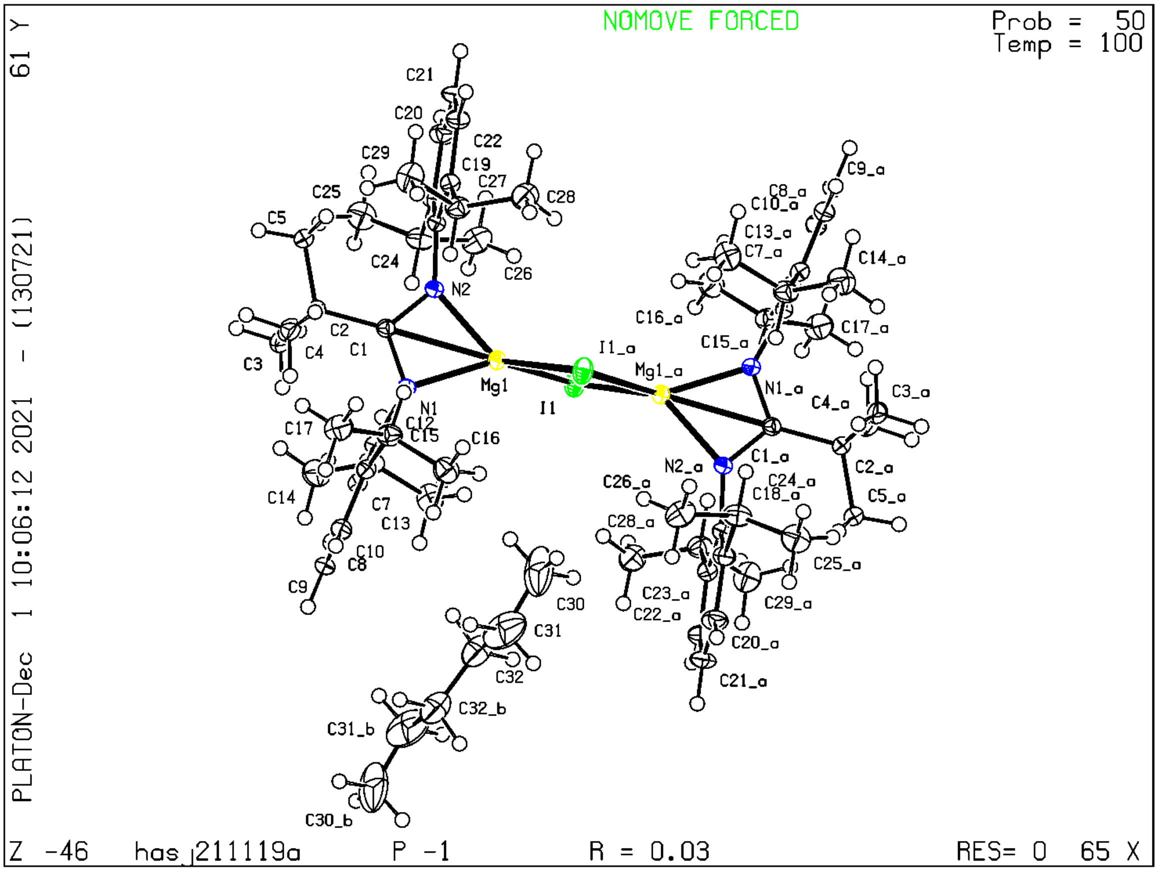

Supplement: Supplementary file 1 — Supporting Information [file ANIE-61-0-s001.pdf]
