# Supplementary material for: Access to a Labile Monomeric Magnesium Radical by Ball‐Milling
Source: Angew Chem Int Ed Engl. 2022 Feb 18;61(15):e202200511. doi: 10.1002/anie.202200511 (PMC9306460; doi:10.1002/anie.202200511)
Supplement: Supplementary file 3 — Supporting Information [file ANIE-61-0-s002.pdf]

Angewandte Chemie
Eine Zeitschrift der Gesellschaft Deutscher Chemiker

Supporting Information

Access to a Labile Monomeric Magnesium Radical by Ball-Milling

*D. Jędrzkiewicz, J. Mai, J. Langer, Z. Mathe, N. Patel, S. DeBeer, S. Harder**

Supporting Information

Contents

Contents	Page
**1. Supporting Experimental Data**	S2
**1.1 General experimental procedures**	S2
**1.2 Syntheses**	S3
**1.3 NMR spectroscopy characterization**	S5
**1.4 Stability of and NMR spectra of (Am)Mg·(CAAC) (2)**	S14
**1.5 EPR, IR and UV spectra of (Am)Mg·(CAAC) (2)**	S15
**1.6 Quenching (Am)Mg·(CAAC) (2) with iodine**	S18
**1.7 DFT calculations**	S19
**1.8 Crystal structure determinations**	S31
**2. References**	S36

1. Supporting Experimental Data

1.1 General experimental procedures

All experiments were conducted in dry glassware under an inert nitrogen or argon atmosphere by applying standard Schlenk techniques or gloveboxes (MBraun) using freshly dried and degassed solvents. Hexanes, pentanes, benzene and toluene were degassed with nitrogen, dried over a column with activated aluminum oxide (Innovative Technology, Pure Solv 400-4-MD, Solvent Purification System) and then stored under inert atmosphere over molecular sieves (3 Å). Deuterated benzene (C_6D_6) and toluene-d_8 were purchased from Deutero GmbH, degassed and dried over molecular sieves (3 Å) and stored under an inert atmosphere. Following reagents were obtained commercially and used without further purification: di-*n*-butyl magnesium solution 0.5 M in heptane (Sigma Aldrich), potassium (chunks, washed with hexane, 98% metal basis, Sigma Aldrich) and N_2 (Linde, 4N). Iodine (Sigma Aldrich, 99.99%) was sublimed and stored under nitrogen atmosphere. The following compounds were prepared according to literature procedures: AmH,[S1] CAAC carbene,[S2] K/KI.[S3] The ball-mill used was a ULTRA-TURRAX® Tube Drive P control from IKA. Mechanochemical reactions were performed in 20 mL polypropylene vessel with three stainless steel balls (diameter: 5 mm, weight: 0.52 g, type: AISI 304).

NMR spectra were measured on Bruker Avance III H 400 MHz and Bruker Avance III HD 600 MHz NMR spectrometers. Chemical shifts (δ) are denoted in ppm (parts per million) and coupling constants in Hz (Hertz). ^{1}H and ^{13}C NMR spectra were referenced to the solvent residual signal ($SiMe_4$ = 0 ppm). Signal multiplicities are described using common abbreviations: s (singlet), d (doublet), t (triplet), q (quartet), quint (quintet), m (multiplet) and br (broad). Elemental analysis was performed with a Hekatech Eurovector EA3000 analyzer and at Kolbe Microanalytical Laboratory (Mülheim/Ruhr, Germany). UV-vis spectra were recorded with an Agilent Technologies Cary 60 UV-VIS spectrophotometer. IR vibrational spectra were recorded neat in the solid state on a Bruker Alpha II Platinum ATR. All crystal structures have been measured on a SuperNova (Agilent) diffractometer with dual Cu and Mo microfocus sources and an Atlas S2 detector. Electron paramagnetic resonance (EPR) spectroscopy was performed using a Magnettech MS5000 spectrometer equipped with a TC H04 cryostat operated with Freiberg Instruments ESRStudio 1.74.3 software. The microwave frequency was 9.5 GHz, the modulation frequency was 100 kHz, the modulation amplitude was 0.01 mT, and the sweep time was 20 s. The microwave power was 1 mW, except where otherwise noted.

1.2 Syntheses

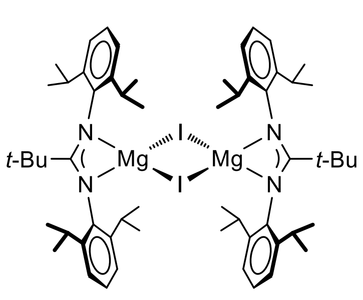

[(Am)MgI]$_2$ AmH (4.21 g, 10.0 mmol) was dissolved in hexane (50 mL) and 20 mL of 0.5 M (*n*-Bu)$_2$Mg in hexanes (10.0 mmol) was added slowly while stirring at room temperature. The mixture was stirred for an additional 5h. After addition of 2.54 g (10.0 mmol) of iodine, the mixture was stirred overnight. The volume of the mixture was reduced to 40 mL and placed at -20 °C overnight. A first crop of block-like crystals suitable for X-ray diffraction analysis was isolated by decanting the supernatant and washing the crystals with a minimal amount of cold hexanes (-20 °C, 2 x 10 mL). Subsequent drying in vacuo gave [(Am)MgI]$_2$ as a colorless powder. Further crops of crystals were obtained by repeated concentration of the mother liquor, isolation and drying of the crystals. Overall yield: 4.79 g, 4.19 mmol, 84%.

^{1}H NMR (600.13 MHz, C_6D_6, 298K): δ = 7.08 – 7.00 (m, 12H, Ar**H**), 3.61 (p, ^3J = 6.9 Hz, 8H, C**H**$^{i\text{-Pr}}$), 1.30 (d, ^3J = 6.9 Hz, 12H, C**H$_3$**$^{i\text{-Pr}}$), 1.22 (d, ^3J = 6.9 Hz, 12H, C**H$_3$**$^{i\text{-Pr}}$), 0.93 (s, 18H, C**H$_3$**$^{t\text{-Bu}}$) ppm.

^{13}C NMR (150.92 MHz, C_6D_6, 298K): δ = 181.3 ((*t*-Bu)**C**), 143.0 (Ar**C**-C), 142.9 (Ar**C**-N), 124.8 (Ar**C**H), 123.5 (Ar**C**H), 44.0 (**C**(CH$_3$)$_3$), 30.4 C(**C**H$_3$)$_3$), 28.8 (**C**H(CH$_3$)$_2$), 28.0 (CH(**C**H$_3$)$_2$), 22.5 (CH(**C**H$_3$)$_2$) ppm.

Elemental analysis: Calculated for $C_{58}H_{86}N_4Mg_2I_2$ (M = 1141.78 g/mol): C 61.01; H 7.59; N 4.91 %. Found: C 60.98; H 7.53; N 4.93 %.

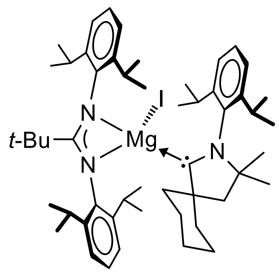

(Am)MgI·(CAAC) (1) [(Am)MgI]$_2$ (0.57 g, 0.50 mmol) and CAAC carbene (0.33 g, 1.0 mmol) were dissolved in benzene (20 mL) and stirred at room temperature for 2h. The solvent was removed in vacuo and the resulting off-white solid was washed with a minimal amount of cold pentane (3x 1 mL) and evaporated to dryness yielding (Am)MgI·(CAAC) as a white powder (yield: 0.82 g, 0.92 mmol, 92%). Colorless block-like crystals suitable for X-ray diffraction analysis were obtained from a sample dissolved in the mixture of benzene, toluene and hexanes (10/1/10) at room temperature.

^{1}H NMR (600.13 MHz, C_6D_6, 298K): δ = 7.22 (d, ^3J = 7.6 Hz, 2H, Ar**H**), 7.10 (t, ^3J = 7.6 Hz, 2H, Ar**H**), 7.03 (d, ^3J = 7.6 Hz, 2H, Ar**H**), 6.95 (t, ^3J = 7.6 Hz, 1H, Ar**H**), 6.85 (d, ^3J = 7.6 Hz, 2H, Ar**H**), 4.29 (p, ^3J = 6.7 Hz, 2H, C**H**$^{i\text{-Pr}}$), 3.36 (p, ^3J = 6.9 Hz, 2H, C**H**$^{i\text{-Pr}}$), 3.15 (p, ^3J = 6.7 Hz, 2H, C**H**$^{i\text{-Pr}}$), 1.97 (br s, 2H,C**H$_2$**^{Cy}), 1.68 (d, ^3J = 6.7 Hz, 6H, C**H$_3$**$^{i\text{-Pr}}$), 1.53 (d, ^3J = 6.7 Hz, 6H,C**H$_3$**$^{i\text{-Pr}}$), 1.49 (br d, ^3J = 12.9 Hz, 2H, C**H$_2$**^{Cy}), 1.44 (s, 2H, C**H$_2$**^{caac}),), 1.35 (d, ^3J = 6.8 Hz, 6H,C**H$_3$**$^{i\text{-Pr}}$), 1.27 – 1.19 (m, 4H, C**H$_2$**^{Cy}), 1.15 (d, ^3J = 6.7 Hz, 6H,C**H$_3$**$^{i\text{-Pr}}$), 1.06 (d, ^3J = 6.7 Hz, 6H,C**H$_3$**$^{i\text{-Pr}}$), 1.04 (s, 9H, C**H$_3$**$^{t\text{-Bu}}$), 1.01 – 0.95 (m, 3H, C**H$_2$**^{Cy}), 0.85 (s, 6H, C**H$_3$**$^{i\text{-Pr}}$), 0.74 (br s, 6H, C**H$_3$**^{caac}), 0.39 – 0.29 (m, 1H, C**H$_2$**^{Cy}) ppm.

^{13}C NMR (150.92 MHz, C_6D_6, 298K): δ = 261.6 (**C**^{carbene}), 178.6 ((*t*-Bu)**C**), 145.8 (Ar**C**), 145.4 (Ar**C**), 144.8 (Ar**C**), 143.0 (Ar**C**), 135.5 (Ar**C**), 129.7 (Ar**C**H), 125.8 (Ar**C**H), 124.8 (Ar**C**H), 124.2 (Ar**C**H), 122.6 (Ar**C**H), 82.8 (**C**^{Cy}), 63.9 (**C**(CH_3)$_2$), 44.5 (**C**H_2^{caac}), 43.4 (**C**(CH_3)$_3$), 39.2 (**C**H_2^{Cy}), 31.5 C(**C**H_3)$_3$), 29.7 (CH(**C**H_3)$_2$), 29.6 (CH(**C**H_3)$_2$), 29.5 (CH(**C**H_3)$_2$), 28.8 (**C**H(CH_3)$_2$), 28.3 (**C**H(CH_3)$_2$), 25.4 (**C**H(CH_3)$_2$), 24.6 (CH(**C**H_3)$_2$), 24.1 (**C**H_2^{Cy}), 23.2 (CH(**C**H_3)$_2$), 22.5 (**C**H_2^{Cy}) ppm.

Elemental analysis: Calculated for $C_{52}H_{78}N_3MgI$ (M = 896.43 g/mol): C 69.67; H 8.77; N 4.69 %. Found: C 69.73; H 8.94; N 4.59 %.

(Am)Mg·(CAAC) (2) (Am)MgI·(CAAC) (179 mg, 0.20 mmol) and K/KI (5 w%, 312 mg, 0.40 mmol) were placed in a vessel equipped with 3 stainless steel balls and ground over 2h with ball mill speed: 3600 rpm (60 Hz). Resulting dark blue powder was extracted with cold pentane (-20 °C, 15 mL). The pentane extract was concentrated to 5 mL and placed at -40 °C. Leaving it standing at this temperature overnight gave black block-like crystals of (Am)Mg·(CAAC) suitable for X-ray diffraction analysis that were isolated by decantation, washed with cold pentane (-40 °C, 1x 1 mL) and dried in vacuo (yield: 23 mg, 0.030 mmol, 15%). Attempts to isolate further crops of crystals failed due to decomposition of the complex into (Am)$_2$Mg (**3**) and other unidentified products. These crops are always contaminated with complex **3**.

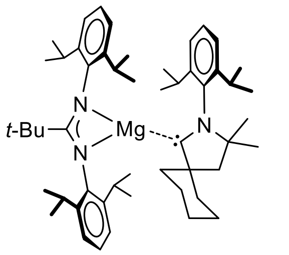

Elemental analysis: Calculated for $C_{52}H_{78}N_3Mg$ (M = 769.52 g/mol): C 81.16; H 10.22; N 5.46; Mg 3.16 %. Found: C 80.87; H 10.17; N 5.41; Mg 3.14 %.

(Am)$_2$Mg (3) (Am)MgI·(CAAC) (179 mg, 0.20 mmol) and K/KI (5 w%, 312 mg, 0.40 mmol) were suspended in toluene (50 mL) and stirred at room temperature for 24h. The solvent was removed in *vacuo* and the resulting solid was extracted with pentane (15 mL). The volume of the extract was concentrated to 5 mL and placed at -40 °C. Leaving it standing at this temperature over 4 days gave colorless block crystals of (Am)$_2$Mg suitable for X-ray diffraction analysis that were isolated by decantation, washed with cold pentane (-40 °C, 3x 1 mL) and dried in vacuo (yield: 57 mg, 0.066 mmol, 66% based on Am ligand).

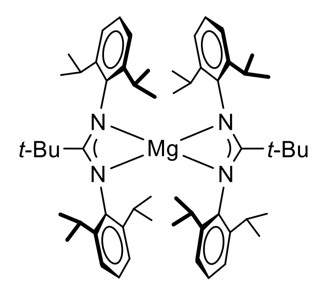

^{1}H NMR (400.13 MHz, C_6D_6, 298K): *δ* = 7.10 – 7.00 (m, 8H, Ar**H**), 6.96 – 6.89 (m, 4H, Ar**H**), 3.64 (p, ^3J = 6.8 Hz, 4H, C**H**$^{i\text{-}Pr}$), 3.22 (p, ^3J = 6.8 Hz, 4H, C**H**$^{i\text{-}Pr}$), 1.40 (d, ^3J = 6.8 Hz, 12H,C**H$_3$**$^{i\text{-}Pr}$), 1.38 (d, ^3J = 6.8 Hz, 12H,C**H$_3$**$^{i\text{-}Pr}$), 1.21 (d, ^3J = 6.8 Hz, 12H, C**H$_3$**$^{i\text{-}Pr}$), 0.90 (s, 18H, C**H$_3$**$^{t\text{-}Bu}$), 0.40 (d, ^3J = 6.8 Hz, 12H, C**H$_3$**$^{i\text{-}Pr}$) ppm.

^{13}C NMR (100.62 MHz, C_6D_6, 298K): δ = 180.7 ((*t*-Bu)**C**), 145.0 (Ar**C**-N), 143.6 (Ar**C**-C), 142.3 (Ar**C**-C), 124.5 (Ar**C**H), 123.6 (Ar**C**H), 123.5 (Ar**C**H), 44.0 (**C**(CH_3)$_3$), 31.0 C(**C**H_3)$_3$), 28.9 (**C**H(CH_3)$_2$), 28.8 (CH(**C**H_3)$_2$), 25.8 (CH(**C**H_3)$_2$), 23.6 (CH(**C**H_3)$_2$), 23.2 (CH(**C**H_3)$_2$), 23.1 (CH(**C**H_3)$_2$) ppm.

Elemental analysis: Calculated for $C_{63}H_{98}N_4Mg$ (M = 935.81 g/mol, calculated with one co-crystallized molecule of pentane): C 80.86; H 10.56; N 5.99 %. Found: C 80.96; H 10.62; N 6.09 %.

1.3 NMR spectroscopy characterization

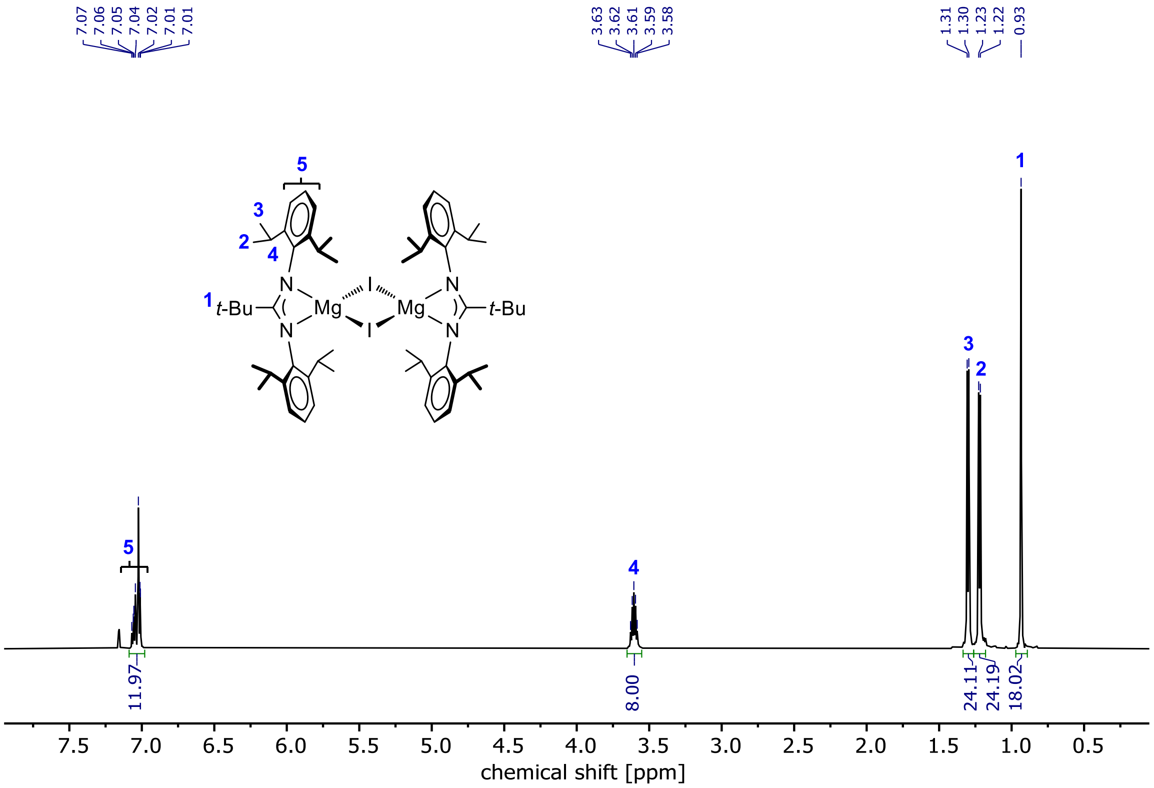

Figure S1. ^{1}H NMR (600.13 MHz, 298 K, C_6D_6) of [(Am)MgI]$_2$.

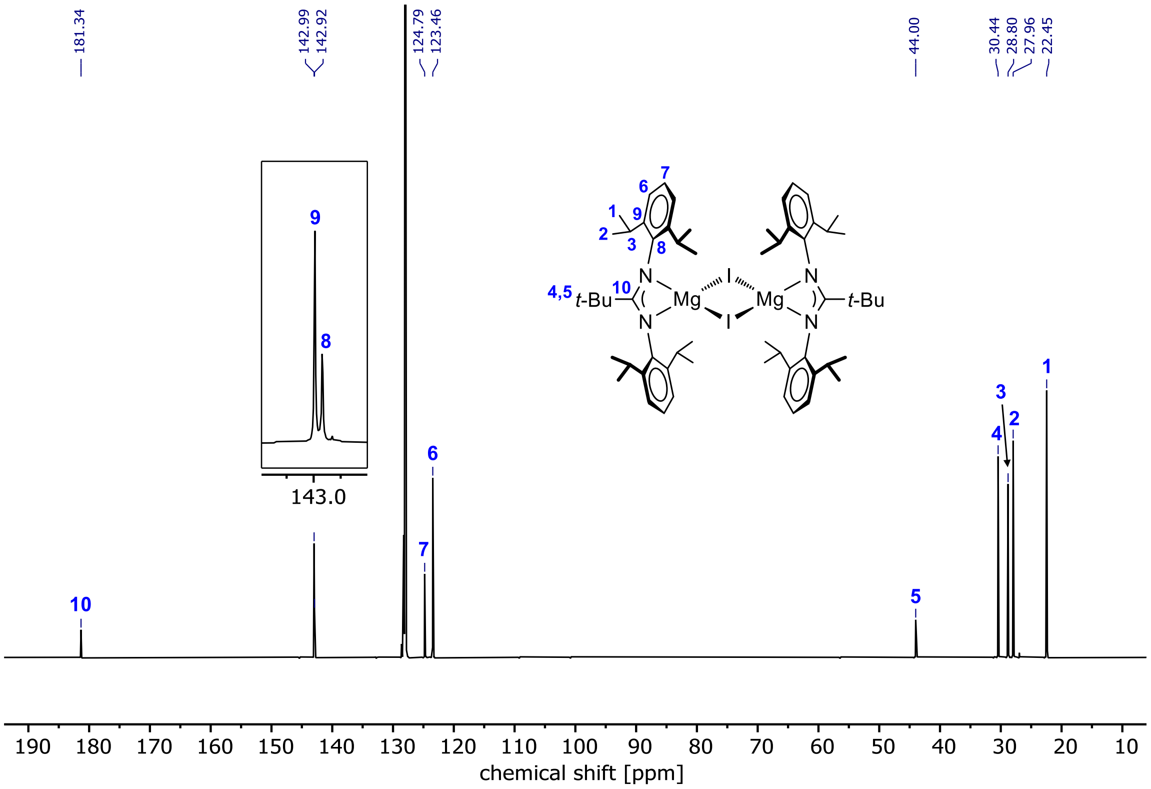

Figure S2. ^{13}C NMR (150.92 MHz, 298 K, C_6D_6) of [(Am)MgI]$_2$.

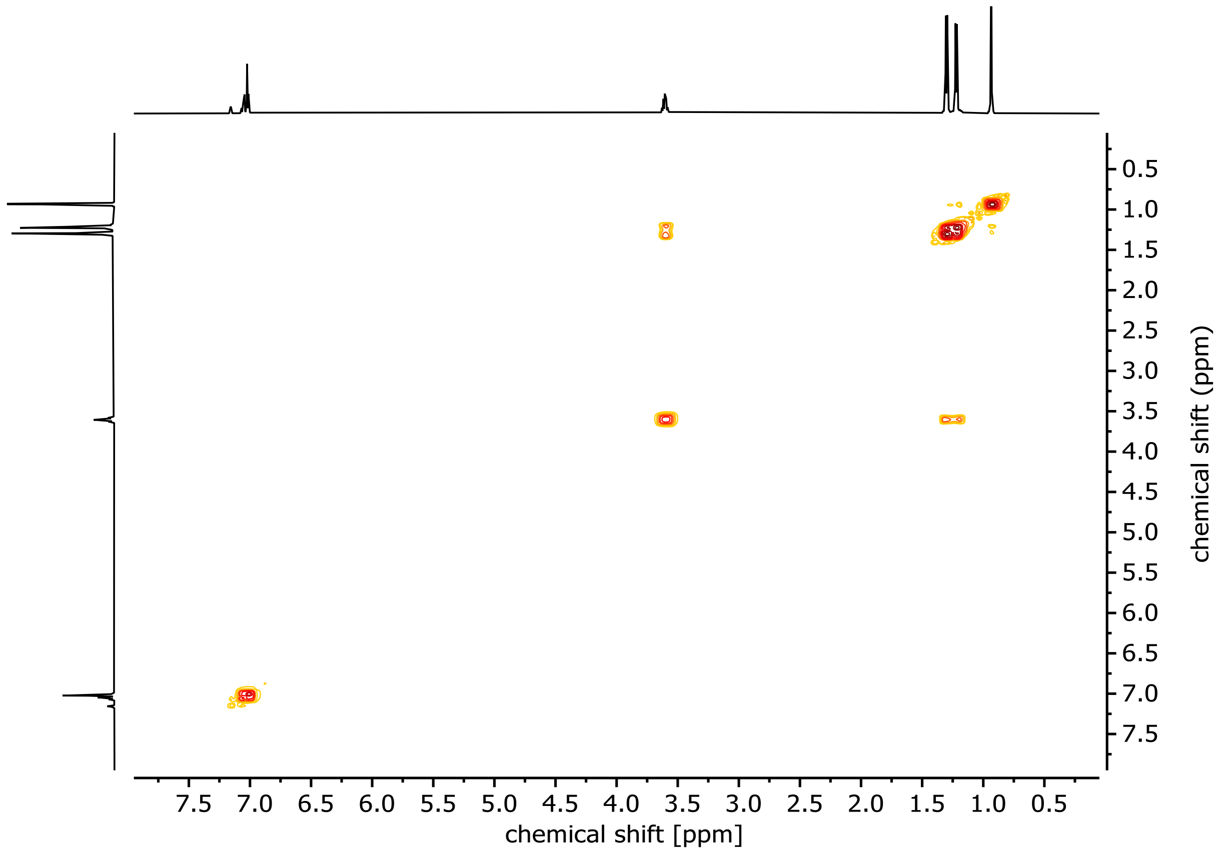

Figure S3. ^{1}H-^{1}H COSY NMR (600.13 MHz, 298 K, C_6D_6) of $[(Am)MgI]_2$.

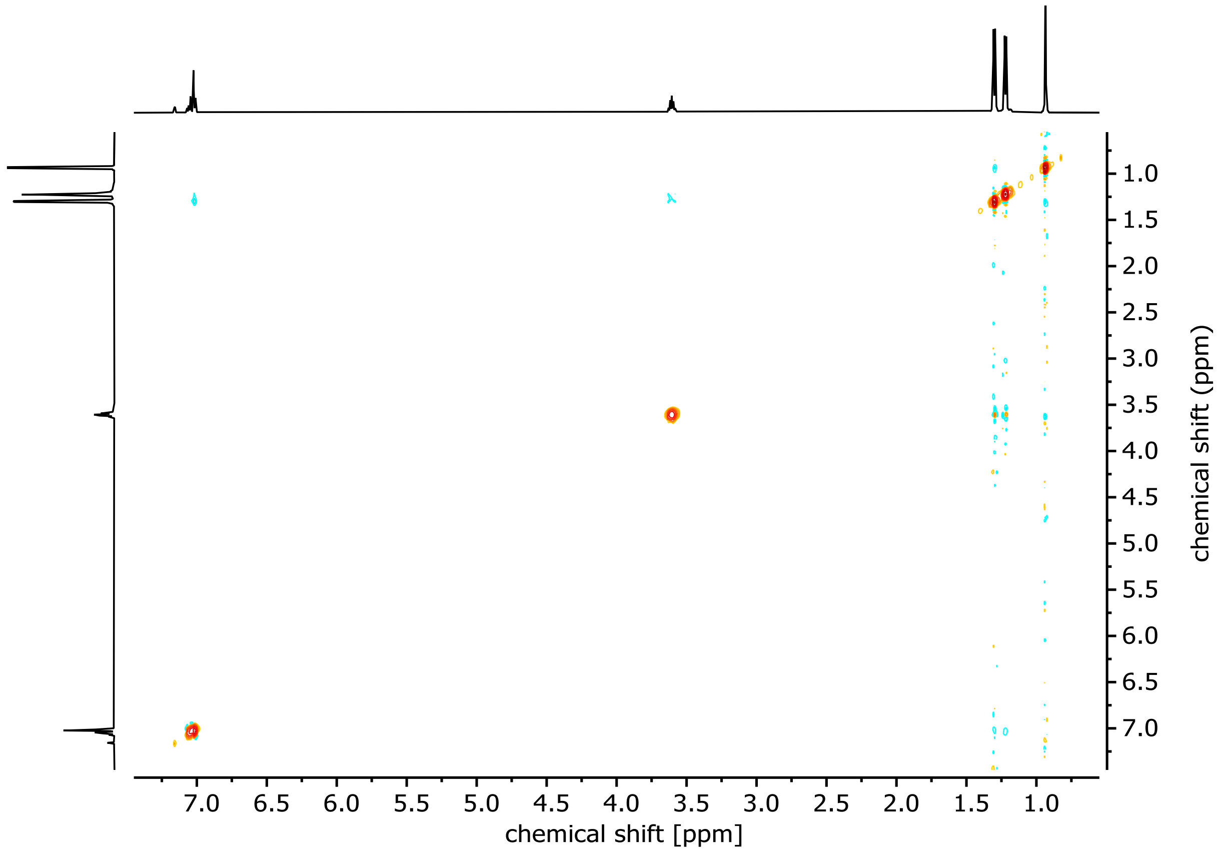

Figure S4. ^{1}H-^{1}H NOESY (600.13 MHz, 298 K, C_6D_6) of $[(Am)MgI]_2$.

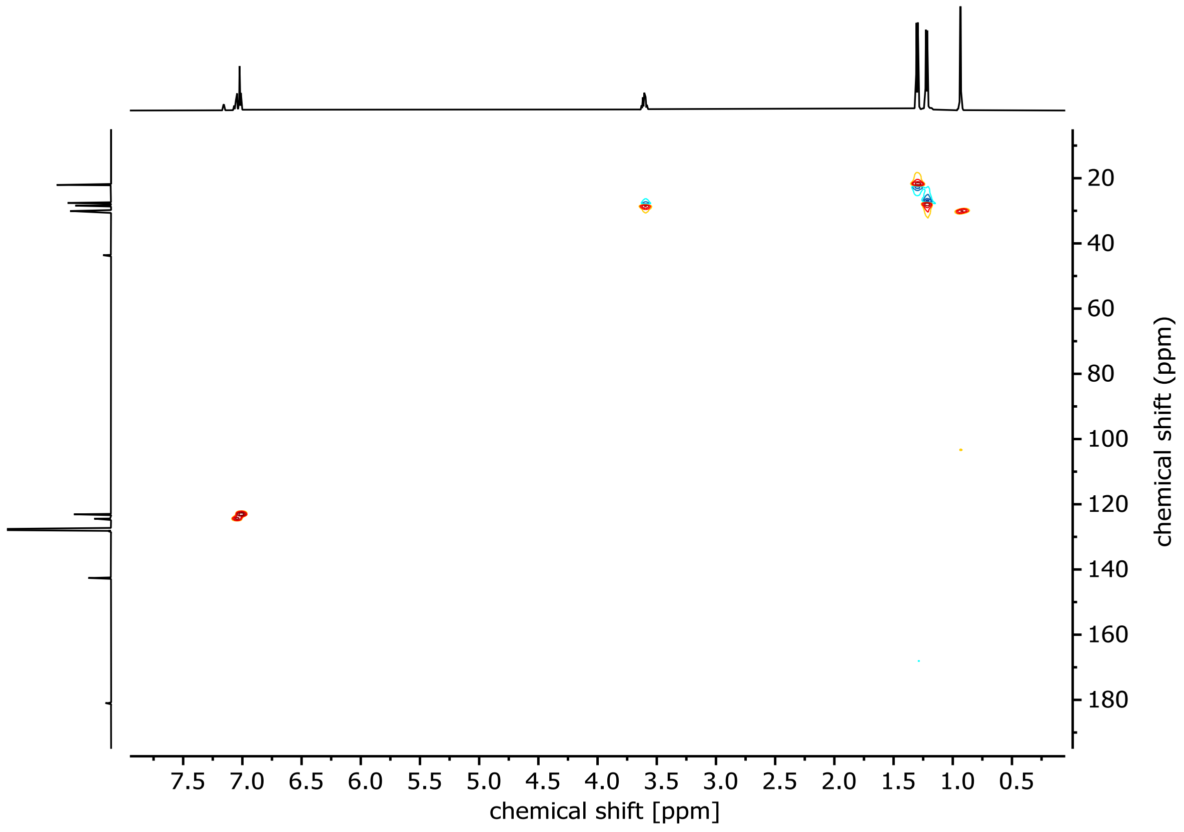

Figure S5. ^{1}H-^{13}C HSQC NMR (600.13/150.92 MHz, 298 K, C_6D_6) of $[(Am)MgI]_2$.

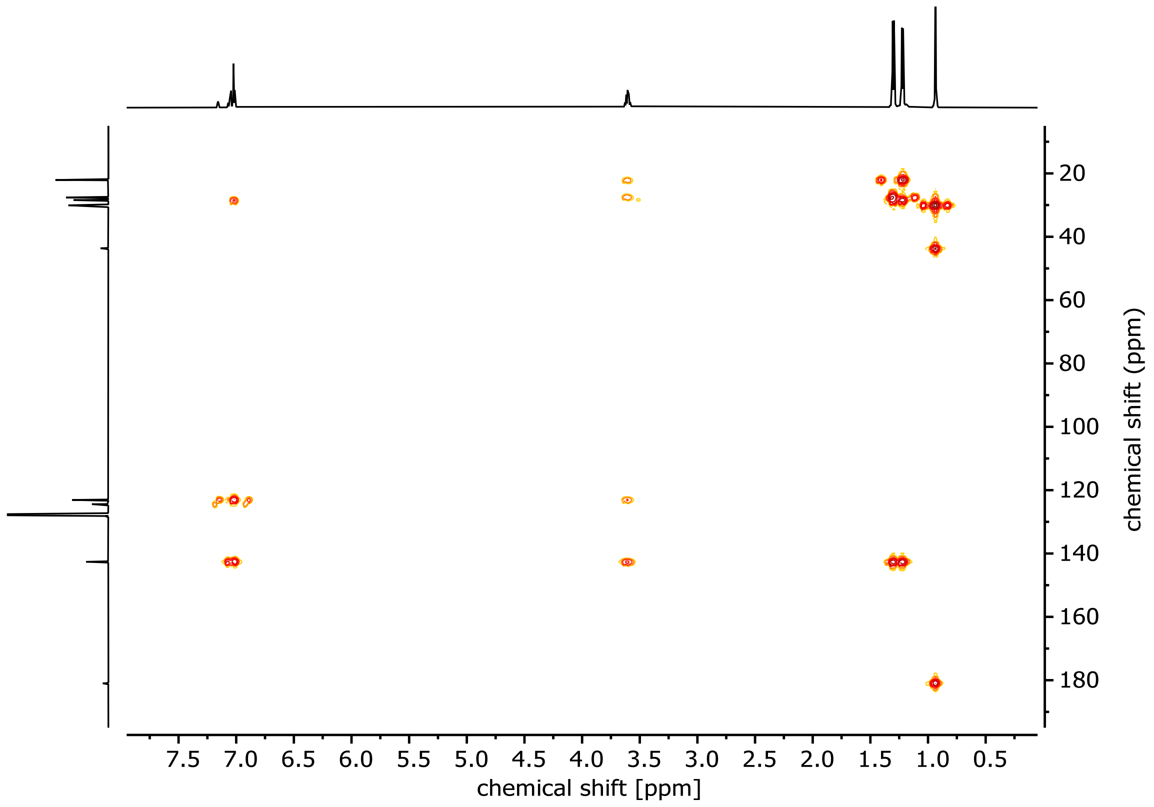

Figure S6. ^{1}H-^{13}C HMBC NMR (600.13/150.92 MHz, 298 K, C_6D_6) of $[(Am)MgI]_2$.

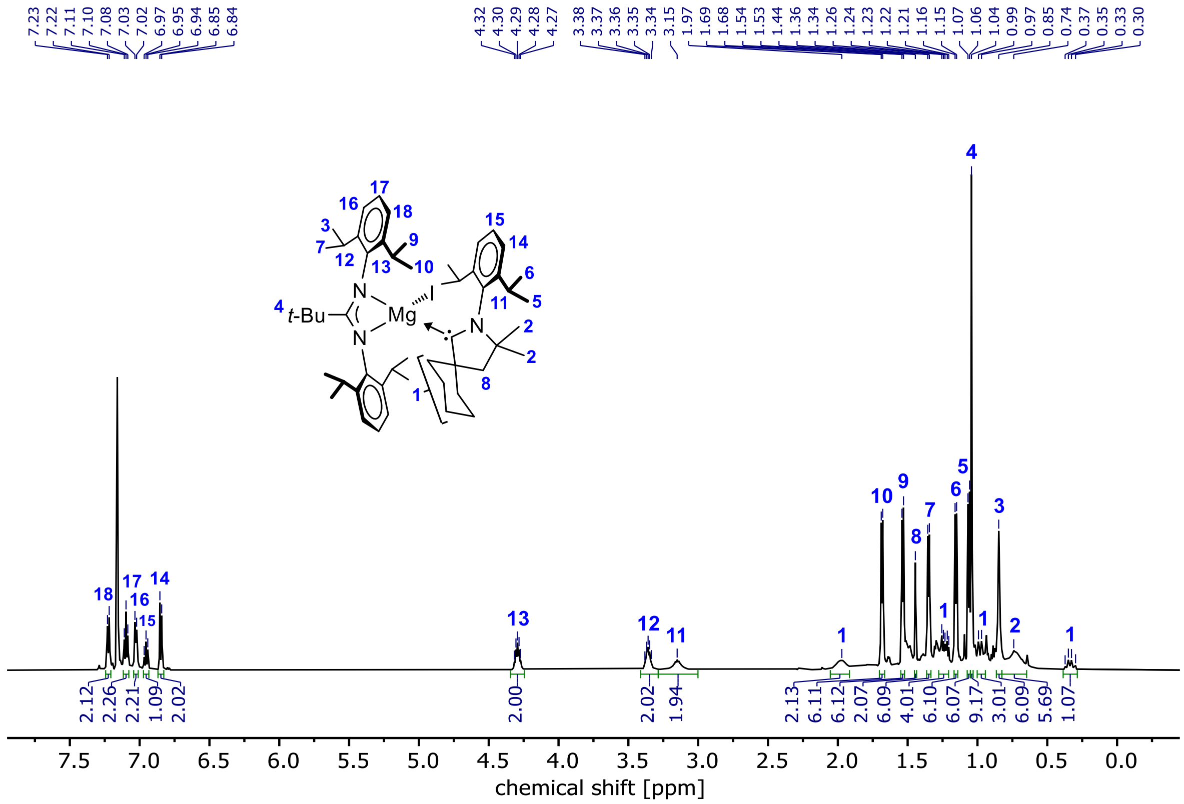

Figure S7. ^{1}H NMR (600.13 MHz, 298 K, C_6D_6) of (Am)MgI·(CAAC) (**1**).

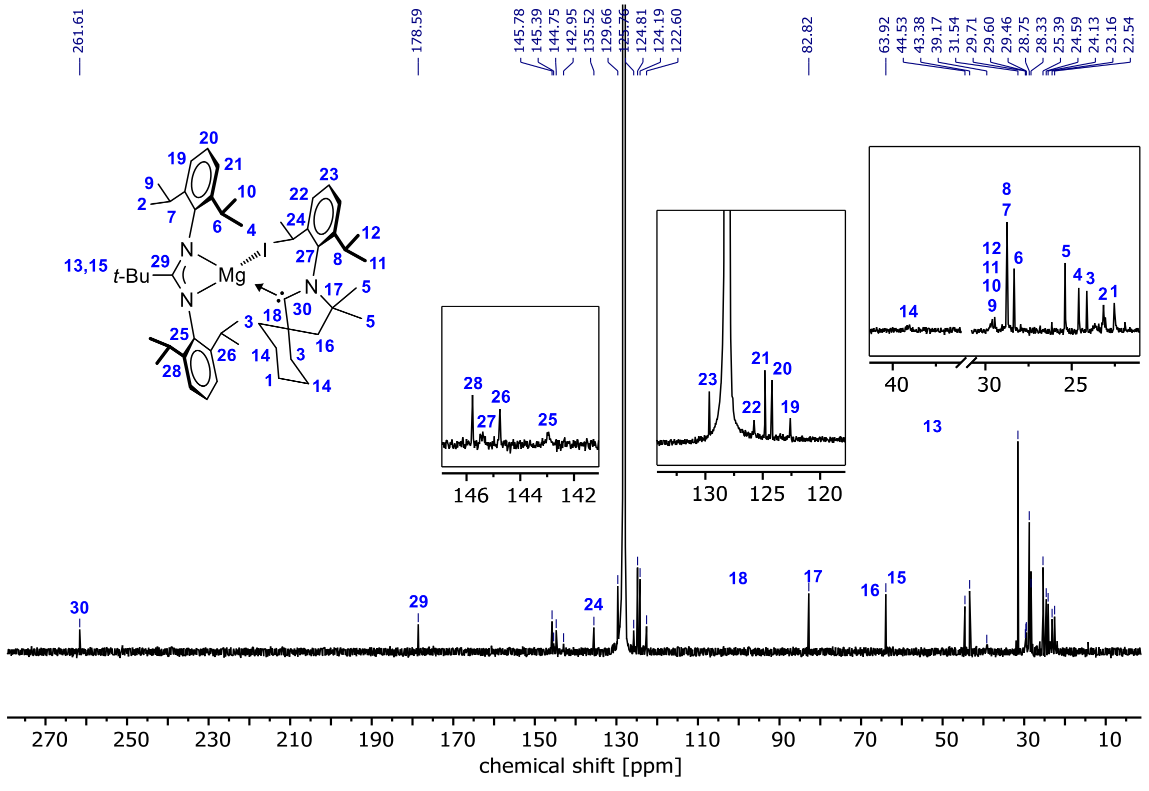

Figure S8. ^{13}C NMR (150.92 MHz, 298 K, C_6D_6) of (Am)MgI·(CAAC) (**1**).

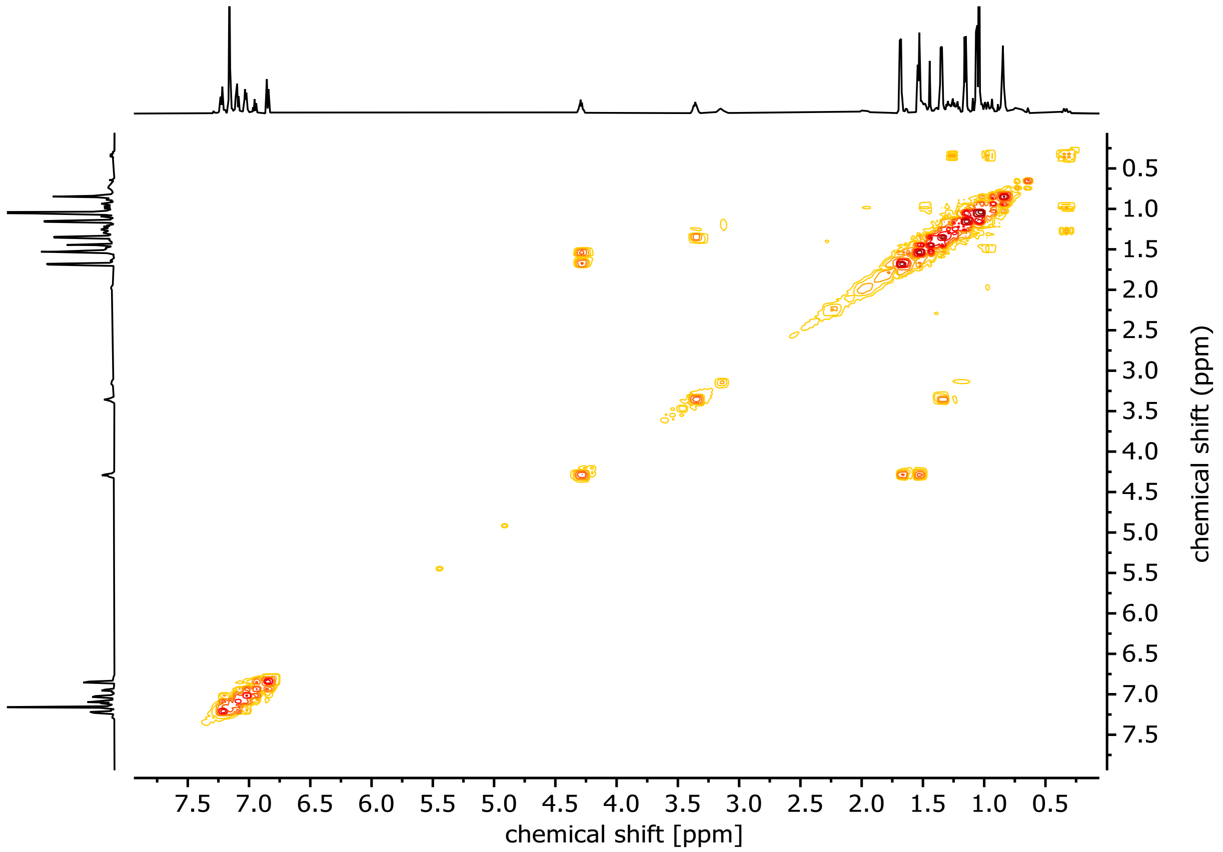

Figure S9. ^1H-^1H COSY NMR (600.13 MHz, 298 K, C_6D_6) of (Am)MgI·(CAAC) (**1**).

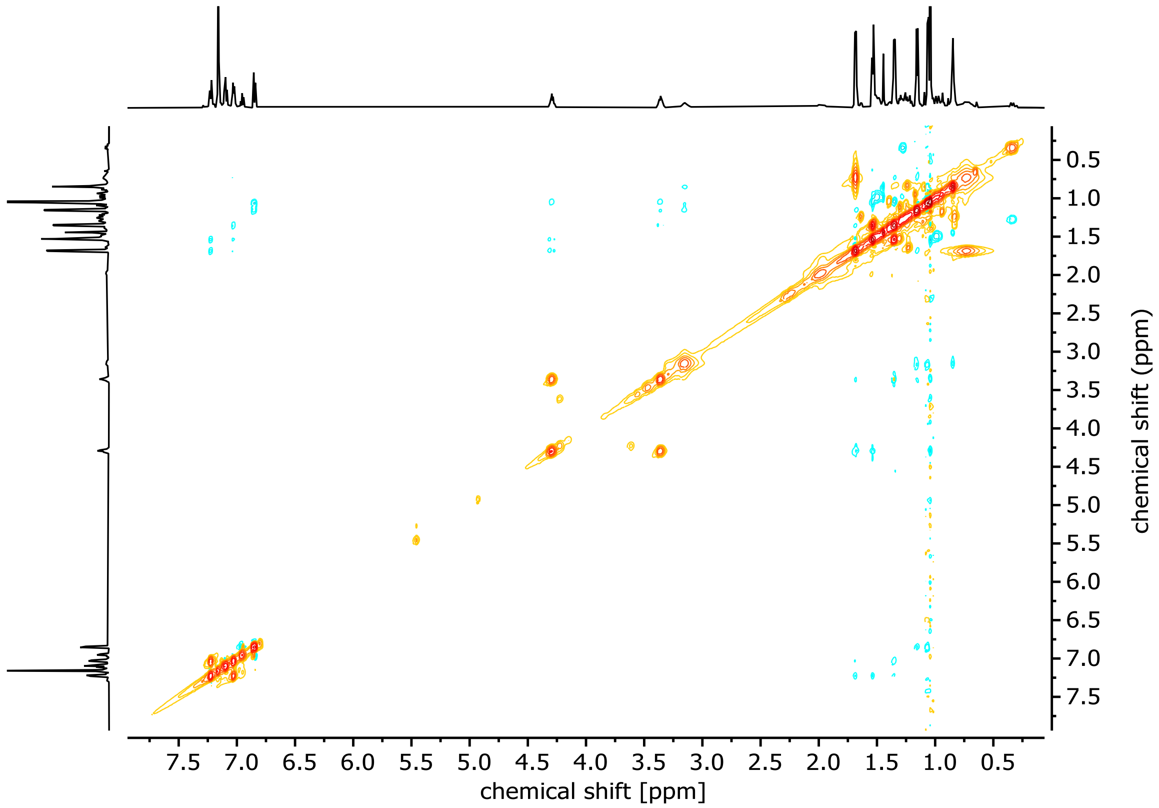

Figure S10. ^1H-^1H NOESY (600.13 MHz, 298 K, C_6D_6) of (Am)MgI·(CAAC) (**1**).

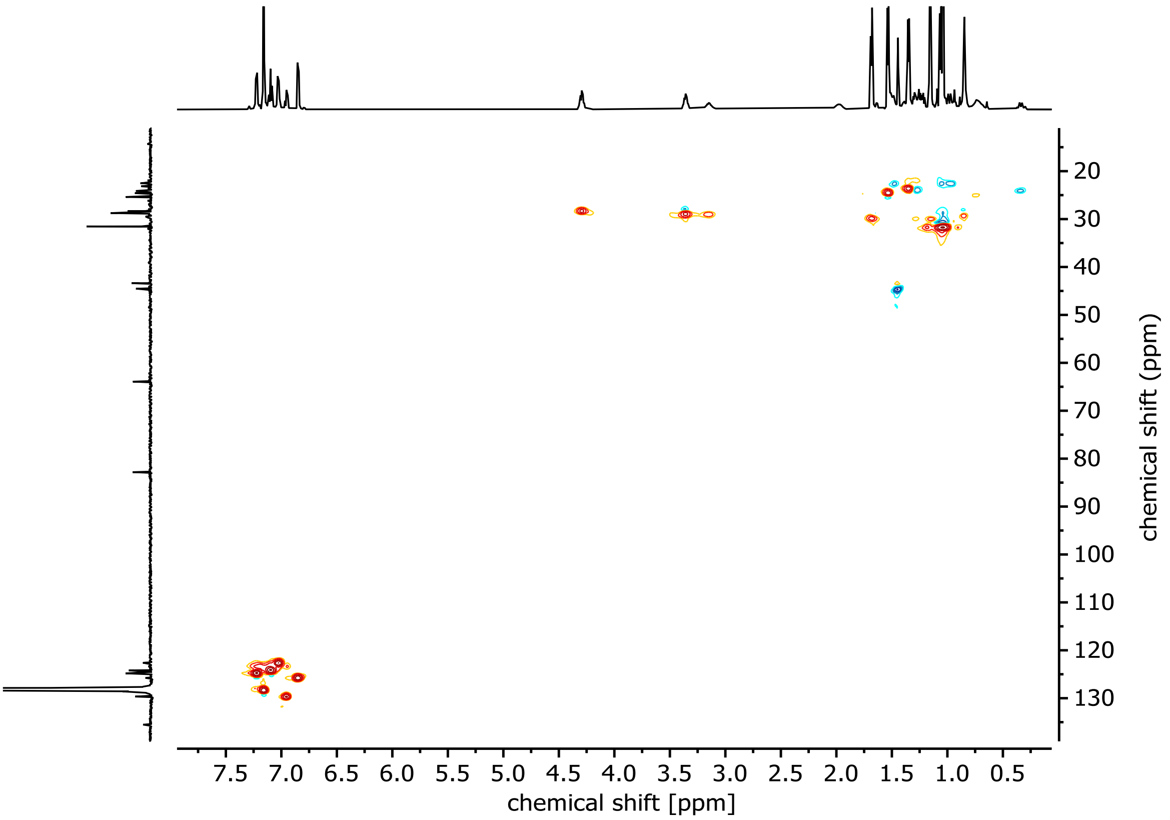

Figure S11. ^{1}H-^{13}C HSQC NMR (600.13/150.92 MHz, 298 K, C_6D_6) of (Am)MgI·(CAAC) (**1**).

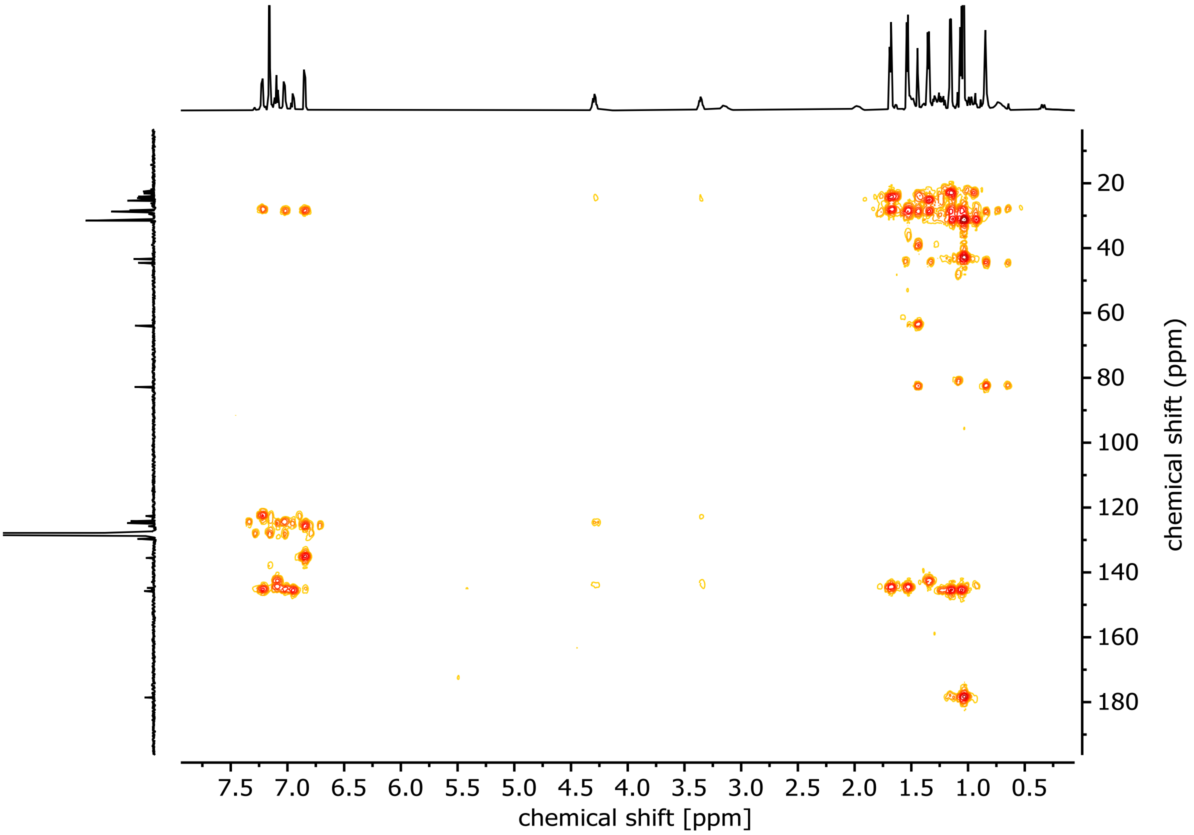

Figure S12. ^{1}H-^{13}C HMBC NMR (600.13/150.92 MHz, 298 K, C_6D_6) of (Am)MgI·(CAAC) (**1**).

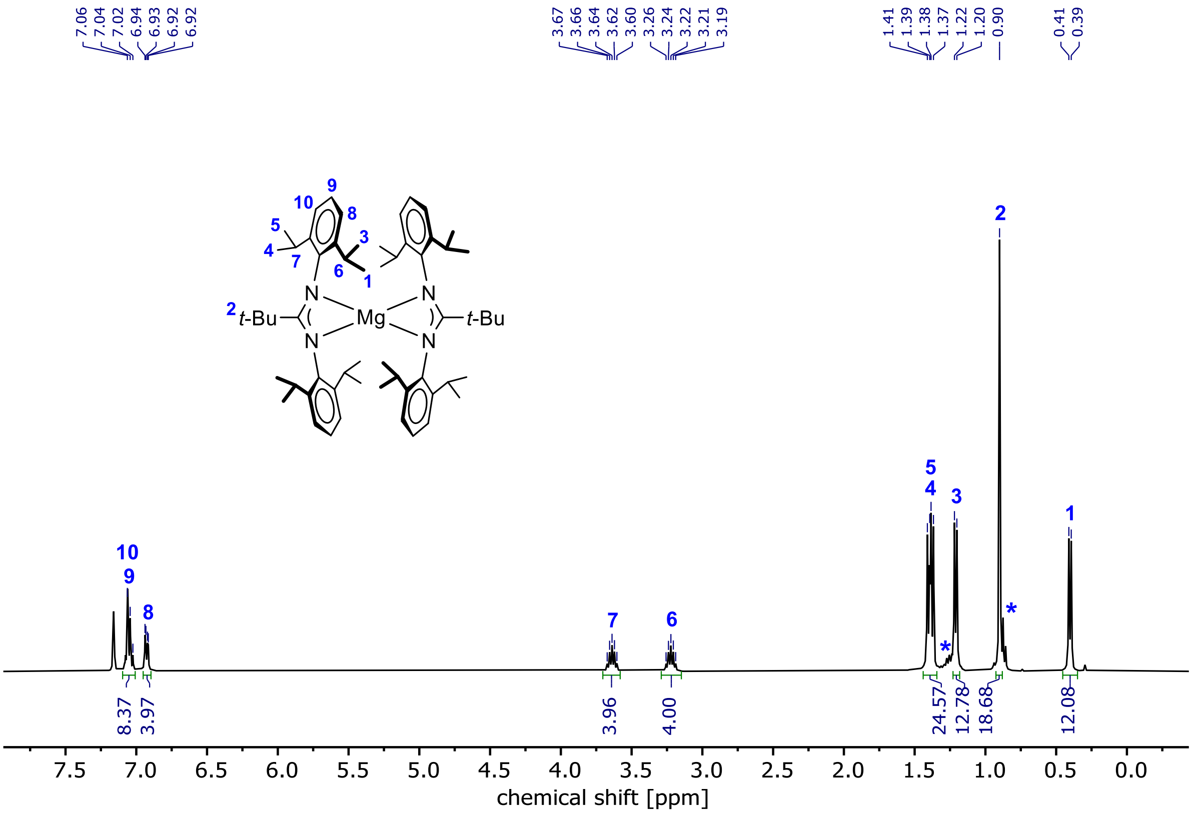

Figure S13. ^{1}H NMR (400.13 MHz, 298 K, C_6D_6) of $(Am)_2Mg$ (**3**). *denotes traces of pentane.

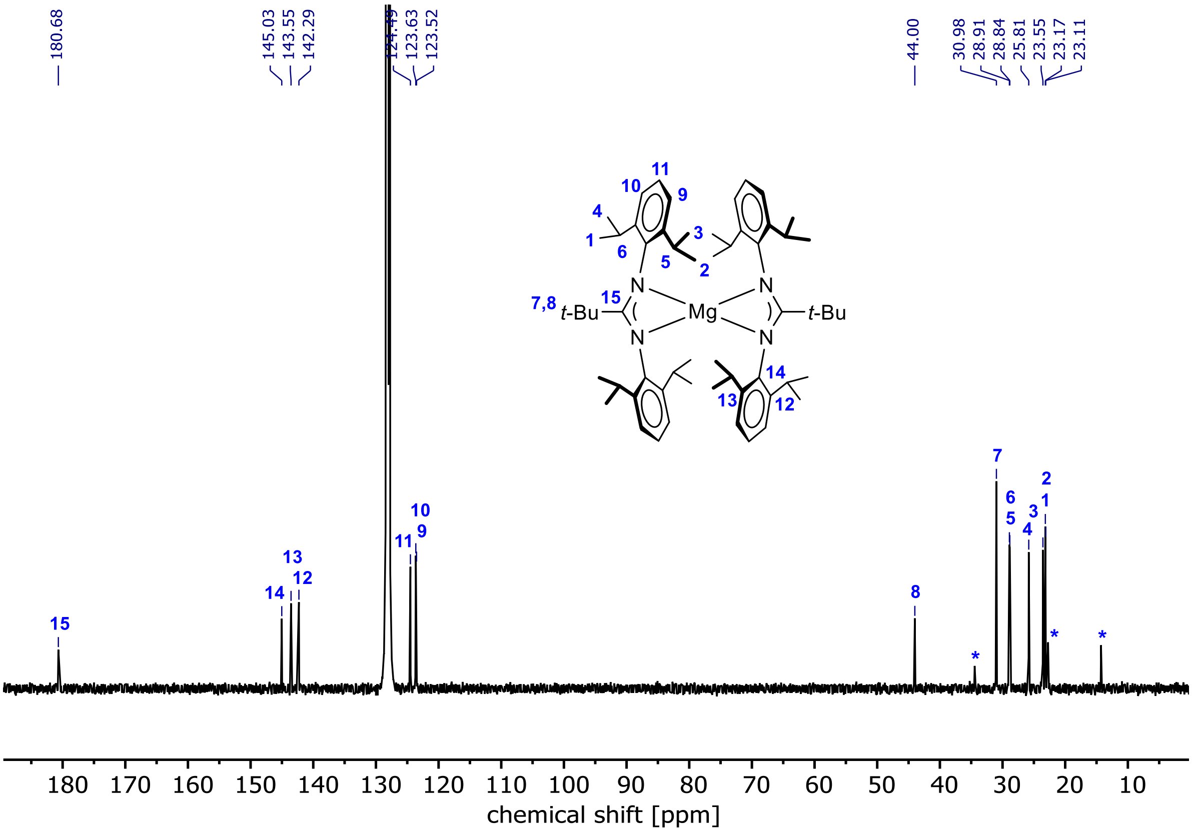

Figure S14. ^{13}C NMR (100.62 MHz, 298 K, C_6D_6) of $(Am)_2Mg$ (**3**). * denotes co-crystallized pentane.

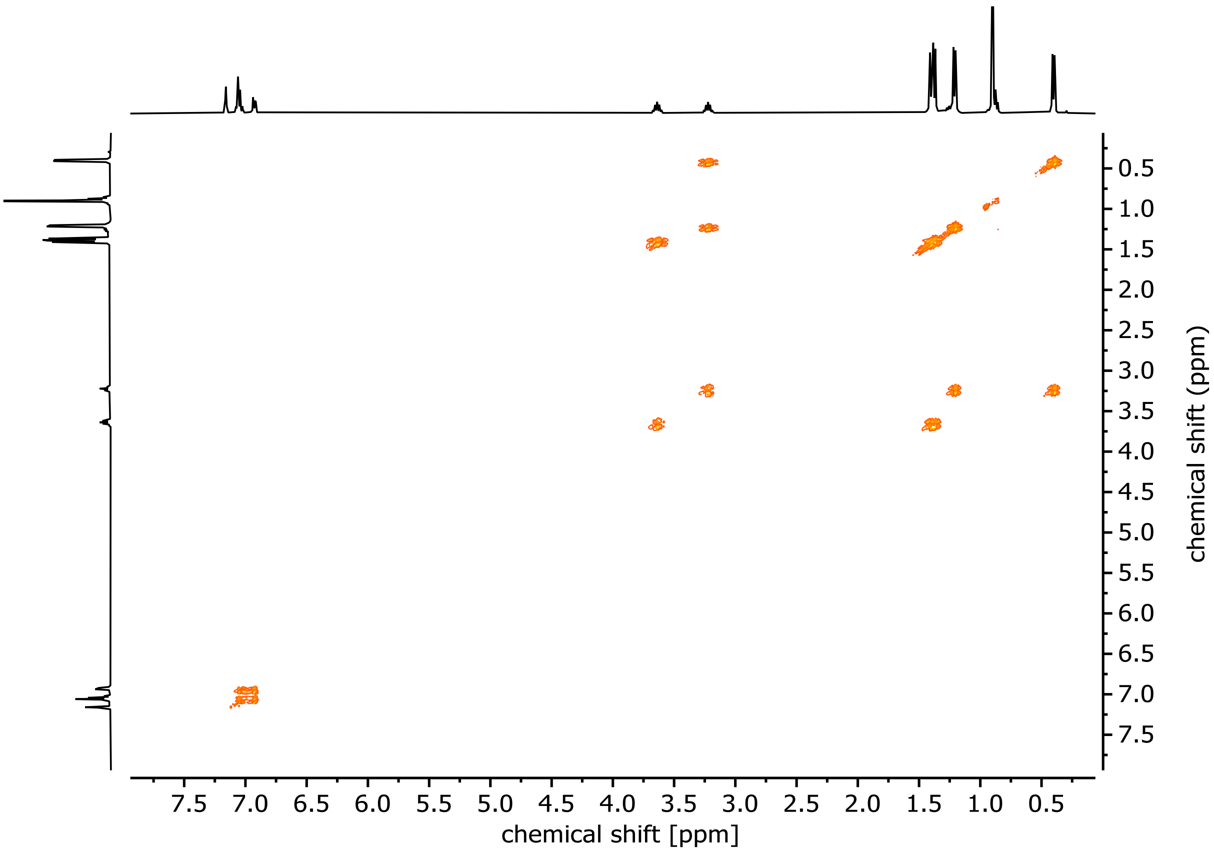

Figure S15. ^{1}H-^{1}H COSY NMR (400.13 MHz, 298 K, C_6D_6) of $(Am)_2Mg$ (**3**).

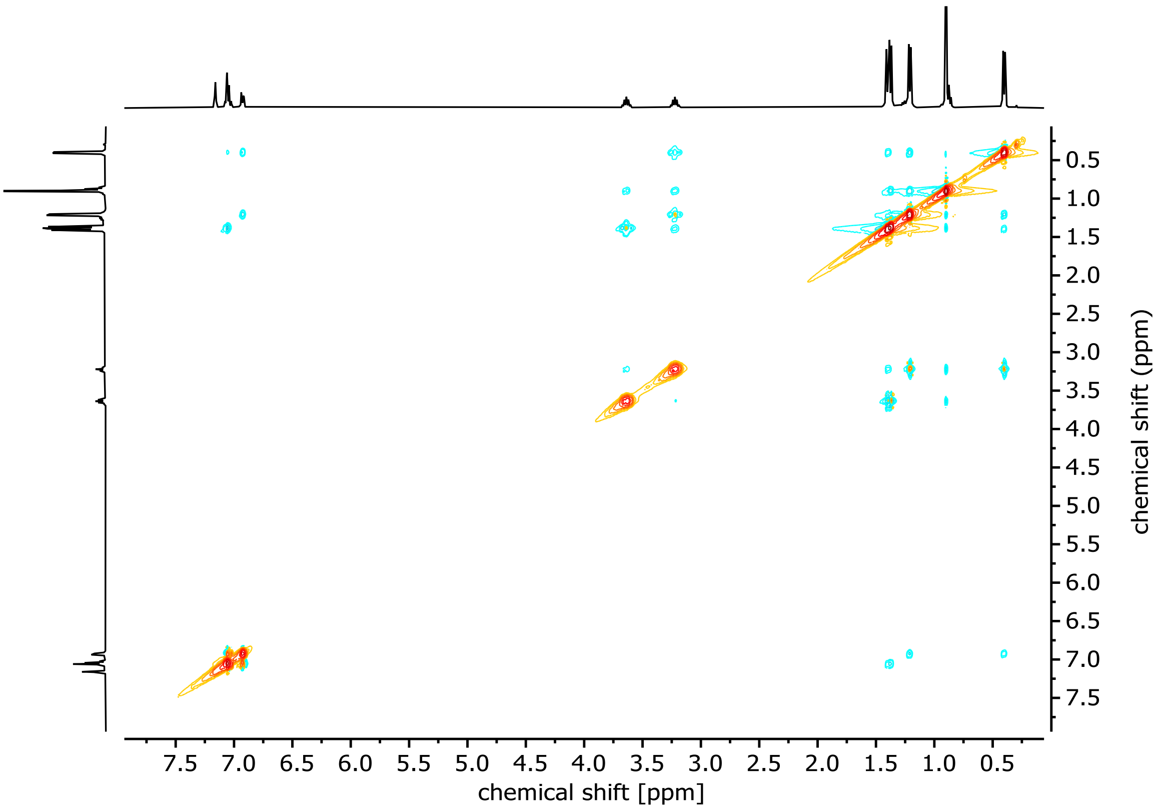

Figure S16. ^{1}H-^{1}H NOESY (400.13 MHz, 298 K, C_6D_6) of $(Am)_2Mg$ (**3**).

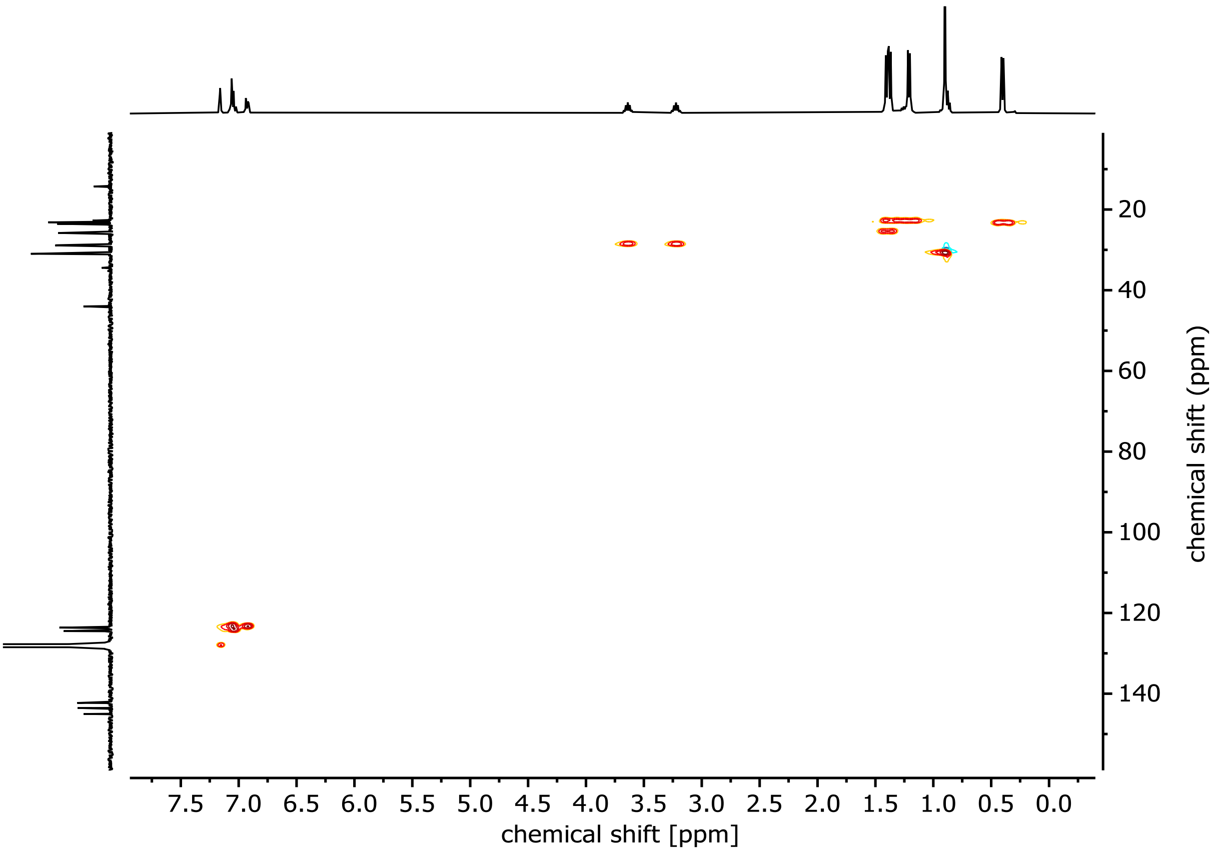

Figure S17. ^{1}H-^{13}C HSQC NMR (400.13/100.62 MHz, 298 K, C_6D_6) of $(Am)_2Mg$ (**3**).

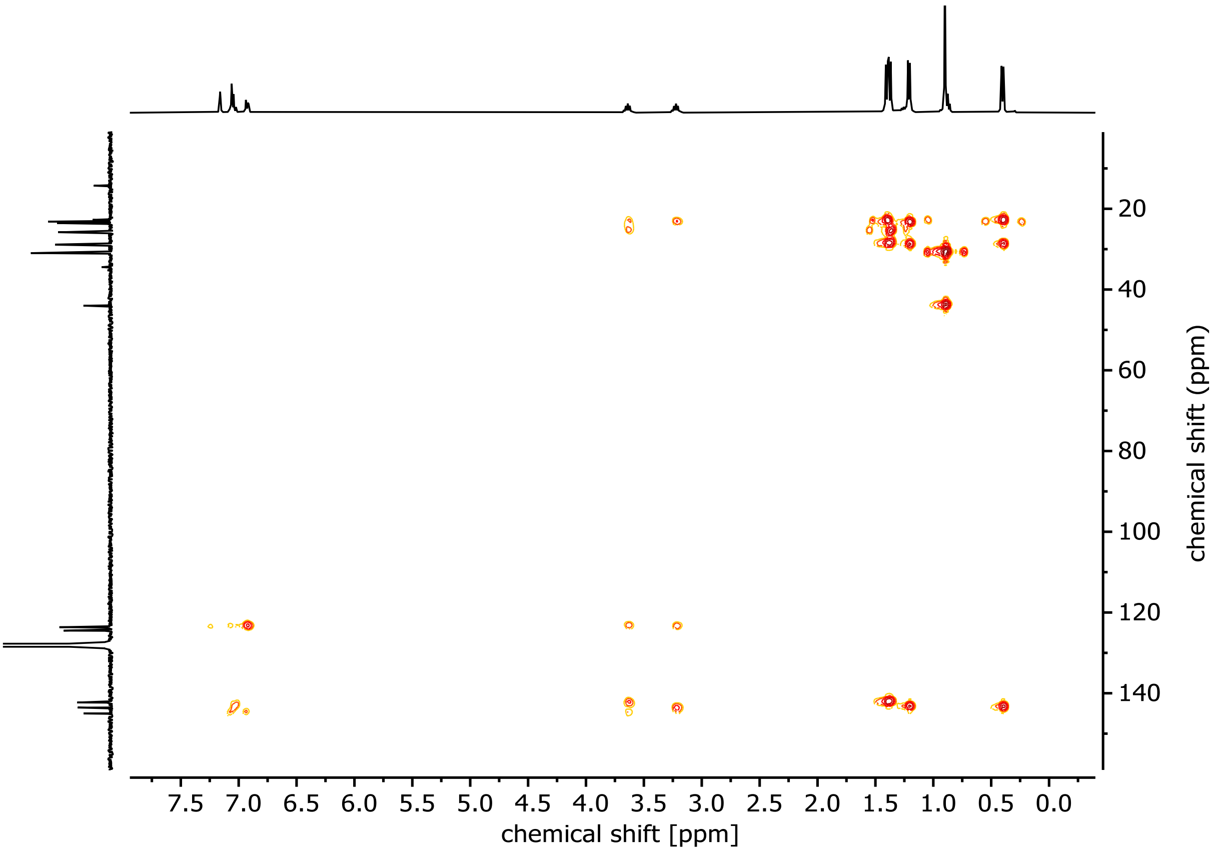

Figure S18. ^{1}H-^{13}C HMBC NMR (400.13/100.62 MHz, 298 K, C_6D_6) of $(Am)_2Mg$ (**3**).

1.4 Stability and NMR spectra of (Am)Mg·(CAAC) (2)

The stability of (Am)Mg•·(CAAC) (**2**) was tested with ^{1}H NMR. Complex **2** dissolves very well in C_6D_6 and a fresh solution was immediately transferred in a NMR machine. The ^{1}H NMR spectra were followed over time. The initial broad signals of the paramagnetic product are replaced with sharp signals of the diamagnetic decomposition products. The homoleptic complex $(Am)_2Mg$ (**3**) is a major decomposition product.

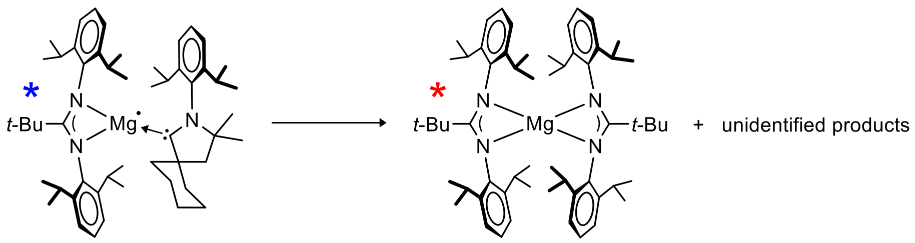

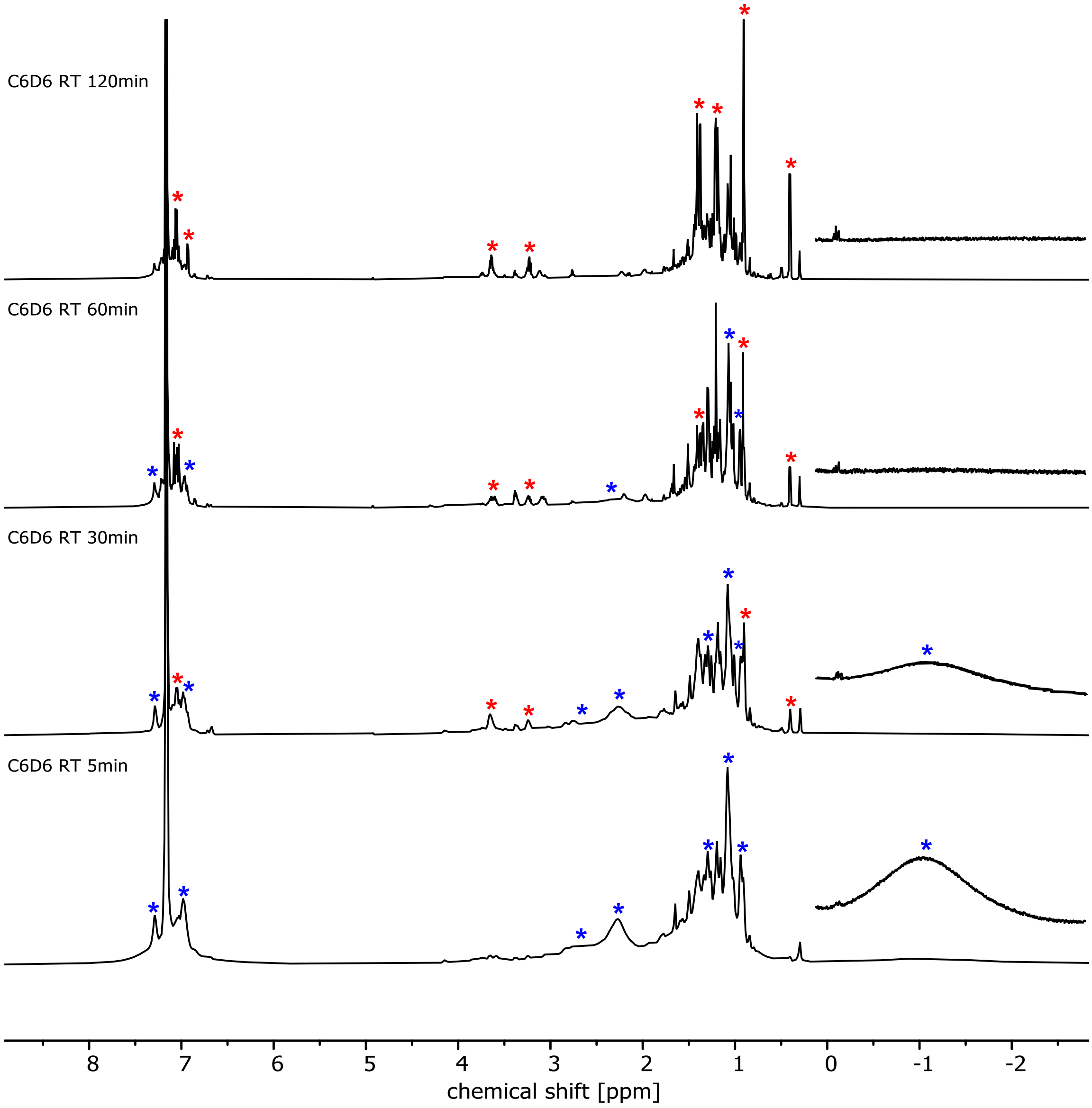

Figure S19. ^{1}H NMR spectra (600.13 MHz, 298 K, C_6D_6) for decomposition of (Am)Mg·(CAAC) (blue *) to $(Am)_2Mg$ (red *) and other unidentified products.

1.5 EPR, IR and UV spectra of (Am)Mg·(CAAC) (2)

EPR samples were prepared in a N_2 glovebox with <0.1 ppm residual O_2 and H_2O. Samples were prepared in 4-mm quartz EPR tubes brazed onto Young-style valves, then were immediately removed from the glovebox, frozen in liquid N_2 and flame-sealed. Samples were stored in liquid N_2 until measurement.

EPR spectra were simulated with an effective spin Hamiltonian using the package EasySpin within MATLAB.[S4] Hyperfine coupling constants were calculated using the ORCA 5.0.1 electronic structure package,[S5,S6] using an unrestricted KS determinant, the B3LYP functional,[S7,S8] the Def2-TZVP basis set,[S9] the Def2/J auxiliary basis set,[S10] CPCM(benzene) solvation,[S11] and default ORCA 5 parameters otherwise. For the EPR/NMR property calculation, the basis set was fully decontracted on the atoms of interest, a necessary procedure to correctly model electron density near the nucleus.[S12,S13] The calculated isotropic hyperfine couplings were not significantly sensitive to the density functional used, nor to the optimized geometry as compared to the crystallographic geometry (with only hydrogen positions optimized) (Table S1).

Table S1. Hyperfine coupling constants (mT), calculated with different functionals and geometries.

	**B3LYP**	**B3LYP H-Opt**	**PBE0**
^{25}Mg	0.25	0.23	0.25
^{14}N	0.37	0.37	0.36
^{13}C	1.46	1.42	1.44

As discussed in the main text, complex **2** was found to be unstable in every solvent in which it dissolved. Two EPR spectra of one sample in toluene were measured at ~300 K, after about 5 and 35 minutes spent in liquid solution. While the total (double-integrated) EPR intensity decreased by 90% in 30 minutes, the signal shape remained constant.

In addition to the isotropic solution spectra, EPR spectra of the frozen solution and the pure powder were measured (Figure S20). As expected, the linewidths are much worse than those observed in the liquid solution, with only ^{14}N hyperfine structure observable in the frozen solution. These spectra simply confirm that **2** is paramagnetic, and support the working hypothesis that the isotropic EPR spectrum is dominated by signals from the target complex.

Together, these data support our assumption that the EPR spectrum presented in the main text is in fact predominantly complex **2**. The agreement between the spin Hamiltonian simulation and the experimental EPR spectrum is good, but not perfect in the region of the satellites. Exploratory simulations with various additional hyperfine couplings or additional broad derivative signals were not illuminating. The simulation deviation may result from a tiny steady-state population of a transient paramagnetic decomposition product.

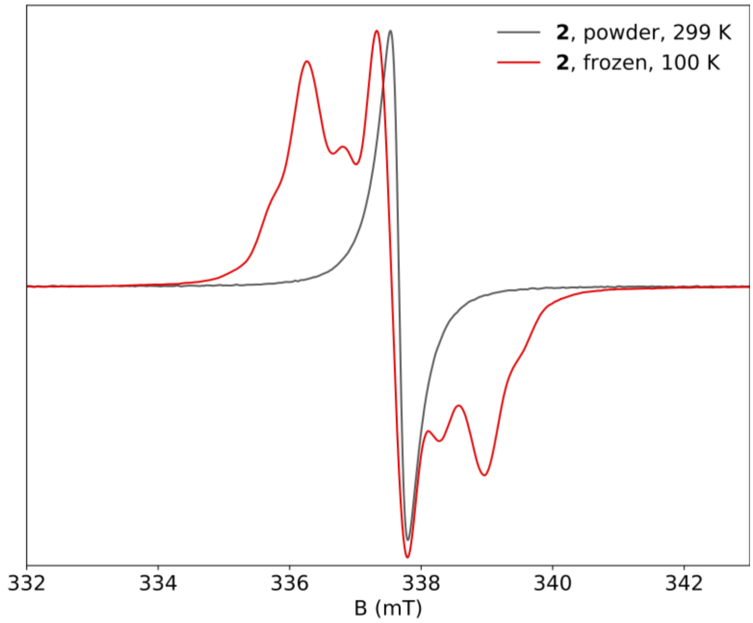

Figure S20. EPR spectra of **2** recorded as a powder (power 0.01 mW) and as a dilute frozen solution in toluene.

ATR-Infra-red spectra of solid (Am)Mg·(CAAC) (**2**) were measured of the pure compound in the inert N_2 atmosphere of a glovebox. The IR spectrum of **2** is shown in Figure S21.

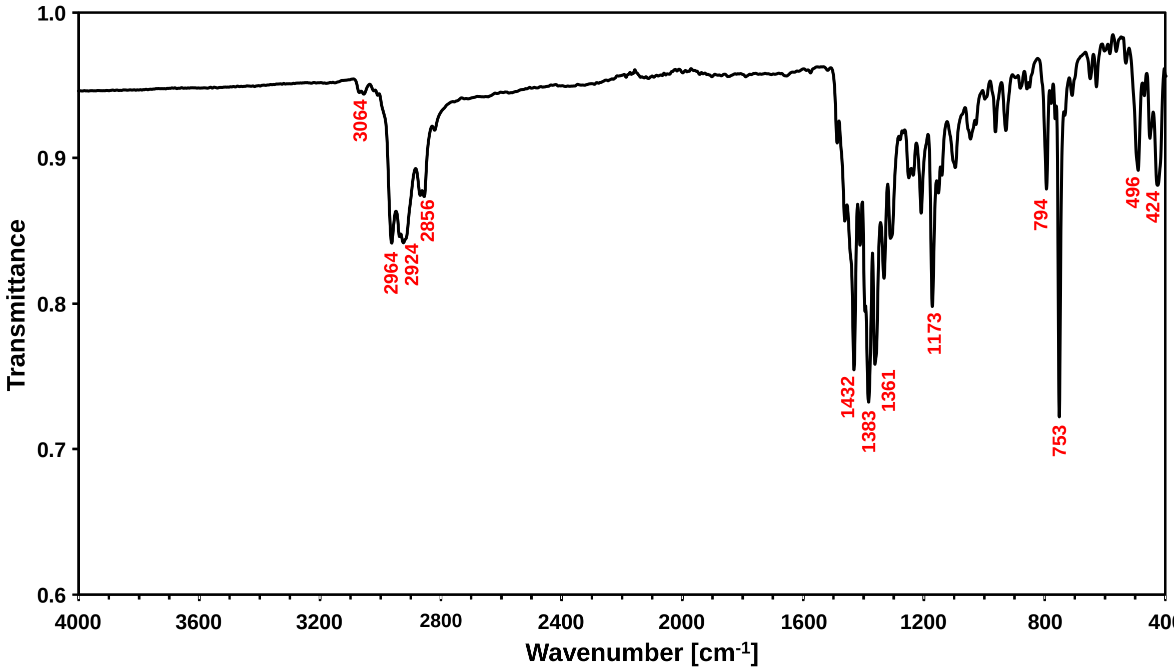

Figure S21. ATR-IR spectrum of **2** recorded as a neat powder. Selected bands at 3064, 2964, 2924, 2856, 1432, 1383, 1361, 1173, 794, 753, 496, 424 cm^{-1}.

UV-vis spectra of solid (Am)Mg·(CAAC) (**2**) were measured using the following method. In the inert N_2 atmosphere of a glovebox, a quartz cuvette was filled with benzene and frozen. Crystals of complex 2 were added and the cuvette was sealed. The purple-violet solution obtained after defrosting, was immediately measured on a UV-vis spectrometer and monitored over time (Figure S22).

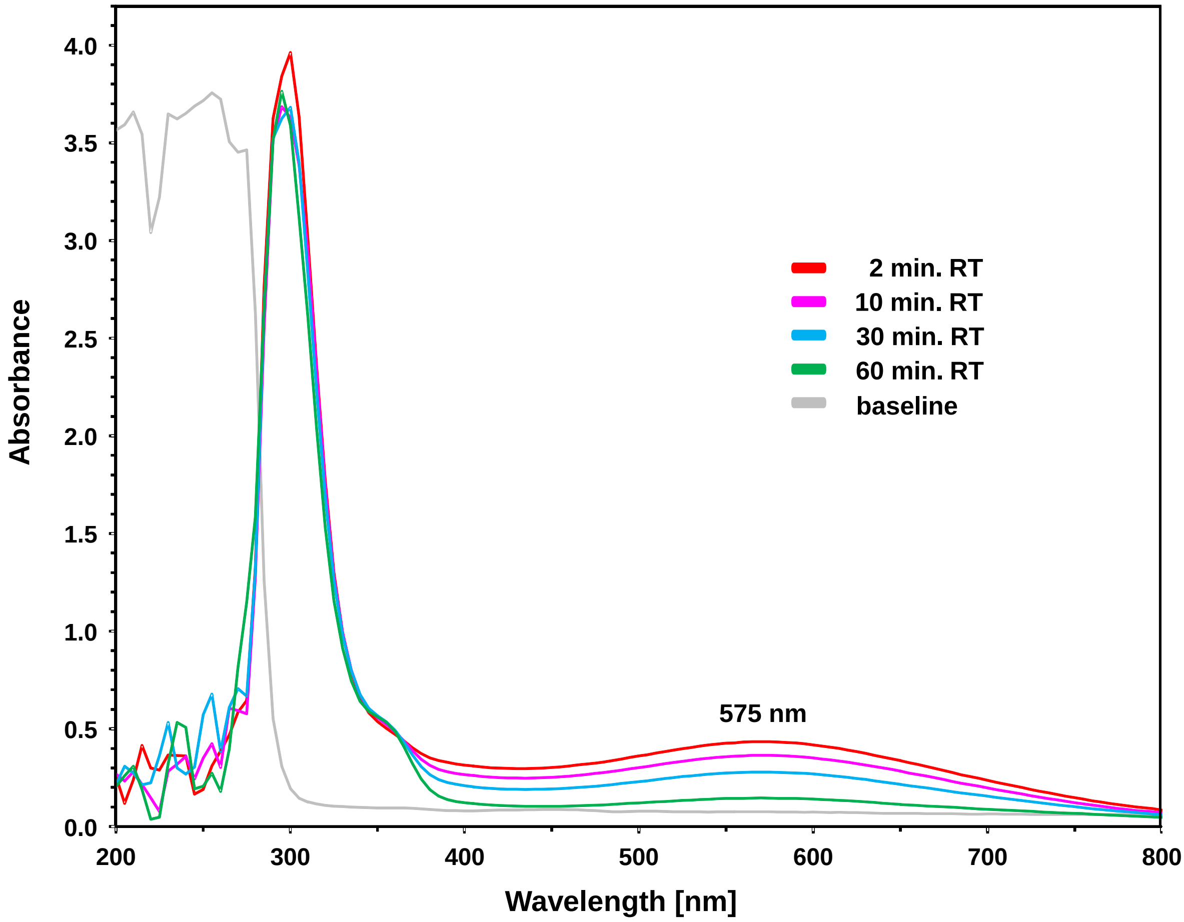

Figure S22. Superimposed UV-vis spectra following the decomposition of (Am)Mg·(CAAC) (**2**) in benzene (20 °C) over time. The characteristic absorption band for **2** is found at 575 nm.

1.6 Quenching (Am)Mg·(CAAC) (2) with iodine

Complex (Am)Mg·CAAC (**2**) (31 mg, 0.040 mmol) was added to a frozen benzene solution of I_2 (5 mg, 0.020 mmol) in 0.5 mL of C_6D_6. It was shaken vigorously until the benzene was completely defrosted. The color changed from deep purple to pale yellow. ^{1}H NMR analysis showed major conversion to (Am)MgI·CAAC) (**1**). Also $(Am)_2Mg$ and free CAAC is detected.

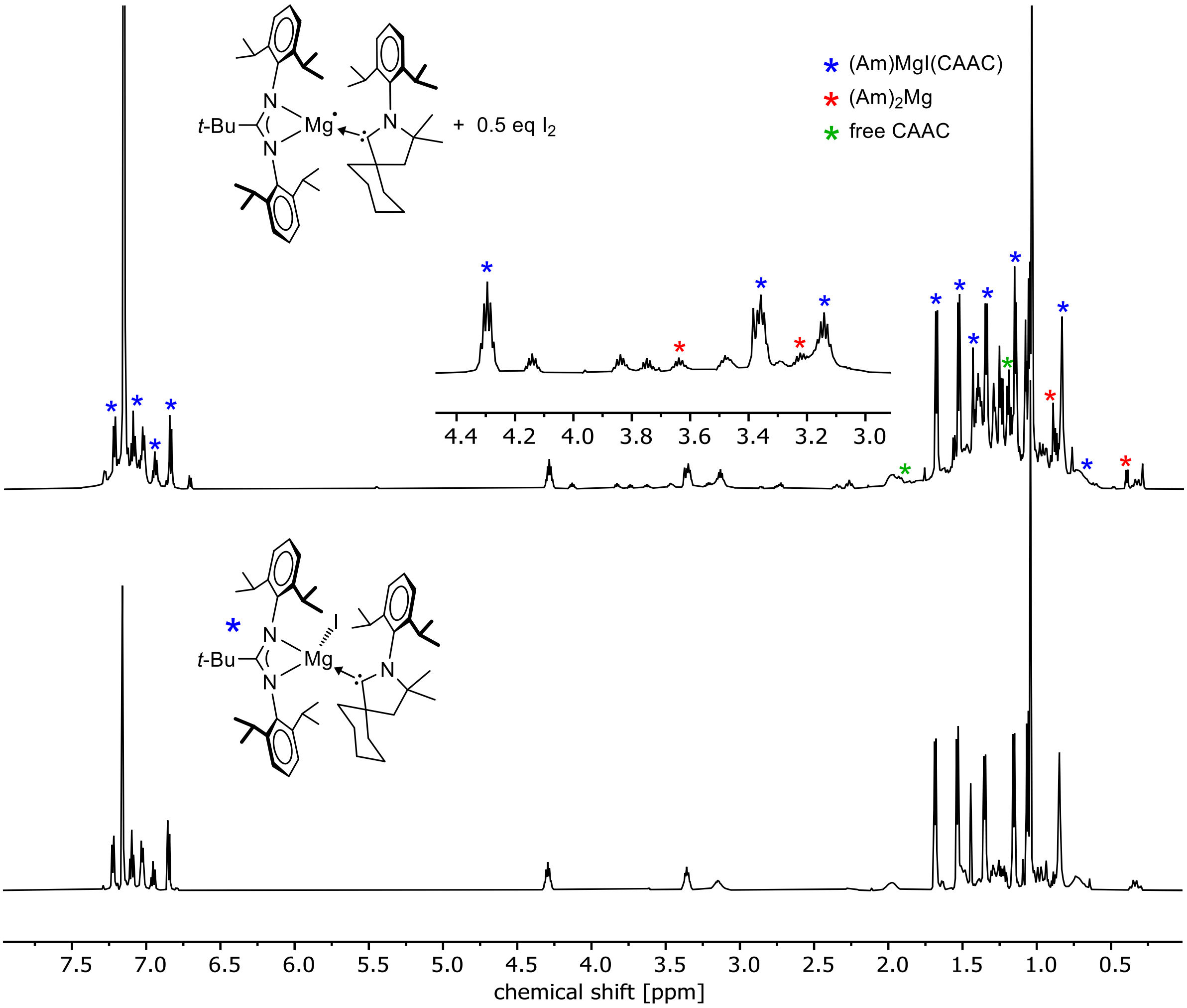

Figure S23. ^{1}H NMR spectra (600.13 MHz, 298 K, C_6D_6) for the product of reaction between (Am)Mg·(CAAC) (**2**) and 0.5 equivalent of I_2 (bottom) and (Am)MgI·(CAAC) (**1**) (top).

1.7 DFT calculations

All calculations were carried out using Gaussian 16A.[S14] All methods were used as implemented. All structures were fully optimized on a B3PW91/def2SVP level of theory.[S15] All structures were characterized as true minima (Nimag=0) by frequency calculations on the same level of theory. Energies were determined at a B3PW91/def2TZVP level of theory. In all cases Grimme`s third dispersion correction with Becke-Johnson damping (GD3BJ) was added, unless indicated otherwise.[S16] Charges were calculated via NBO7 Analyses.[S17] All structures were evaluated using Molecule 2.3.[S18] Topological analyses were carried out with AIMAll17 using the wave function of the B3PW91/def2TZVP calculation.[S19]

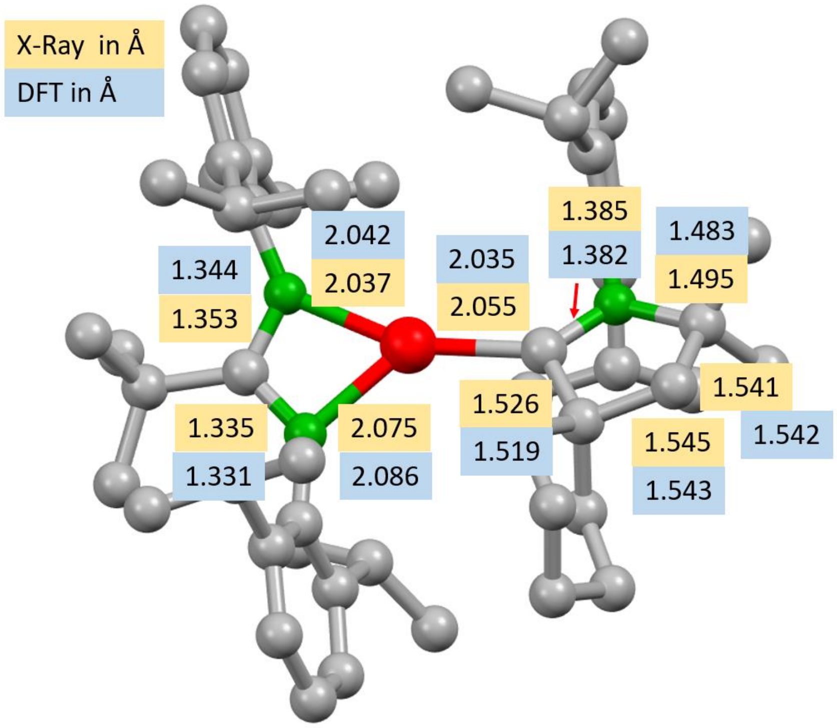

Figure S24. Comparison of bond distances in the crystal structure of (Am)Mg·(CAAC) (**2**) with those in the calculated structure.

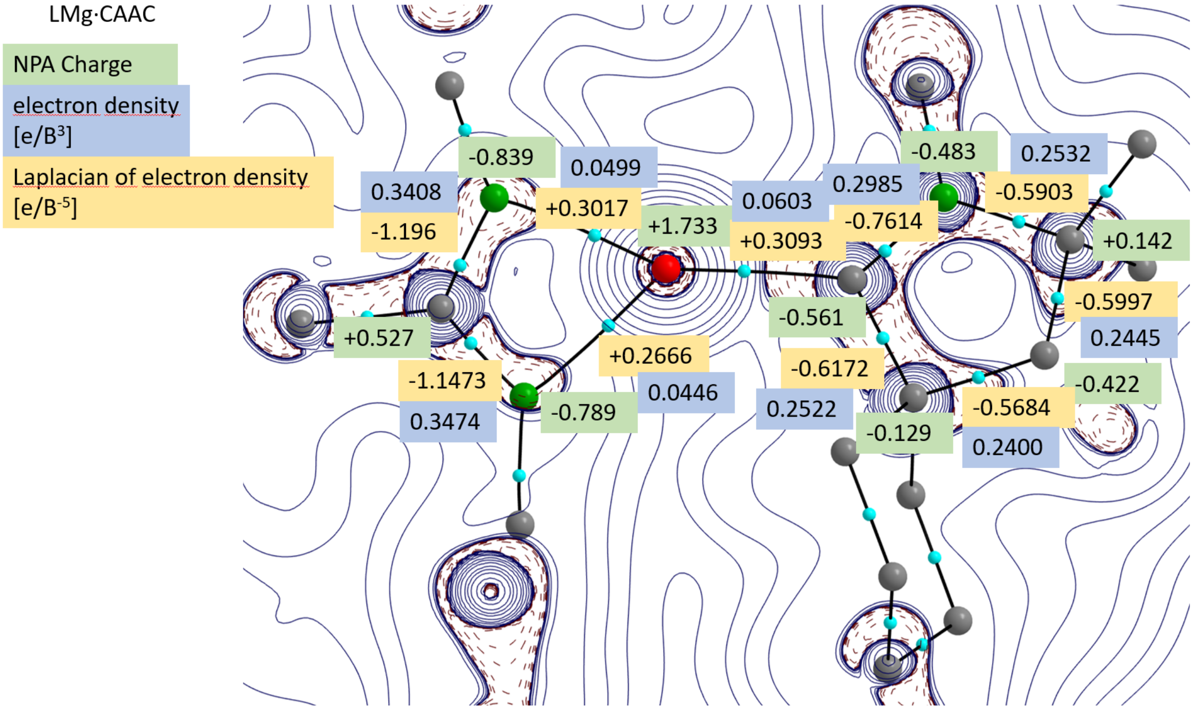

Figure S25. Contour plot of the Laplacian $\nabla^2\rho$ for complex (Am)Mg·(CAAC) (**2**) showing areas of electron density concentration (dashed lines) and depletion (solid lines). The BCP's are shown in blue.

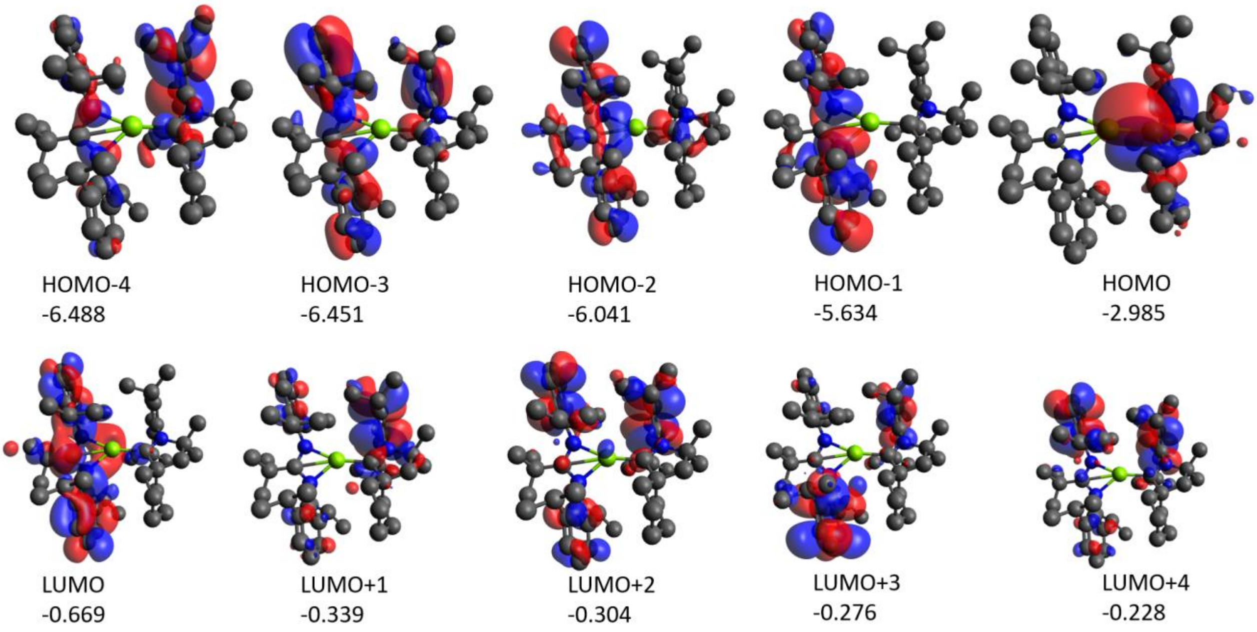

Figure S26. Selected MO's for complex (Am)Mg·(CAAC) (**2**), energies in eV.

Calculation of the spin density

For the radicals $[Be^{I}(CAAC)_2^{+}]$ and (CAAC-H)Be(CAAC) Mulliken spin densities at the BP86-D3(BJ)/def2SVP level of theory have been reported.[S20,S21] However, using the more flexible basis set def2TZVP, gave strikingly different values (see Table S3). Especially noticeable is the considerable drop of the Mulliken spin densities on the metals. Calculations at the B3PW91/def2SVP and B3PW91/def2TZVP levels of theory show the same trend. Since it is well-known that Mulliken population analysis is highly sensitive to the basis set used in the calculation,[S22] we calculated the spin densities for $[Be^{I}(CAAC)_2^{+}]$, (CAAC-H)Be(CAAC) and (Am)Mg·(CAAC) (**2**) with the NBO method (NBO7);[S17] see Tables S2 and S3. The spin densities on the metals are much lower and hardly affected by choice of the calculational method and basis set.

Table S2. The calculated spin density for (Am)Mg·(CAAC) (**2**); B3PW91/def2TZVP//def2SVP

Electron density **LMg-CAAC**

Atom No.	Charge	Core	Valence	Rydberg	Total	Spin density
Mg1	1.73353	9.99992	0.23115	0.03541	10.26647	0.01927
N2	-0.83881	1.99999	5.80603	0.03280	7.83881	0.00048
N3	-0.78895	1.99999	5.75635	0.03261	7.78895	0.00024
N4	-0.48283	1.99999	5.44745	0.03539	7.48283	0.19567
C5	0.52696	1.99999	3.44119	0.03187	5.47304	0.00359
C77	-0.56146	1.99998	4.52529	0.03619	6.56146	0.69345
C78	-0.12906	1.99999	4.10775	0.02133	6.12906	-0.02142
C79	-0.40459	1.99999	4.39110	0.01350	6.40459	0.02460
C91	-0.40305	1.99999	4.38775	0.01531	6.40305	0.01127
C94	-0.42239	1.99999	4.40805	0.01436	6.42239	0.00600
C97	0.14241	1.99999	3.84190	0.01570	5.85759	-0.01032
C106	0.14378	1.99999	3.83282	0.02341	5.85622	-0.00937
C107	0.00338	1.99999	3.97609	0.02054	5.99662	0.01396
C114	0.00642	1.99999	3.97338	0.02022	5.99358	0.00684
**TOTAL**	0.00000	119.99920	301.94808	1.05272	423.00000	1.00000

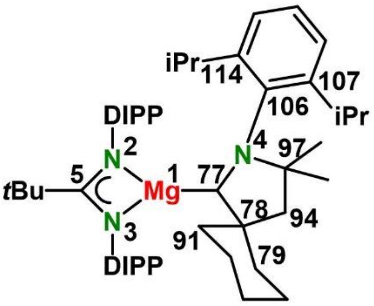

Table S3. Calculation of spin densities (Mulliken and NBO) and the NPA charges on the atoms Be, C1 and N1 using two different methods (BP86 and B3PW91) and two different basis sets (def2SVP and def2 TZVP). In all calculations corrections for dispersion have been included (GD3BJ).

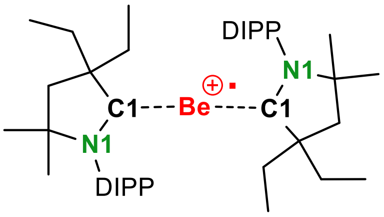

$Be(CAAC)_2^+$

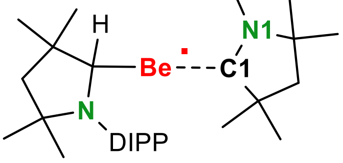

(CAAC-H)Be(CAAC)

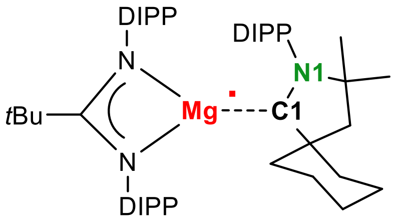

(Am)Mg(CAAC)

	Method and basis set	$Be(CAAC)_2^+$			(CAAC-H)Be(CAAC)			(Am)Mg(CAAC)		
		**Be**	**C1**	**N1**	**Be**	**C1**	**N1**	**Mg**	**C1**	**N1**
**Mulliken spin density**	BP86/def2SVP	0.38	0.18	0.13	0.23	0.54	0.19	0.19	0.58	0.18
	BP86/def2TZVP //BP86/def2SVP	0.11	0.30	0.12	0.07	0.64	0.19	0.11	0.71	0.19
	B3PW91/def2SVP	0.42	0.17	0.13	0.20	0.62	0.19	0.16	0.68	0.17
	B3PW91/ def2TZVP //B3PW91/def2SVP	0.15	0.29	0.12	0.05	0.70	0.18	0.07	0.80	0.18
**NBO spin density**	BP86/def2SVP	0.11	0.26	0.14	0.07	0.60	0.22	0.03	0.62	0.20
	BP86/ def2TZVP //BP86/def2SVP	0.11	0.27	0.13	0.06	0.61	0.21	0.03	0.63	0.20
	B3PW91/def2SVP	0.14	0.26	0.14	0.06	0.65	0.22	0.02	0.69	0.20
	B3PW91/ def2TZVP //B3PW91/def2SVP	0.13	0.27	0.14	0.05	0.65	0.21	0.02	0.69	0.20
**NPA atomic charges**	BP86/def2SVP	1.489	-0.400	-0.403	1.499	-0.588	-0.478	1.681	-0.573	-0.495
	BP86/ def2TZVP //BP86/def2SVP	1.584	-0.435	-0.356	1.507	-0.569	-0.426	1.705	-0.539	-0.457
	B3PW91/def2SVP	1.489	-0.380	-0.425	1.512	-0.588	-0.508	1.724	-0.601	-0.528
	B3PW91/ def2TZVP //B3PW91/def2SVP	1.581	-0.411	-0.375	1.513	-0.566	-0.448	1.734	-0.561	-0.483

XYZ-Coordinates

134
LMgCAAC (2)
Mg 0.007480 -0.021681 -0.032177
N 1.012605 1.719070 0.328006
N 1.998176 -0.245493 0.548069
N -2.825551 -1.208191 -0.735300
C 2.138715 1.072314 0.674070
C 3.351625 1.802397 1.298792
C 4.405724 0.843013 1.860073
H 3.982321 0.128510 2.576298
H 5.169703 1.435155 2.386649
H 4.911497 0.265264 1.080490
C 4.036329 2.748104 0.299718
H 4.443843 2.211450 -0.567400
H 4.879912 3.241768 0.806491
H 3.356114 3.530858 -0.056996
C 2.821055 2.652439 2.466707
H 2.154025 3.449080 2.116869
H 3.670197 3.125372 2.982991
H 2.279673 2.040174 3.202335
C 0.780016 3.057771 -0.003506
C 1.174630 3.538095 -1.278359
C 0.809298 4.833920 -1.649727
H 1.110124 5.217784 -2.626776
C 0.049863 5.640110 -0.803597
H -0.227711 6.650677 -1.112643
C -0.367768 5.144940 0.427786
H -0.984857 5.769977 1.077365
C -0.020301 3.855974 0.844329
C 1.897545 2.621906 -2.250474
H 2.458884 1.890564 -1.655132
C 0.899580 1.822428 -3.093255
H 0.214653 1.224879 -2.469825
H 1.423780 1.127635 -3.766802
H 0.277968 2.492998 -3.706103
C 2.905370 3.341994 -3.142421
H 2.411870 4.005289 -3.870065
H 3.492853 2.611243 -3.719597
H 3.604807 3.952269 -2.551773
C -0.563281 3.283160 2.138627
H 0.071727 2.422120 2.393799
C -1.981934 2.751993 1.926299
H -2.663807 3.555492 1.609398
H -2.385377 2.310213 2.849205
H -2.023120 1.976334 1.146867
C -0.511284 4.265729 3.306800
H 0.500671 4.675071 3.447219
H -0.809988 3.765903 4.241421
H -1.198507 5.114075 3.160441
C 2.857784 -1.338933 0.644046
C 2.556192 -2.343340 1.600713
C 3.322075 -3.511882 1.615151
H 3.105741 -4.288148 2.350005
C 4.351426 -3.714968 0.699494
H 4.935504 -4.637689 0.724526
C 4.616176 -2.741853 -0.258101
H 5.407830 -2.912643 -0.991458
C 3.884845 -1.551965 -0.309411
C 1.403891 -2.146197 2.570742
H 0.560625 -1.751743 1.971627
C 0.919502 -3.439136 3.219442
H 0.694931 -4.214545 2.472794
H 0.001647 -3.251867 3.796296
H 1.664454 -3.846432 3.921229
C 1.712796 -1.098220 3.642094
H 2.580824 -1.402551 4.247983
H 0.854083 -0.967667 4.318827
H 1.934569 -0.122603 3.192945
C 4.170586 -0.543696 -1.407181
H 3.688750 0.397907 -1.115458
C 3.534342 -0.969003 -2.732162
H 3.931174 -1.941040 -3.065063
H 3.742100 -0.228654 -3.520937
H 2.445719 -1.066184 -2.636389
C 5.660955 -0.255712 -1.591090
H 6.145689 0.020923 -0.643087
H 5.805364 0.573908 -2.300874
H 6.198041 -1.126844 -1.997486
C -1.447618 -1.294793 -0.667106
C -1.041723 -2.629249 -1.269362
C -0.715416 -3.650635 -0.154122
H 0.035910 -3.181221 0.499301
H -1.601401 -3.820676 0.475903
C -0.151535 -4.973761 -0.664241
H 0.087219 -5.630063 0.189508
H -0.916839 -5.508309 -1.255555
C 1.088694 -4.745454 -1.522709
H 1.473694 -5.702156 -1.912491
H 1.889099 -4.314886 -0.894448
C 0.783103 -3.781956 -2.664706
H 0.053428 -4.240804 -3.356195
H 1.691359 -3.586855 -3.257673
C 0.222269 -2.469959 -2.128151
H 0.017457 -1.756085 -2.943060
H 1.001957 -2.009686 -1.491084
C -2.279677 -3.025035 -2.100163
H -2.151096 -2.654154 -3.129242
H -2.439806 -4.111279 -2.166697
C -3.491288 -2.318159 -1.460040
C -4.268391 -3.232535 -0.506608
H -3.615812 -3.693723 0.245075
H -4.758408 -4.040580 -1.070950
H -5.051242 -2.664170 0.017325
C -4.462233 -1.803155 -2.520852
H -5.273760 -1.217791 -2.062662
H -4.918266 -2.649841 -3.056569
H -3.948409 -1.171974 -3.257826
C -3.556858 -0.219192 -0.029966
C -3.973951 0.959525 -0.690399
C -4.817022 1.849664 -0.014179
H -5.158655 2.754360 -0.522965
C -5.202428 1.616678 1.300725
H -5.869149 2.316519 1.810583
C -4.690591 0.515713 1.981320
H -4.939348 0.371435 3.035653
C -3.850083 -0.401540 1.343299
C -3.427061 1.347056 -2.050510
H -2.857460 0.485040 -2.427017
C -2.441924 2.510235 -1.888638
H -2.932494 3.401046 -1.466947
H -2.005093 2.794109 -2.857269
H -1.612714 2.254152 -1.213797
C -4.514958 1.704172 -3.063793
H -5.254119 0.898110 -3.172152
H -4.071647 1.896564 -4.053450
H -5.057184 2.616373 -2.768218
C -3.191285 -1.497295 2.157681
H -2.662988 -2.151361 1.453091
C -2.127593 -0.883105 3.072595
H -1.393016 -0.305210 2.491132
H -1.582860 -1.666692 3.619840
H -2.574700 -0.199943 3.811917
C -4.177769 -2.339642 2.965307
H -4.677074 -1.746034 3.747447
H -3.654051 -3.167571 3.468800
H -4.959678 -2.772215 2.325320

59
CAAC
N -0.078080 -0.067834 0.177740
C 0.767968 -0.090855 -0.821140
C 2.149047 -0.181264 -0.208031
C 2.846125 1.175868 -0.452526
H 2.767126 1.404280 -1.528543
H 2.300798 1.978189 0.070208
C 4.313049 1.168047 -0.031116
H 4.771053 2.143131 -0.265039
H 4.386236 1.052671 1.065192
C 5.086850 0.039292 -0.707243
H 6.131025 0.023379 -0.354121
H 5.127499 0.230953 -1.794790
C 4.415902 -1.310628 -0.466805
H 4.475461 -1.564308 0.607271
H 4.957325 -2.109071 -1.000174
C 2.957255 -1.283144 -0.912033
H 2.474161 -2.258722 -0.734977
H 2.900128 -1.102929 -1.998537
C 1.931554 -0.481452 1.296392
H 2.092041 -1.553814 1.487812
H 2.620630 0.064913 1.955858
C 0.470035 -0.119217 1.594166
C 0.323002 1.241830 2.274799
H 0.884082 2.026870 1.751150
H 0.711207 1.179401 3.302322
H -0.732931 1.544222 2.331883
C -0.242282 -1.165779 2.445205
H -1.319265 -0.955375 2.523431
H 0.179601 -1.152931 3.461347
H -0.113428 -2.179224 2.044142
C -1.493757 0.040127 -0.036741
C -2.252472 -1.140828 -0.151265
C -3.640870 -1.021591 -0.270626
H -4.251860 -1.922393 -0.355705
C -4.252545 0.228278 -0.305009
H -5.339616 0.302885 -0.388341
C -3.475526 1.381980 -0.278260
H -3.957421 2.357382 -0.370526
C -2.082437 1.312750 -0.160940
C -1.582986 -2.493110 -0.303085
H -0.572458 -2.415094 0.118068
C -1.404098 -2.789810 -1.796725
H -2.379688 -2.847610 -2.305455
H -0.882762 -3.749410 -1.943967
H -0.810987 -1.993865 -2.271862
C -2.305152 -3.623304 0.425419
H -2.457541 -3.386842 1.489710
H -1.719621 -4.553554 0.362202
H -3.291697 -3.834443 -0.016016
C -1.240642 2.562176 -0.333020
H -0.241032 2.349707 0.062768
C -1.061476 2.834065 -1.831318
H -0.592144 1.965766 -2.317388
H -0.418412 3.714283 -1.992653
H -2.032159 3.024930 -2.316463
C -1.785168 3.779718 0.409484
H -2.746458 4.120812 -0.005740
H -1.082002 4.622817 0.326190
H -1.938593 3.568935 1.479079

150
LMg-MgL
Mg -1.381354 -0.487100 0.096166
N -3.322176 -1.127842 0.426080
N -2.843457 1.003632 0.109987

C -3.804576 0.123806 0.383602
C -5.272248 0.468580 0.739521
C -5.557946 1.974240 0.757690
H -4.872082 2.525365 1.413180
H -6.580392 2.126474 1.136074
H -5.500948 2.429916 -0.237152
C -6.255321 -0.194116 -0.238847
H -6.091872 0.135842 -1.274689
H -7.280063 0.096169 0.040052
H -6.196831 -1.288523 -0.205559
C -5.559346 -0.075580 2.150242
H -5.502170 -1.169629 2.186389
H -6.577895 0.217544 2.447011
H -4.863317 0.338111 2.894100
C -3.967772 -2.359888 0.557665
C -4.484835 -2.999021 -0.595749
C -5.069804 -4.260628 -0.462515
H -5.485841 -4.757246 -1.341617
C -5.116341 -4.904216 0.772767
H -5.583544 -5.887929 0.861351
C -4.537688 -4.301855 1.886797
H -4.540052 -4.828802 2.843502
C -3.945718 -3.038246 1.798411
C -4.290047 -2.354551 -1.955515
H -4.276682 -1.266241 -1.803660
C -2.913682 -2.741828 -2.508894
H -2.107313 -2.476171 -1.805548
H -2.708443 -2.232824 -3.463778
H -2.850844 -3.828455 -2.676353
C -5.395057 -2.662794 -2.960684
H -5.402106 -3.724105 -3.255157
H -5.249746 -2.074447 -3.880008
H -6.388397 -2.419415 -2.554332
C -3.203143 -2.430345 2.972695
H -3.188204 -1.342187 2.811724
C -1.745638 -2.906335 2.962906
H -1.687668 -3.996664 3.108581
H -1.162056 -2.420851 3.761644
H -1.252771 -2.680594 2.002849
C -3.860510 -2.691001 4.325216
H -4.915181 -2.376692 4.327367
H -3.337965 -2.135161 5.119143
H -3.826738 -3.756733 4.601517
C -2.834294 2.361844 -0.192027
C -2.131317 3.239689 0.667333
C -2.080532 4.598783 0.341864
H -1.551908 5.289470 1.000466
C -2.678073 5.084056 -0.818606
H -2.635067 6.150127 -1.053221
C -3.290667 4.197257 -1.702437
H -3.710111 4.577189 -2.635926
C -3.359361 2.831647 -1.421097
C -1.446893 2.688182 1.904791
H -1.001478 1.720645 1.599551
C -0.300826 3.554801 2.410960
H 0.431347 3.764397 1.619530
H 0.226195 3.041543 3.228239
H -0.658585 4.516443 2.812122
C -2.445831 2.386947 3.023628
H -2.948812 3.309629 3.354388
H -1.937236 1.945448 3.895509
H -3.217141 1.681261 2.689633
C -3.911696 1.835386 -2.422731
H -4.406584 1.038518 -1.850687
C -2.766317 1.170842 -3.192832
H -2.235705 1.908344 -3.814153
H -3.141713 0.371984 -3.851457
H -2.029126 0.726671 -2.505614
C -4.954011 2.412172 -3.374437
H -5.770871 2.906806 -2.826705
H -5.392971 1.611805 -3.990029
H -4.517142 3.149587 -4.066171
Mg 1.381757 -0.486078 -0.088331
N 3.320835 -1.129608 -0.423785
N 2.844962 1.002771 -0.109676
C 3.804705 0.121529 -0.383952
C 5.272144 0.464449 -0.742385
C 5.559704 1.969770 -0.760826
H 4.873887 2.521844 -1.415539
H 6.581955 2.120729 -1.140242
H 5.504284 2.425402 0.234137
C 6.255865 -0.199382 0.234534
H 6.094262 0.130855 1.270582
H 7.280545 0.089670 -0.045853
H 6.196048 -1.293722 0.201433
C 5.556460 -0.080083 -2.153547
H 5.498000 -1.174068 -2.189544
H 6.574899 0.211901 -2.451818
H 4.859789 0.334383 -2.896346
C 3.964499 -2.362410 -0.558054
C 4.483280 -3.003370 0.593534
C 5.066568 -4.265494 0.457686
H 5.483956 -4.763513 1.335352
C 5.109742 -4.907772 -0.778383
H 5.575715 -5.891877 -0.869075
C 4.529237 -4.303619 -1.890495
H 4.528903 -4.829569 -2.847750
C 3.938802 -3.039496 -1.799450
C 4.292352 -2.360020 1.954375
H 4.279584 -1.271568 1.803428
C 2.917001 -2.746565 2.510740
H 2.109196 -2.479715 1.809498
H 2.714474 -2.238132 3.466512
H 2.853628 -3.833266 2.677526
C 5.399522 -2.670194 2.956560
H 5.406305 -3.731786 3.250023
H 5.256999 -2.082578 3.876790
H 6.392095 -2.427360 2.548013
C 3.193748 -2.429788 -2.971232
H 3.180571 -1.341724 -2.809514
C 1.735684 -2.904047 -2.957508
H 1.676014 -3.994183 -3.103952
H 1.150294 -2.417184 -3.754080
H 1.245995 -2.678543 -1.995780
C 3.846837 -2.690367 -4.325811
H 4.901878 -2.377323 -4.330878
H 3.322640 -2.133389 -5.117849
H 3.810966 -3.755873 -4.602719
C 2.837862 2.360926 0.192782
C 2.135217 3.239778 -0.665801
C 2.085354 4.598664 -0.339352
H 1.556910 5.290148 -0.997262
C 2.683578 5.082736 0.821288
H 2.641229 6.148652 1.056732
C 3.296200 4.195006 1.704156
H 3.716348 4.574069 2.637686
C 3.364019 2.829542 1.421801
C 1.449903 2.689380 -1.903290
H 1.003792 1.722121 -1.598305
C 0.304696 3.557396 -2.408894
H -0.427076 3.767458 -1.617256
H -0.223060 3.045028 -3.226254
H 0.663414 4.518807 -2.809771
C 2.448215 2.387639 -3.022556
H 2.952206 3.309915 -3.352917
H 1.938856 1.947260 -3.894554
H 3.218700 1.680804 -2.689172
C 3.916238 1.832299 2.422511
H 4.409633 1.035043 1.849704
C 2.770971 1.168874 3.193721
H 2.242102 1.906764 3.816079
H 3.146086 0.369149 3.851454
H 2.032112 0.726067 2.507361
C 4.960244 2.407597 3.373301
H 5.777036 2.901502 2.824819
H 5.398982 1.606531 3.988143
H 4.524958 3.145270 4.065753

135
LMg(H)CAAC
Mg -0.163818 -0.048837 -0.668960
N -1.612988 -1.291806 0.202546
N -1.667258 0.900938 0.473342
N 3.013946 0.253208 -0.754644
C -2.312689 -0.252589 0.666567
C -3.663943 -0.412064 1.412904
C -4.263423 0.910785 1.902429
H -3.576528 1.483573 2.534497
H -5.158701 0.681600 2.500874
H -4.573510 1.562299 1.078111
C -4.712122 -1.096619 0.524938
H -4.912285 -0.527556 -0.392997
H -5.658323 -1.165284 1.083756
H -4.411903 -2.113910 0.250659
C -3.433079 -1.315365 2.636260
H -3.145498 -2.329587 2.336361
H -4.369019 -1.387828 3.211329
H -2.659378 -0.916657 3.306940
C -1.906967 -2.656607 0.110367
C -2.579685 -3.164645 -1.031840
C -2.721239 -4.548730 -1.169036
H -3.238171 -4.946098 -2.045728
C -2.209241 -5.430443 -0.221365
H -2.332110 -6.508709 -0.349000
C -1.530224 -4.926505 0.883717
H -1.111867 -5.618060 1.619086
C -1.362516 -3.550706 1.064704
C -3.096757 -2.246598 -2.126507
H -3.133216 -1.230675 -1.708654
C -2.132291 -2.202025 -3.314897
H -1.165570 -1.764775 -3.026758
H -2.545989 -1.575042 -4.121279
H -1.965196 -3.212571 -3.721935
C -4.505440 -2.613261 -2.598612
H -4.508534 -3.554078 -3.171558
H -4.897967 -1.829793 -3.265747
H -5.208020 -2.731693 -1.761233
C -0.537371 -3.032078 2.227746
H -0.759908 -1.960017 2.328327
C 0.953510 -3.156087 1.913877
H 1.228264 -4.200975 1.702606
H 1.567904 -2.809530 2.757292
H 1.234572 -2.561732 1.033820
C -0.869564 -3.709945 3.556750
H -1.946947 -3.673582 3.775759
H -0.336888 -3.213580 4.383196
H -0.563142 -4.768022 3.563975
C -2.074053 2.231690 0.540904
C -1.463528 3.091210 1.490440
C -1.783176 4.452172 1.469578
H -1.330724 5.123492 2.200815
C -2.659333 4.976773 0.522710
H -2.890338 6.044544 0.519668
C -3.228002 4.132569 -0.426578
H -3.903903 4.548735 -1.176846
C -2.953932 2.762578 -0.436900
C -0.463618 2.531643 2.488950
H 0.184934 1.850122 1.910758
C 0.417065 3.597857 3.135838
H 0.886680 4.260714 2.394975
H 1.219550 3.123715 3.721039
H -0.158443 4.228842 3.831636
C -1.117345 1.677359 3.576096

H -1.845845 2.264581 4.157465
H -0.358155 1.291592 4.274621
H -1.635274 0.816504 3.140724
C -3.573471 1.864152 -1.492972
H -3.574921 0.843754 -1.087540
C -2.728878 1.820015 -2.768848
H -2.593524 2.830403 -3.186500
H -3.216262 1.195397 -3.534037
H -1.734682 1.386726 -2.587606
C -5.026161 2.218234 -1.808270
H -5.635063 2.290589 -0.894076
H -5.472837 1.447031 -2.454976
H -5.110693 3.176875 -2.343977
C 1.846015 0.838701 -0.655537
C 1.972193 2.237284 -1.227860
C 2.097432 3.179956 -0.005361
H 1.224837 2.998409 0.635362
H 2.982068 2.920797 0.595341
C 2.133133 4.655731 -0.389499
H 2.190333 5.264745 0.527764
H 3.054969 4.874973 -0.957606
C 0.914096 5.046520 -1.217158
H 0.975567 6.105126 -1.517706
H 0.007276 4.943344 -0.596793
C 0.781502 4.142128 -2.436574
H 1.626925 4.314428 -3.127390
H -0.132530 4.388689 -2.999606
C 0.737222 2.675736 -2.027146
H 0.612844 2.012642 -2.894309
H -0.154004 2.529673 -1.391234
C 3.257890 2.178050 -2.071844
H 2.987415 1.969166 -3.117751
H 3.830990 3.115267 -2.062562
C 4.084532 1.010693 -1.512772
C 5.209163 1.454455 -0.578517
H 4.863061 2.156795 0.189826
H 5.987911 1.959727 -1.168026
H 5.671342 0.589599 -0.081033
C 4.671529 0.147528 -2.620715
H 5.198773 -0.727241 -2.213449
H 5.396552 0.744012 -3.194267
H 3.891453 -0.196346 -3.311471
C 3.348481 -0.993877 -0.118397
C 3.259858 -2.208265 -0.827686
C 3.768781 -3.360681 -0.215129
H 3.715224 -4.311247 -0.749769
C 4.308240 -3.324040 1.063979
H 4.704835 -4.235001 1.518324
C 4.288288 -2.135306 1.786518
H 4.644019 -2.125830 2.818586
C 3.797072 -0.956024 1.220852
C 2.521065 -2.349105 -2.145125
H 2.222447 -1.346564 -2.487051
C 1.223339 -3.131969 -1.914509
H 1.423892 -4.151488 -1.550777
H 0.650015 -3.205976 -2.848379
H 0.569279 -2.650281 -1.174552
C 3.352814 -3.026353 -3.236047
H 4.316374 -2.524323 -3.399717
H 2.800175 -3.023368 -4.187922
H 3.564347 -4.078119 -2.986755
C 3.629755 0.276140 2.088317
H 3.475092 1.135446 1.426832
C 2.353084 0.132342 2.922982
H 1.480036 -0.045551 2.279243
H 2.167300 1.045801 3.507008
H 2.432018 -0.711398 3.625800
C 4.839469 0.582347 2.968247
H 4.993986 -0.188869 3.738704
H 4.694304 1.538591 3.494142
H 5.764315 0.655730 2.377055
H 0.169035 -0.191583 -2.404700

75
LMg Radical
Mg -0.053572 0.531319 -2.215979
N 1.058882 0.198552 -0.482804
N -1.151741 0.258657 -0.450906
C -0.041316 0.111361 0.276165
C 0.011797 -0.023089 1.820715
C -1.366743 -0.039205 2.491342
H -1.982075 0.826310 2.218227
H -1.216176 -0.013669 3.581473
H -1.943044 -0.941034 2.256880
C 0.758069 -1.296548 2.248900
H 0.272030 -2.206270 1.869022
H 0.750184 -1.355275 3.348156
H 1.804499 -1.293754 1.922721
C 0.780639 1.191215 2.372548
H 1.828326 1.192664 2.050882
H 0.767456 1.152301 3.472356
H 0.316751 2.140029 2.067045
C 2.414073 0.074171 -0.157920
C 3.019550 -1.206067 -0.182593
C 4.388240 -1.309967 0.077434
H 4.863020 -2.293359 0.075372
C 5.161191 -0.177769 0.326347
H 6.229116 -0.277351 0.534633
C 4.571787 1.082654 0.280651
H 5.189139 1.969707 0.438341
C 3.205084 1.232180 0.026433
C 2.200626 -2.417147 -0.587409
H 1.158713 -2.215758 -0.301910
C 2.223458 -2.561146 -2.112771
H 1.867750 -1.641519 -2.606602
H 1.582719 -3.392853 -2.445795
H 3.247181 -2.748494 -2.473217
C 2.621545 -3.714620 0.095917
H 3.619108 -4.048959 -0.229962
H 1.915010 -4.522291 -0.150834
H 2.644402 -3.605099 1.190626
C 2.585743 2.605631 -0.148329
H 1.509164 2.500452 0.051887
C 2.730815 3.051835 -1.607414
H 3.792229 3.163593 -1.879999
H 2.227090 4.016088 -1.782335
H 2.294925 2.311643 -2.299449
C 3.134608 3.660772 0.808501
H 3.063386 3.331381 1.856042
H 2.569638 4.600426 0.707925
H 4.190524 3.895625 0.601236
C -2.492721 -0.004321 -0.172505
C -3.423919 1.057235 -0.254669
C -4.775685 0.783785 -0.023982
H -5.504249 1.595389 -0.064490
C -5.212544 -0.509416 0.249584
H -6.271443 -0.703076 0.436234
C -4.297754 -1.560529 0.251498
H -4.653080 -2.578295 0.424292
C -2.938880 -1.334338 0.025283
C -2.940323 2.453966 -0.600285
H -2.137462 2.319687 -1.350149
C -4.011708 3.333740 -1.238070
H -4.493487 2.832407 -2.091036
H -3.565442 4.272179 -1.601414
H -4.798459 3.608817 -0.517561
C -2.305410 3.154524 0.602765
H -3.038964 3.276107 1.415663
H -1.933956 4.154204 0.326325
H -1.458088 2.576681 0.993095
C -1.954439 -2.483097 -0.087561
H -0.986325 -2.125547 0.289256
C -1.741976 -2.849087 -1.559354
H -2.678935 -3.208127 -2.012841
H -0.979304 -3.635986 -1.668948
H -1.410853 -1.975057 -2.144234
C -2.323508 -3.710537 0.739239
H -2.504254 -3.447353 1.792763
H -1.508220 -4.449820 0.708295
H -3.227455 -4.210154 0.356993

134
LMgCAAC without dispersion
Mg 0.054430 -0.106795 -0.087690
N 1.151085 1.650157 0.158143
N 2.085568 -0.338525 0.504320
N -2.981828 -1.166094 -0.508129
C 2.255858 0.987958 0.561819
C 3.512218 1.723548 1.127461
C 4.557976 0.795075 1.763319
H 4.139416 0.162464 2.555808
H 5.338675 1.424144 2.219446
H 5.047588 0.135390 1.040255
C 4.218253 2.547230 0.034453
H 4.563287 1.920355 -0.799447
H 5.107611 3.026965 0.473580
H 3.576795 3.342584 -0.364748
C 3.053116 2.693826 2.231369
H 2.413870 3.491217 1.837074
H 3.938652 3.168952 2.681715
H 2.511113 2.173527 3.035114
C 0.959419 3.002551 -0.199405
C 1.327821 3.444407 -1.503353
C 1.041695 4.760090 -1.882093
H 1.330022 5.105275 -2.877604
C 0.384838 5.637856 -1.024135
H 0.172868 6.662856 -1.339013
C -0.014305 5.189782 0.229438
H -0.553445 5.870633 0.893137
C 0.250378 3.883581 0.660404
C 1.954858 2.492572 -2.514897
H 2.461559 1.698652 -1.947871
C 0.874874 1.810785 -3.365704
H 0.141964 1.269408 -2.746520
H 1.323463 1.085819 -4.064089
H 0.315550 2.552013 -3.958549
C 3.005572 3.146818 -3.416229
H 2.556976 3.864193 -4.121779
H 3.516091 2.382029 -4.022727
H 3.769956 3.683921 -2.834682
C -0.291306 3.427573 2.007445
H 0.208550 2.477460 2.250235
C -1.795412 3.147599 1.911913
H -2.350281 4.060264 1.641806
H -2.191924 2.787873 2.873938
H -2.030549 2.388710 1.150739
C -0.007822 4.411061 3.148454
H 1.059552 4.667112 3.222085
H -0.318257 3.976963 4.112294
H -0.567588 5.352159 3.026340
C 2.952513 -1.421320 0.745834
C 2.694068 -2.274654 1.855763
C 3.517061 -3.386807 2.065959
H 3.335620 -4.031883 2.927919
C 4.558420 -3.697917 1.197834
H 5.191116 -4.569767 1.382265
C 4.766311 -2.899563 0.078974
H 5.562152 -3.162959 -0.622751
C 3.980408 -1.769868 -0.175842
C 1.542243 -1.999738 2.813999
H 0.749015 -1.512730 2.218179
C 0.931814 -3.270737 3.410309

H 0.681503 -4.008060 2.633103
H 0.007230 -3.027225 3.955817
H 1.607663 -3.755518 4.132841
C 1.929631 -1.024675 3.932200
H 2.757737 -1.430065 4.536112
H 1.077935 -0.842645 4.607201
H 2.249748 -0.053164 3.532016
C 4.237143 -0.988121 -1.458467
H 3.660126 -0.054000 -1.396113
C 3.722579 -1.755316 -2.683447
H 4.261271 -2.708481 -2.808410
H 3.864763 -1.166870 -3.604525
H 2.653304 -1.989458 -2.592747
C 5.710906 -0.613504 -1.660933
H 6.132640 -0.087116 -0.792057
H 5.823021 0.042761 -2.538801
H 6.335547 -1.502389 -1.842850
C -1.593388 -1.274133 -0.626158
C -1.300577 -2.592053 -1.353348
C -0.723913 -3.662689 -0.388329
H 0.171668 -3.231194 0.094962
H -1.436716 -3.858901 0.427518
C -0.325165 -4.971363 -1.075058
H 0.111501 -5.662000 -0.334005
H -1.224398 -5.479955 -1.467287
C 0.659979 -4.729868 -2.216865
H 0.882992 -5.673806 -2.742216
H 1.618578 -4.376316 -1.796182
C 0.127267 -3.685689 -3.196797
H -0.752191 -4.091544 -3.728932
H 0.878211 -3.471764 -3.976234
C -0.267145 -2.391749 -2.481880
H -0.653042 -1.649275 -3.200066
H 0.647192 -1.945815 -2.041420
C -2.674055 -2.994266 -1.933981
H -2.754581 -2.608093 -2.963268
H -2.831556 -4.081787 -1.990453
C -3.748216 -2.318815 -1.066451
C -4.276684 -3.257870 0.029544
H -3.476010 -3.637374 0.677663
H -4.777967 -4.124877 -0.429022
H -5.017516 -2.743536 0.658445
C -4.947522 -1.889394 -1.915150
H -5.682125 -1.322133 -1.323467
H -5.456577 -2.783325 -2.308302
H -4.640403 -1.277781 -2.773060
C -3.652828 -0.096030 0.157075
C -4.122369 1.017674 -0.591621
C -4.916825 1.979100 0.046928
H -5.295507 2.827557 -0.528869
C -5.228432 1.880881 1.397981
H -5.863935 2.632481 1.873944
C -4.696060 0.836737 2.145500
H -4.903170 0.787472 3.217841
C -3.893746 -0.149583 1.556625
C -3.737556 1.250673 -2.045753
H -3.221664 0.343619 -2.393543
C -2.738411 2.410438 -2.150833
H -3.186300 3.359869 -1.816006
H -2.408111 2.546512 -3.192732
H -1.844101 2.236142 -1.535173
C -4.939695 1.514772 -2.960615
H -5.705979 0.730625 -2.879702
H -4.616349 1.566402 -4.012740
H -5.424603 2.476146 -2.726714
C -3.252009 -1.189907 2.463517
H -2.746116 -1.919419 1.816592
C -2.170901 -0.534855 3.333599
H -1.412818 -0.026991 2.718034
H -1.657169 -1.288481 3.950648
H -2.600768 0.217174 4.014759
C -4.255165 -1.933431 3.352768
H -4.719435 -1.262645 4.093423
H -3.749905 -2.735027 3.915147
H -5.066227 -2.392597 2.769781

75
LMg radical without dispersion
Mg -0.006121 0.001084 -2.220932
N 1.093801 0.000378 -0.434697
N -1.125957 0.000382 -0.440566
C -0.021745 0.000141 0.315683
C 0.002949 0.000079 1.871814
C -1.388692 -0.000036 2.518540
H -1.980336 0.885734 2.255458
H -1.258391 0.000029 3.612346
H -1.980121 -0.885973 2.255536
C 0.742993 -1.252999 2.373777
H 0.257979 -2.176825 2.023094
H 0.719889 -1.266061 3.475029
H 1.794956 -1.267854 2.063488
C 0.742736 1.253442 2.373484
H 1.794723 1.268305 2.063267
H 0.719534 1.266875 3.474730
H 0.257639 2.177082 2.022427
C 2.463025 0.000010 -0.113218
C 3.175701 -1.228539 -0.046159
C 4.553740 -1.201843 0.195847
H 5.104358 -2.143192 0.263258
C 5.242366 -0.000717 0.337593
H 6.317664 -0.001005 0.533483
C 4.554389 1.200757 0.195779
H 5.105504 2.141823 0.263139
C 3.176346 1.228176 -0.046193
C 2.479853 -2.550672 -0.337533
H 1.408703 -2.406320 -0.131468
C 2.609668 -2.892638 -1.829328
H 2.216866 -2.083975 -2.468337
H 2.057917 -3.814580 -2.075789
H 3.664849 -3.042756 -2.109358
C 2.961227 -3.717602 0.527736
H 4.000858 -4.001991 0.299593
H 2.339908 -4.609096 0.346953
H 2.907592 -3.480546 1.601212
C 2.481229 2.550717 -0.337462
H 1.409948 2.406801 -0.131756
C 2.611670 2.893025 -1.829121
H 3.667009 3.042674 -2.108809
H 2.060475 3.815325 -2.075489
H 2.218628 2.084746 -2.468468
C 2.962912 3.717174 0.528282
H 2.908770 3.479871 1.601677
H 2.342134 4.609048 0.347510
H 4.002780 4.001057 0.300588
C -2.503210 0.000061 -0.159263
C -3.211277 1.228517 -0.099989
C -4.602090 1.201599 0.048477
H -5.154698 2.143032 0.099532
C -5.299463 -0.000574 0.128574
H -6.386084 -0.000826 0.246232
C -4.601545 -1.202434 0.048428
H -5.153734 -2.144116 0.099420
C -3.210724 -1.228721 -0.100009
C -2.481467 2.555016 -0.250602
H -1.426487 2.372182 0.007730
C -2.514274 3.024184 -1.711906
H -2.095981 2.263902 -2.392714
H -1.935394 3.952530 -1.846780
H -3.548410 3.217877 -2.039903
C -2.994248 3.652016 0.686254
H -4.018070 3.970047 0.432721
H -2.354613 4.546204 0.615873
H -2.998586 3.321131 1.736401
C -2.480323 -2.554878 -0.250753
H -1.425443 -2.371648 0.007697
C -2.512768 -3.023788 -1.712144
H -3.546791 -3.217777 -2.040322
H -1.933545 -3.951905 -1.847112
H -2.094643 -2.263227 -2.392743
C -2.992718 -3.652239 0.685899
H -2.997390 -3.321456 1.736077
H -2.352634 -4.546105 0.615526
H -4.016342 -3.970753 0.432170

59
CAAC without dispersion
N -0.069188 -0.041278 0.199863
C 0.781083 -0.054781 -0.798311
C 2.171808 -0.131000 -0.194082
C 2.887279 1.211687 -0.484689
H 2.784490 1.418880 -1.563668
H 2.369336 2.035936 0.033333
C 4.368063 1.198417 -0.103033
H 4.826742 2.163963 -0.374969
H 4.473105 1.112771 0.994008
C 5.116592 0.046104 -0.770883
H 6.168431 0.026962 -0.439706
H 5.138068 0.212659 -1.863474
C 4.436608 -1.291568 -0.483860
H 4.529240 -1.527681 0.592379
H 4.952163 -2.106888 -1.018589
C 2.962957 -1.260737 -0.886505
H 2.482347 -2.230665 -0.672637
H 2.878261 -1.111469 -1.976451
C 1.963070 -0.394720 1.322329
H 2.161733 -1.454278 1.549457
H 2.635161 0.194411 1.963946
C 0.487795 -0.071213 1.622279
C 0.311021 1.289252 2.303580
H 0.842478 2.091188 1.772821
H 0.716064 1.240419 3.326143
H -0.751529 1.564430 2.380186
C -0.186125 -1.135250 2.487875
H -1.265396 -0.948453 2.595288
H 0.257983 -1.115326 3.495202
H -0.045272 -2.147290 2.085268
C -1.493192 0.024561 -0.034826
C -2.221439 -1.180751 -0.161072
C -3.611981 -1.100081 -0.304282
H -4.195242 -2.019100 -0.396307
C -4.262908 0.128386 -0.354871
H -5.350541 0.169708 -0.457008
C -3.519299 1.302928 -0.320892
H -4.029998 2.262814 -0.426682
C -2.125442 1.279986 -0.179553
C -1.534471 -2.532351 -0.293644
H -0.514802 -2.430509 0.101523
C -1.388380 -2.882737 -1.782181
H -2.374214 -2.982833 -2.264763
H -0.851540 -3.837515 -1.906300
H -0.824871 -2.098102 -2.309130
C -2.221923 -3.656646 0.484504
H -2.352147 -3.400037 1.547265
H -1.622882 -4.579426 0.429343
H -3.215945 -3.897048 0.075129
C -1.341564 2.574261 -0.339655
H -0.325263 2.399754 0.034494
C -1.202762 2.901185 -1.834265
H -0.710178 2.072419 -2.364358
H -0.598332 3.811787 -1.978097
H -2.188165 3.072092 -2.297531
C -1.925867 3.753937 0.441148
H -2.909151 4.063064 0.052317

H -1.261868 4.629265 0.361892
H -2.049487 3.519841 1.509977

105
Be(CAAC)2
Be -0.000001 -0.000086 -0.000194
C -1.153688 1.058830 -0.425881
C 1.153720 -1.058799 0.425961
N -2.499231 0.810857 -0.481675
N 2.499231 -0.810719 0.481832
C -3.336146 1.933408 -0.991611
C 3.336231 -1.933130 0.991917
C -2.249354 2.837234 -1.595993
C 2.249476 -2.837214 1.595996
C -0.930205 2.497913 -0.869881
C 0.930317 -2.497914 0.869873
C -0.690276 3.390142 0.361590
C 0.690401 -3.390051 -0.361665
C 0.276440 2.638095 -1.797226
C -0.276322 -2.638184 1.797201
H -0.627497 4.451822 0.068147
H 0.627910 -4.451781 -0.068349
H -1.485433 3.290115 1.112412
H 1.485403 -3.289743 -1.112610
H 0.255494 3.109824 0.848276
H -0.255513 -3.109898 -0.848166
H 0.430397 3.690461 -2.087077
H -0.430258 -3.690576 2.086971
H 1.196695 2.293809 -1.301682
H -1.196582 -2.293872 1.301680
H 0.144774 2.044788 -2.712122
H -0.144657 -2.044939 2.712140
C -3.034362 -0.368601 0.120675
C 3.034330 0.368640 -0.120705
C -3.259674 -0.364197 1.526927
C 3.259709 0.363893 -1.526931
C -3.823406 -1.487942 2.134354
C 3.823297 1.487565 -2.134622
C -4.125811 -2.627037 1.397866
C 4.125555 2.626871 -1.398394
C -3.274558 -1.549065 -0.628324
C 3.274446 1.549262 0.628051
C -3.830041 -2.652041 0.044709
C 3.829804 2.652148 -0.045237
C -2.860749 0.790256 2.428886
C 2.860945 -0.790882 -2.428556
H -4.007885 -1.475982 3.210838
H 4.007797 1.475373 -3.211101
H -4.559449 -3.503929 1.884552
H 4.559092 3.503702 -1.885281
C -2.973979 -1.833446 -2.103081
C 2.973853 1.833893 2.102757
H -4.014718 -3.566780 -0.525412
H 4.014389 3.567036 0.524675
C -4.254007 -1.928406 -2.944688
C 4.253864 1.928573 2.944422
C -1.896399 -1.015122 -2.800317
C 1.896054 1.015889 2.800023
H -4.738271 -0.950897 -3.063768
H 4.737860 0.950939 3.063593
H -4.017911 -2.310358 -3.950433
H 4.017815 2.310653 3.950129
H -4.988850 -2.608565 -2.488789
H 4.988917 2.608503 2.488522
C -1.693603 0.392610 3.334368
C 1.693603 -0.393721 -3.333998
C -4.026787 1.317303 3.268982
C 4.027005 -1.317867 -3.268665
H -2.495497 1.597426 1.786517
H 2.495963 -1.597997 -1.785945
H -1.366389 1.252402 3.939931
H 1.366516 -1.253731 -3.939317
H -1.974764 -0.419597 4.023748
H 1.974503 0.418383 -4.023607
H -0.841706 0.058497 2.728522
H 0.841701 -0.059656 -2.728138
H -3.727170 2.229418 3.808868
H 3.727540 -2.230200 -3.808266
H -4.907559 1.558143 2.657761
H 4.907908 -1.558330 -2.657486
H -4.341046 0.581329 4.025661
H 4.340995 -0.582022 -4.025580
H -2.570017 -2.861078 -2.070521
H 2.570139 2.861619 2.070073
H -1.632681 -1.499970 -3.752851
H 1.632405 1.500882 3.752503
H -0.990526 -0.944999 -2.182474
H 0.990193 0.945922 2.182142
H -2.204965 0.012643 -3.024882
H 2.204368 -0.011925 3.024702
C -4.343910 1.462311 -2.035360
C 4.343691 -1.461885 2.035895
C -4.133557 2.640196 0.109893
C 4.134014 -2.639636 -0.109500
H -4.928817 2.319854 -2.399983
H 4.928687 -2.319337 2.400586
H -3.850863 1.000030 -2.897955
H 3.850397 -0.999746 2.898421
H -5.046845 0.736210 -1.600340
H 5.046580 -0.735623 1.601062
H -4.739613 3.441847 -0.338135
H 4.739844 -3.441466 0.338506
H -4.820475 1.936705 0.599966
H 4.821174 -1.936036 -0.599072
H -3.494656 3.094455 0.875311
H 3.495381 -3.093594 -0.875322
H -2.136684 2.596507 -2.664540
H 2.136693 -2.596702 2.664578
H -2.516243 3.902424 -1.526960
H 2.516489 -3.902363 1.526787

105
Mg(CAAC)2
Mg 0.000270 -0.000267 -0.000066
C 1.615296 1.213476 -0.304216
C -1.615229 -1.213393 0.304076
N 2.861238 0.664386 -0.173368
N -2.861190 -0.664377 0.173263
C 3.994425 1.567738 -0.512283
C -3.994311 -1.567823 0.512162
C 3.295250 2.932251 -0.365301
C -3.295063 -2.932291 0.365141
C 1.785930 2.687855 -0.616490
C -1.785759 -2.687802 0.616327
C 1.367294 2.976921 -2.068726
C -1.367107 -2.976874 2.068553
C 0.918082 3.547974 0.307927
C -0.917890 -3.547861 -0.308120
H 1.541940 4.036071 -2.326423
H -1.541642 -4.036051 2.326218
H 1.913852 2.352556 -2.788564
H -1.913736 -2.352596 2.788411
H 0.294709 2.763573 -2.196868
H -0.294545 -2.763420 2.196726
H 1.039675 4.620981 0.081838
H -1.039447 -4.620875 -0.082042
H -0.147147 3.298734 0.182795
H 0.147342 -3.298612 -0.183010
H 1.180367 3.385870 1.363096
H -1.180204 -3.385744 -1.363277
C 2.929569 -0.729539 0.130001
C -2.929689 0.729579 -0.129950
C 2.806711 -1.674071 -0.928914
C -2.806814 1.674008 0.929048
C 2.839810 -3.042900 -0.633243
C -2.840059 3.042855 0.633533
C 2.927368 -3.490543 0.679108
C -2.927881 3.490644 -0.678749
C 2.955604 -1.175700 1.479623
C -2.956027 1.175895 -1.479507
C 2.968301 -2.565984 1.710460
C -2.968910 2.566189 -1.710190
C 2.654615 -1.279979 -2.387088
C -2.654400 1.279785 2.387156
H 2.779234 -3.766606 -1.448915
H -2.779383 3.766472 1.449279
H 2.940995 -4.560877 0.898690
H -2.941638 4.560999 -0.898219
C 3.023125 -0.353678 2.771107
C -3.023511 0.354006 -2.771078
H 2.999834 -2.919849 2.744389
H -3.000641 2.920164 -2.744076
C 4.409070 -0.472435 3.422597
C -4.409523 0.472541 -3.422455
C 2.543982 1.092392 2.775300
C -2.544022 -1.091948 -2.775465
H 5.181670 0.016789 2.813908
H -5.181986 -0.016925 -2.813786
H 4.407204 0.006243 4.414574
H -4.407618 -0.006002 -4.414497
H 4.709420 -1.522543 3.552733
H -4.710102 1.522604 -3.552433
C 1.349551 -1.809375 -2.985757
C -1.349229 1.809199 2.985573
C 3.843508 -1.757478 -3.228799
C -3.843136 1.757161 3.229152
H 2.599203 -0.186297 -2.426383
H -2.598904 0.186102 2.426342
H 1.229350 -1.454072 -4.021462
H -1.228733 1.453743 4.021191
H 1.319829 -2.910182 -3.002702
H -1.319596 2.910006 3.002688
H 0.483065 -1.455837 -2.406482
H -0.482870 1.455831 2.406009
H 3.798084 -1.330389 -4.243038
H -3.797509 1.329977 4.243342
H 4.808250 -1.477719 -2.782641
H -4.807953 1.477422 2.783144
H 3.839230 -2.853990 -3.332350
H -3.838873 2.853664 3.332804
H 2.326965 -0.889801 3.439807
H -2.327520 0.890350 -3.439776
H 2.408750 1.423351 3.816579
H -2.408844 -1.422778 -3.816793
H 1.586761 1.192508 2.246548
H -1.586709 -1.191877 -2.246846
H 3.250307 1.782127 2.300517
H -3.250110 -1.781903 -2.300650
C 5.180600 1.381561 0.426994
C -5.180522 -1.381718 -0.427078
C 4.513554 1.369914 -1.942382
C -4.513401 -1.370049 1.942280
H 6.011808 2.029435 0.111783
H -6.011664 -2.029670 -0.111857
H 4.932990 1.634611 1.463543
H -4.932918 -1.634727 -1.463638
H 5.536595 0.340237 0.398707
H -5.536601 -0.340424 -0.398758
H 5.277664 2.131936 -2.158844
H -5.277451 -2.132124 2.158776
H 4.982527 0.385145 -2.059680
H -4.982436 -0.385314 2.059601
H 3.720338 1.469862 -2.692585

H -3.720142 -1.469934 2.692446
H 3.433656 3.300398 0.662712
H -3.433469 -3.300423 -0.662877
H 3.725742 3.686365 -1.040775
H -3.725507 -3.686442 1.040605

105
Ca(CAAC)2
Ca -0.124714 -0.493323 -0.066068
C 2.230362 -1.124293 -0.364347
C -2.206983 1.081058 -0.004777
N 3.050475 -0.071602 -0.144521
N -3.154779 0.140514 -0.032419
C 4.524274 -0.299773 -0.262084
C -4.584869 0.572857 -0.079357
C 4.510735 -1.613660 -1.055875
C -4.408982 2.050129 0.314916
C 3.131156 -2.273885 -0.792930
C -2.933457 2.415408 0.000067
C 3.192176 -3.316610 0.336303
C -2.758582 3.059871 -1.384140
C 2.602015 -2.951970 -2.059469
C -2.348952 3.350609 1.057736
H 3.856549 -4.153832 0.061556
H -3.303865 4.016724 -1.442023
H 3.563113 -2.885686 1.277237
H -3.122017 2.410455 -2.193209
H 2.186559 -3.712714 0.535402
H -1.692722 3.256806 -1.575214
H 3.283793 -3.754647 -2.388922
H -2.881164 4.316488 1.065547
H 1.610650 -3.386762 -1.877777
H -1.286362 3.536815 0.852749
H 2.499705 -2.226237 -2.879989
H -2.422039 2.908153 2.062043
C 2.457066 1.114354 0.383767
C -2.578292 -1.172699 -0.126286
C 2.130801 1.145398 1.767385
C -2.078481 -1.558827 -1.380758
C 1.535929 2.296168 2.297034
C -1.120144 -2.620155 -1.474480
C 1.268858 3.400137 1.490168
C -0.745166 -3.311884 -0.297040
C 2.070573 2.178762 -0.473759
C -2.045066 -1.734452 1.099208
C 1.512825 3.324281 0.129353
C -1.197505 -2.898996 0.936360
C 2.363104 -0.034070 2.697113
C -2.410277 -0.820309 -2.661373
H 1.294667 2.334119 3.361449
H -0.811422 -2.992967 -2.450972
H 0.829687 4.302500 1.921023
H -0.072101 -4.170050 -0.363163
C 2.125480 2.245157 -2.003305
C -2.458556 -1.475822 2.541689
H 1.231019 4.165094 -0.510311
H -0.876490 -3.437514 1.831750
C 3.136697 3.289263 -2.497076
C -3.619825 -2.380271 2.984557
C 2.246126 0.951888 -2.805596
C -2.643707 -0.030369 3.001484
H 4.169625 2.976317 -2.294037
H -4.556255 -2.126046 2.471951
H 3.035431 3.434026 -3.584082
H -3.795783 -2.302206 4.070529
H 2.985613 4.264621 -2.011694
H -3.395725 -3.432656 2.752096
C 1.043611 -0.675812 3.131464
C -1.172275 -0.185223 -3.298577
C 3.197669 0.347670 3.922712
C -3.144999 -1.722077 -3.655712
H 2.904662 -0.798284 2.131536
H -3.076365 0.004661 -2.401723
H 1.233768 -1.509565 3.824742
H -1.449554 0.381585 -4.201382
H 0.376886 0.038713 3.635731
H -0.415174 -0.929026 -3.584171
H 0.501274 -1.117635 2.280692
H -0.708185 0.538434 -2.607282
H 3.463941 -0.551471 4.500006
H -3.458201 -1.151972 -4.545239
H 4.129048 0.858346 3.639118
H -4.041764 -2.167607 -3.199276
H 2.644420 1.018438 4.598479
H -2.504896 -2.550179 -3.997548
H 1.128984 2.649550 -2.260428
H -1.588277 -1.835871 3.118602
H 1.975974 1.155845 -3.852952
H -2.749412 0.000944 4.098076
H 1.585617 0.158809 -2.425731
H -1.778399 0.581663 2.708365
H 3.260535 0.537573 -2.802136
H -3.528221 0.452922 2.574374
C 5.241726 0.840111 -0.975910
C -5.469295 -0.226612 0.869715
C 5.188003 -0.458962 1.111216
C -5.194469 0.433231 -1.480901
H 6.322430 0.636360 -1.005294
H -6.511953 0.111690 0.777884
H 4.898578 0.964665 -2.008642
H -5.170705 -0.114503 1.917097
H 5.094503 1.790676 -0.440905
H -5.438457 -1.295739 0.610769
H 6.265109 -0.639694 0.976836
H -6.245375 0.758137 -1.446314
H 5.073651 0.456531 1.708407
H -5.174691 -0.611945 -1.819527
H 4.779192 -1.301125 1.681743
H -4.684025 1.054254 -2.227313
H 4.604803 -1.388214 -2.129536
H -4.584903 2.162833 1.395875
H 5.356515 -2.264025 -0.786721
H -5.133218 2.699771 -0.199182

105
[Be(CAAC)2]+
Be 0.000148 -0.000160 -0.000141
C -1.177710 -1.156406 0.192436
C 1.177725 1.156362 -0.192647
N -2.477538 -0.903799 0.281394
N 2.477611 0.903888 -0.281374
C -3.346146 -2.109570 0.555491
C 3.346139 2.109755 -0.555316
C -2.273019 -3.145729 0.926564
C 2.272974 3.145737 -0.926768
C -0.939068 -2.645801 0.334345
C 0.938908 2.645696 -0.334875
C -0.650760 -3.215531 -1.066808
C 0.649970 3.215661 1.066047
C 0.248239 -2.956129 1.244988
C -0.248116 2.955701 -1.246005
H -0.551991 -4.310631 -1.012995
H 0.551158 4.310743 1.011984
H -1.444709 -2.978600 -1.788020
H 1.443590 2.978905 1.787671
H 0.292923 -2.809465 -1.460489
H -0.293868 2.809612 1.459365
H 0.399442 -4.043779 1.317418
H -0.399519 4.043317 -1.318542
H 1.179839 -2.520258 0.852932
H -1.179769 2.519626 -0.854301
H 0.087418 -2.568044 2.259946
H -0.086783 2.567615 -2.260881
C -3.001759 0.412075 0.009282
C 3.001835 -0.411983 -0.009164
C -3.300654 0.727777 -1.343501
C 3.300535 -0.727708 1.343675
C -3.847364 1.979062 -1.636484
C 3.847336 -1.978946 1.636708
C -4.059746 2.921913 -0.639082
C 4.059919 -2.921765 0.639330
C -3.162182 1.376841 1.035173
C 3.162403 -1.376776 -1.035030
C -3.695508 2.623047 0.664404
C 3.695773 -2.622940 -0.664186
C -2.982048 -0.186952 -2.513825
C 2.981539 0.186818 2.514038
H -4.089798 2.225316 -2.671863
H 4.089647 -2.225202 2.672115
H -4.481726 3.899386 -0.882146
H 4.481936 -3.899210 0.882447
C -2.816205 1.290872 2.524981
C 2.816624 -1.290930 -2.524900
H -3.818700 3.383221 1.439962
H 3.819055 -3.383122 -1.439721
C -4.071541 1.203764 3.404543
C 4.072112 -1.204085 -3.404274
C -1.739501 0.311954 2.971088
C 1.740010 -0.312055 -2.971356
H -4.580287 0.237013 3.308744
H 4.581070 -0.237464 -3.308355
H -3.798300 1.335561 4.462426
H 3.799010 -1.335790 -4.462203
H -4.798323 1.986736 3.145369
H 4.798667 -1.987237 -3.144999
C -1.826391 0.385694 -3.336729
C 1.826211 -0.386402 3.337006
C -4.193299 -0.449763 -3.411887
C 4.192717 0.450115 3.412066
H -2.636765 -1.145102 -2.112245
H 2.635782 1.144805 2.112499
H -1.546411 -0.306691 -4.145104
H 1.545920 0.305879 4.145366
H -2.096628 1.349132 -3.795629
H 2.096958 -1.349678 3.795945
H -0.942239 0.549931 -2.708370
H 0.942109 -0.551113 2.708713
H -3.950140 -1.213668 -4.165880
H 3.949305 1.213955 4.166039
H -5.067537 -0.797880 -2.844963
H 5.066824 0.798517 2.845112
H -4.494037 0.457967 -3.956546
H 4.493787 -0.457501 3.956729
H -2.389870 2.288220 2.730981
H 2.390271 -2.288284 -2.730836
H -1.482191 0.509422 4.021885
H 1.482897 -0.509699 -4.022167
H -0.822034 0.435273 2.379388
H 0.822429 -0.435245 -2.379807
H -2.037883 -0.740724 2.899546
H 2.038415 0.740619 -2.899957
C -4.317842 -1.834265 1.693590
C 4.318270 1.834577 -1.693062
C -4.163646 -2.523291 -0.667110
C 4.163242 2.523766 0.667469
H -4.919632 -2.735500 1.877625
H 4.920127 2.735838 -1.876742
H -3.798305 -1.584170 2.625061
H 3.799120 1.584598 -2.624778
H -5.007192 -1.016662 1.437856
H 5.007525 1.016955 -1.437141
H -4.786372 -3.386366 -0.392556
H 4.785864 3.386937 0.392983

H -4.837160 -1.715939 -0.982030
H 4.836845 1.716595 0.982653
H -3.542798 -2.821466 -1.519749
H 3.542127 2.821912 1.519914
H -2.181093 -3.199867 2.021705
H 2.181316 3.199710 -2.021941
H -2.540191 -4.151249 0.573499
H 2.539914 4.151346 -0.573778

105
[Mg(CAAC)2]+
Mg -0.029012 0.073987 -0.053644
C -1.637552 -1.209983 -0.336363
C 1.670456 1.280747 0.030320
N -2.833546 -0.630594 -0.166390
N 2.830223 0.629018 -0.038124
C -4.021739 -1.505180 -0.465911
C 4.066767 1.492213 0.008992
C -3.343209 -2.887577 -0.417803
C 3.438558 2.869610 -0.264599
C -1.837160 -2.660052 -0.704127
C 1.938073 2.763916 0.098806
C -1.462732 -2.859753 -2.183213
C 1.625771 3.245818 1.526687
C -0.946616 -3.567026 0.150155
C 1.058956 3.533257 -0.888424
H -1.652012 -3.900224 -2.491124
H 1.841673 4.321620 1.621583
H -2.029107 -2.196392 -2.850985
H 2.216901 2.712235 2.283938
H -0.392541 -2.649422 -2.334814
H 0.560976 3.089833 1.759914
H -1.091604 -4.623435 -0.125690
H 1.281219 4.610867 -0.843742
H 0.115689 -3.323555 -0.000450
H -0.006506 3.399209 -0.648982
H -1.171565 -3.461993 1.220797
H 1.224926 3.193042 -1.920738
C -2.838159 0.754955 0.220733
C 2.825310 -0.809446 -0.000821
C -2.778462 1.744859 -0.798814
C 2.741774 -1.433606 1.275928
C -2.678613 3.091165 -0.430228
C 2.741589 -2.829171 1.343803
C -2.580653 3.464419 0.904233
C 2.783047 -3.602738 0.189997
C -2.707171 1.115701 1.590229
C 2.792215 -1.580640 -1.192079
C -2.579090 2.485186 1.886812
C 2.781989 -2.979918 -1.048522
C -2.794627 1.416818 -2.280414
C 2.607957 -0.659222 2.577380
H -2.659184 3.856524 -1.208659
H 2.695896 -3.317116 2.319233
H -2.489411 4.517673 1.178358
H 2.782825 -4.692756 0.256023
C -2.737850 0.226830 2.838583
C 2.755455 -1.120031 -2.653344
H -2.477654 2.780349 2.934256
H 2.761435 -3.592673 -1.953283
C -4.077997 0.391422 3.572114
C 4.093693 -1.374593 -3.361885
C -2.351354 -1.244643 2.742021
C 2.198414 0.259867 -2.983059
H -4.913010 -0.004898 2.979335
H 4.887883 -0.713179 -2.994926
H -4.055682 -0.149547 4.530199
H 3.985685 -1.203075 -4.443472
H -4.297137 1.447337 3.784668
H 4.433547 -2.410096 -3.218128
C -1.505237 1.862149 -2.972494
C 1.206066 -0.815538 3.168940
C -4.014040 2.025927 -2.978888
C 3.670179 -1.056428 3.605450
H -2.844241 0.327888 -2.377956
H 2.732312 0.404056 2.352092
H -1.505045 1.546532 -4.026760
H 1.120440 -0.268760 4.120267
H -1.379800 2.955090 -2.950108
H 0.951554 -1.869557 3.356560
H -0.618597 1.417630 -2.493982
H 0.439251 -0.403757 2.492283
H -4.086968 1.663610 -4.015483
H 3.633889 -0.380521 4.473078
H -4.952134 1.777551 -2.462331
H 4.684714 -1.016091 3.184810
H -3.944019 3.123860 -3.017387
H 3.509048 -2.078041 3.981672
H -1.975641 0.685524 3.492400
H 2.043219 -1.827929 -3.112080
H -2.226254 -1.644927 3.758975
H 2.084327 0.349923 -4.073243
H -1.407875 -1.393337 2.203639
H 1.204937 0.413232 -2.537100
H -3.108170 -1.857622 2.242579
H 2.835200 1.086409 -2.648775
C -5.135256 -1.318949 0.555979
C 5.083446 1.076124 -1.043780
C -4.616279 -1.230026 -1.850160
C 4.755094 1.428060 1.373464
H -5.996834 -1.940196 0.273694
H 5.964328 1.729904 -0.973685
H -4.833686 -1.610848 1.567183
H 4.685454 1.163645 -2.060486
H -5.473048 -0.271809 0.578881
H 5.421006 0.042028 -0.880792
H -5.426610 -1.950875 -2.030502
H 5.651781 2.063308 1.343836
H -5.050393 -0.224609 -1.910685
H 5.081316 0.405647 1.606112
H -3.883619 -1.349468 -2.657451
H 4.120423 1.792843 2.189765
H -3.458714 -3.318881 0.587598
H 3.534546 3.107528 -1.334652
H -3.807165 -3.589760 -1.124292
H 3.953167 3.666488 0.290152

105
[Ca(CAAC)2]+
Ca 0.155059 -0.512075 0.116531
C -2.268055 -1.237716 0.030330
C 2.192843 0.928143 -0.512498
N -3.082045 -0.216723 -0.130749
N 3.151151 0.153040 -0.031010
C -4.559950 -0.523051 -0.288915
C 4.569665 0.653411 -0.097290
C -4.561332 -1.989718 0.167940
C 4.370196 1.800223 -1.104973
C -3.104619 -2.496533 0.035284
C 2.863078 2.167104 -1.067964
C -2.842178 -3.219962 -1.297772
C 2.551185 3.327155 -0.107315
C -2.715566 -3.419950 1.189882
C 2.327833 2.514763 -2.457608
H -3.454313 -4.133011 -1.358779
H 3.030138 4.254214 -0.459400
H -3.082576 -2.592713 -2.168063
H 2.906217 3.129195 0.913592
H -1.784062 -3.512528 -1.376015
H 1.465765 3.494324 -0.054585
H -3.365634 -4.308532 1.204148
H 2.842364 3.402029 -2.858780
H -1.676456 -3.758680 1.079737
H 1.252338 2.737187 -2.410555
H -2.809628 -2.912689 2.161235
H 2.479674 1.687448 -3.166210
C -2.510934 1.102564 -0.234597
C 2.624617 -1.036568 0.584332
C -2.053462 1.514264 -1.514703
C 2.065507 -0.879866 1.873961
C -1.473028 2.779390 -1.642335
C 1.213979 -1.901753 2.391281
C -1.303654 3.604184 -0.536115
C 0.971617 -3.057714 1.634194
C -2.291879 1.898448 0.918293
C 2.261978 -2.130421 -0.257735
C -1.683590 3.151155 0.718715
C 1.474638 -3.172076 0.350133
C -2.085203 0.600153 -2.731191
C 2.241335 0.370676 2.712714
H -1.138447 3.118407 -2.623632
H 0.825306 -1.823391 3.407118
H -0.850570 4.591168 -0.649437
H 0.366546 -3.863251 2.055705
C -2.597830 1.588712 2.387597
C 2.697549 -2.453863 -1.684236
H -1.500276 3.782270 1.592000
H 1.257311 -4.067772 -0.236035
C -3.781789 2.416220 2.909071
C 3.912377 -3.393171 -1.690293
C -2.679251 0.133124 2.839341
C 2.843454 -1.316435 -2.691989
H -4.735349 2.105983 2.465580
H 4.814150 -2.895011 -1.312981
H -3.865592 2.303695 4.000501
H 4.122185 -3.749362 -2.710704
H -3.649092 3.485658 2.691269
H 3.731891 -4.272888 -1.055149
C -0.731838 -0.093414 -2.929606
C 0.910807 1.098635 2.921605
C -2.524889 1.322590 -4.004788
C 2.923515 0.067607 4.048130
H -2.811168 -0.192464 -2.538634
H 2.882841 1.056372 2.156043
H -0.676604 -0.578989 -3.915003
H 1.059258 2.022601 3.500880
H 0.109993 0.609095 -2.850961
H 0.179187 0.482525 3.463878
H -0.598559 -0.914608 -2.201663
H 0.469856 1.417428 1.959064
H -2.690035 0.595833 -4.813950
H 3.128751 0.998457 4.598760
H -3.461398 1.878169 -3.849433
H 3.877636 -0.458347 3.897984
H -1.766394 2.035799 -4.361529
H 2.295322 -0.566986 4.691886
H -1.715310 1.996721 2.907500
H 1.858732 -3.061245 -2.069288
H -2.687805 0.103543 3.939097
H 2.984583 -1.740701 -3.697513
H -1.816823 -0.458885 2.496551
H 1.947718 -0.681973 -2.713282
H -3.582233 -0.383148 2.494543
H 3.696848 -0.661201 -2.492967
C -5.419787 0.384229 0.577460
C 5.539844 -0.424450 -0.561242
C -5.022627 -0.360705 -1.738700
C 5.064303 1.160274 1.261124
H -6.477982 0.127036 0.428135
H 6.558058 -0.010896 -0.583325
H -5.199249 0.268102 1.643679
H 5.311189 -0.795665 -1.565514
H -5.290850 1.440682 0.299162

H 5.541619 -1.274707 0.137166
H -6.101646 -0.565991 -1.784244
H 6.103373 1.502059 1.150081
H -4.866936 0.665670 -2.100286
H 5.056345 0.361033 2.015090
H -4.529801 -1.061323 -2.424141
H 4.481395 2.009958 1.637124
H -4.871059 -2.045320 1.222403
H 4.634576 1.450929 -2.114621
H -5.275525 -2.594137 -0.408570
H 5.022152 2.656156 -0.880113

1.8 Crystal structure determinations

In all cases, a crystal of the compound was embedded in inert perfluoropolyalkylether (viscosity 1800 cSt; ABCR GmbH) and mounted using a Hampton Research CryoLoop. The selected crystal was then flash cooled to 100 K in a nitrogen gas stream and kept at this temperature during the experiment. In case of compound (Am)MgI·(CAAC) (**1**) the measurement was done at 160 K instead, since crystals of this compound seem to undergo a destructive phase transition at lower temperature. The crystal structures were measured on an Agilent SuperNova diffractometer with Atlas S2 detector using a CuKα (for **1**-**3**) or MoKα (compound $[(Am)MgI]_2$) microfocus source. The measured data were processed with the CrysAlisPro (v40.84a) software package.[S23] Using Olex2,[S24] the structures were solved with the ShelXT[S25] structure solution program using Intrinsic Phasing and refined with the ShelXL[S26] refinement package using Least Squares Minimization. All non-hydrogen atoms were refined anisotropically. All hydrogen atoms were placed in ideal positions and refined as riding atoms with relative isotropic displacement parameters.

In case of compound $[(Am)MgI]_2$, similarity restraints (SADI) and a distance restraint (DFIX) were used to model the co-crystallized *n*-hexane.

Compound **1** showed disorder of the *t*-butyl group, two isopropyl groups and of the co-crystallized benzene and toluene molecules. The disorder was modeled with the help of similarity restraints (SIMU, SADI). The relative occupancies of the two alternative orientations of each group were refined to 0.525(18)/0.475(18) (*t*Bu), 0.575(15)/0.425(15) (*i*Pr), 0.53(2)/0.47(2) (*i*Pr) and 0.629(9)/0.371(9) (benzene), respectively. The disordered solvent molecules required additional FLAT, ISOR and DFIX restraints to achieve reasonable geometries. The aromatic ring of toluene was refined as regular hexagon (AFIX 66). Since toluene is disordered about an inversion center, its occupancy was constrained to 0.5.

The crystal of compound **3** was a non-merohedral twin. The fractional contributions of the two twin domains were refined to 0.5754(10) and 0.4246(10) later on. While the data suggested a monoclinic crystal system at first glance, and the structure could be solved in space group $P2_1/n$, the resulting solution was of poor quality. Careful reinvestigation showed that the triclinic space group P-1 with two angles close but not equal to 90° is the better choice. In this setting, the asymmetric unit contains two molecules of **3**, which showed disorder of four isopropyl groups. One of the two co-crystallized *n*-pentane molecules was also

disordered. The disorder was modeled with the help of similarity restraints (SIMU, SADI) and rigid bond restraints (RIGU).[S27] The relative occupancies of the two alternative orientations of each group were refined to 0.63(3)/0.37(3) (*i*Pr), 0.688(14)/0.312(14) (2 x *i*Pr), 0.720(19)/0.280(19) (*i*Pr) and 0.514(7)/0.486(7) (*n*-pentane), respectively.

Crystallographic and refinement data are summarized in Table S3.

The crystal structure data of the compounds have been deposited with the Cambridge Crystallographic Data Centre. CCDC 2126546 (Am)MgI(CAAC) (**1**), 2126547 (Am)Mg·(CAAC) (**2**), 2126548 $(Am)_2Mg$ (**3**) and 2126549 $[(Am)MgI]_2$ contain the supplementary crystallographic data for the compounds. This data can be obtained free of charge from The Cambridge Crystallographic Data Centre via www.ccdc.cam.ac.uk/data_request/cif.

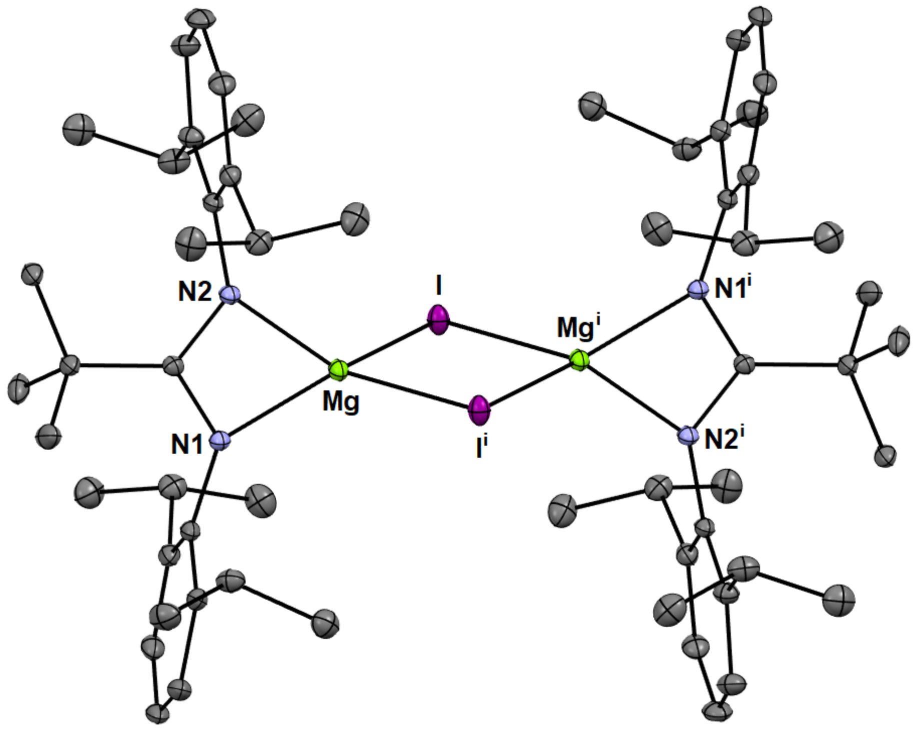

Figure S27. ORTEP plot for $[(Am)MgI]_2$. Ellipsoids represent 30% probability. Hydrogen atoms and co-crystallized *n*-hexane have been omitted for clarity. ^{i}-X,-Y,1-Z.

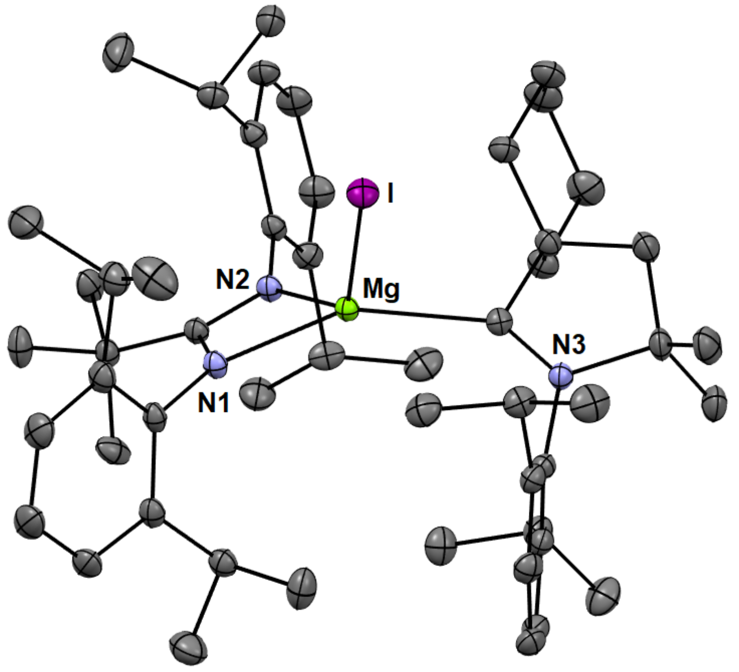

Figure S28. ORTEP plot for (Am)MgI·(CAAC) (**1**). Ellipsoids represent 30% probability. Disorder, hydrogen atoms and co-crystallized solvents (benzene/toluene) have been omitted for clarity.

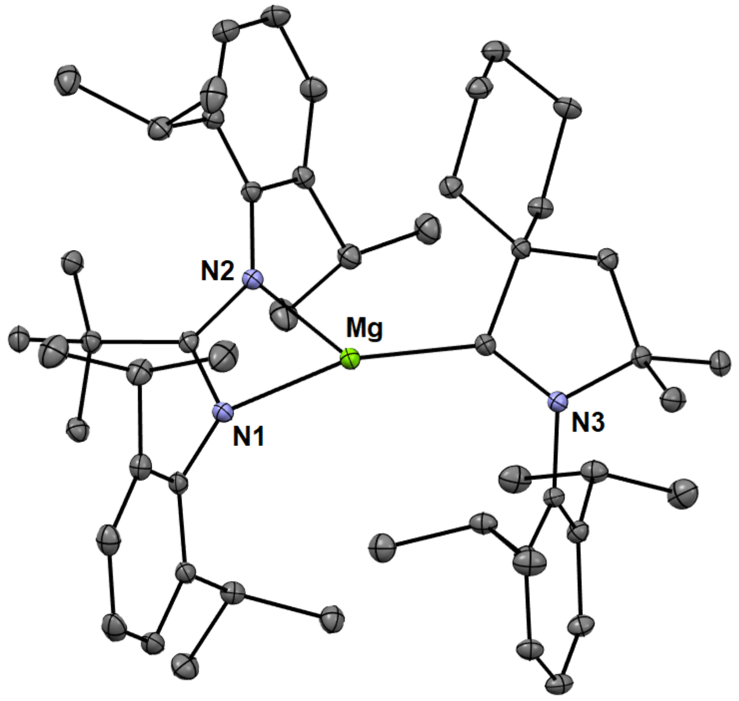

Figure S29. ORTEP plot for (Am)Mg·(CAAC) (**2**). Ellipsoids represent 30% probability. Hydrogen atoms have been omitted for clarity.

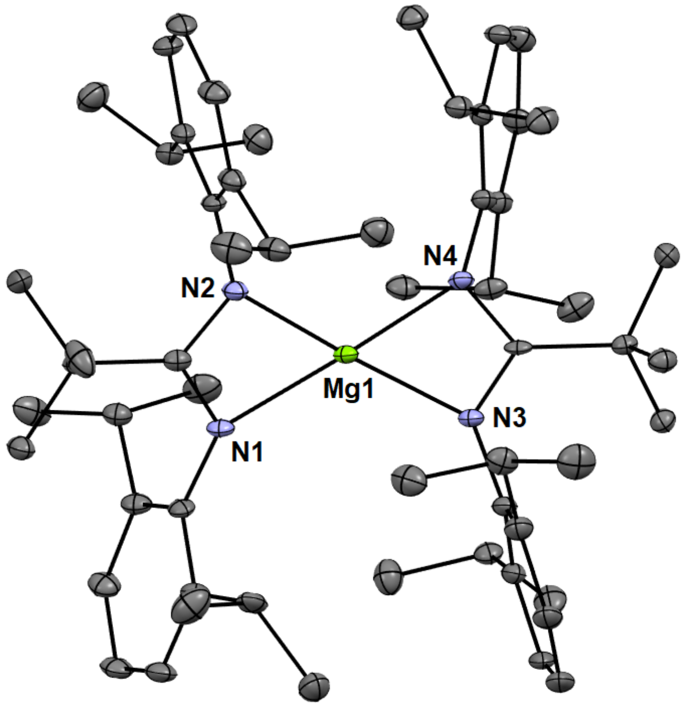

Figure S30. ORTEP plot for $(Am)_2Mg$ (**3**). Molecule 1 of two symmetry-independent molecules is shown. Ellipsoids represent 30% probability. Disorder and hydrogen atoms have been omitted for clarity.

Table S4. Crystal data and structure refinement.

Compound	$[(Am)MgI]_2 \cdot n$-Hexane	$[(Am)MgI \cdot (CAAC)] \cdot$Benzene $\cdot$0.5Toluene (**1**)	(Am)Mg·(CAAC) (**2**)	$[(Am)_2Mg] \cdot n$-Pentane (**3**)
Empirical formula	$C_{64}H_{100}I_2Mg_2N_4$	$C_{61.5}H_{88}IMgN_3$	$C_{52}H_{78}MgN_3$	$C_{63}H_{98}MgN_4$
Formula weight	1227.89	1020.55	769.48	935.76
Crystal color	colorless	colorless	bluish black	colorless
Temperature/K	100.0(6)	160.0(1)	100.0(9)	100.0(6)
Crystal system	triclinic	monoclinic	orthorhombic	triclinic
Space group	P-1	P21/n	Pbca	P-1
a/Å	9.9240(3)	10.51945(12)	17.6227(3)	12.2331(5)
b/Å	12.6128(4)	23.6504(3)	19.6722(4)	20.6715(7)
c/Å	15.1493(5)	22.9991(3)	26.7713(5)	23.0680(11)
α/°	104.763(3)	90	90	90.609(3)
β/°	107.472(3)	97.9283(12)	90	90.895(4)
γ/°	104.936(3)	90	90	98.901(3)
Volume/Å^3	1630.17(9)	5667.24(12)	9281.0(3)	5761.9(4)
Z	1	4	8	4
ρ_{calc}g/cm^3	1.251	1.196	1.101	1.079
μ/mm^{-1}	1.022	4.829	0.591	0.557
F(000)	642.0	2172.0	3384.0	2064.0
Crystal size/mm^3	0.711 × 0.397 × 0.351	0.626 × 0.165 × 0.095	0.168 × 0.136 × 0.082	0.235 × 0.154 × 0.097
Radiation	Mo Kα (λ = 0.71073)	Cu Kα (λ = 1.54184)	Cu Kα (λ = 1.54184)	Cu Kα (λ = 1.54184)
2Θ range for data collection/°	4.418 to 59.534	5.386 to 144.724	6.604 to 144.628	5.742 to 145.022
Index ranges	-12 ≤ h ≤ 13, -16 ≤ k ≤ 17, -21 ≤ l ≤ 20	-12 ≤ h ≤ 9, -28 ≤ k ≤ 18, -28 ≤ l ≤ 26	-21 ≤ h ≤ 20, -23 ≤ k ≤ 22, -31 ≤ l ≤ 32	-15 ≤ h ≤ 15, -25 ≤ k ≤ 25, -27 ≤ l ≤ 28
Reflections collected	28615	20712	27704	25077
Independent reflections	8212 [R_{int} = 0.0415, R_{sigma} = 0.0371]	10896 [R_{int} = 0.0311, R_{sigma} = 0.0430]	9019 [R_{int} = 0.0391, Rsigma = 0.0365]	25077 [R_{int} = not determined due to twinning, R_{sigma} = 0.0536]
Data/restraints/parameters	8212/4/337	10896/830/774	9019/0/522	25077/834/1433
Goodness-of-fit on F^2	1.066	1.026	1.049	1.011
Final R indexes [I>=2σ (I)]	R1 = 0.0334, wR2 = 0.0860	R1 = 0.0398, wR2 = 0.1023	R1 = 0.0485, wR2 = 0.1193	R_1 = 0.0605, wR_2 = 0.1630
Final R indexes [all data]	R1 = 0.0370, wR2 = 0.0888	R1 = 0.0450, wR2 = 0.1076	R1 = 0.0586, wR2 = 0.1277	R_1 = 0.0808, wR_2 = 0.1717
Largest diff. peak/hole / e Å^{-3}	1.43/-0.52	0.62/-1.26	0.47/-0.28	0.40/-0.54

2. References

[S1] A. Xia, H. M. El-Kaderi, M. Jane Heeg, C. H. Winter, *J. Organomet. Chem.* **2003**, 682, 224–232.

[S2] R. Jazzar, R. D. Dewhurst, J.-B. Bourg, B. Donnadieu, Y. Canac, G. Bertrand, *Angew. Chem. Int. Ed.* **2007**, 46, 2899–2902.

[S3] J. Hicks, M. Juckel, A. Paparo, D. Dange, C. Jones. *Organometallics* **2018**, 37, 24, 4810–4813.

[S4] S. Stoll, A. Schweiger, *J. Magn. Reson.* **2006**, *178*, 42–55.

[S5] F. Neese, *Wiley Interdiscip. Rev. - Comput. Mol. Sci.* **2012**, *2*, 73–78.

[S6] F. Neese, F. Wennmohs, U. Becker, C. Riplinger, The ORCA Quantum Chemistry Program Package. *J. Chem. Phys.* **2020**, *152* (22), 224108.

[S7] C. Lee, W. Yang, R. G. Parr, *Phys. Rev. B* **1988**, *37* (2), 785–789.

[S8] A. D. Becke, *J. Chem. Phys.* **1993**, *98* (7), 5648–5652.

[S9] F. Weigend, R. Ahlrichs, *Phys. Chem. Chem. Phys.* **2005**, *7*, 3297–3305.

[S10] F. Weigend, *Phys. Chem. Chem. Phys.* **2006**, *8* (9), 1057–1065.

[S11] V. Barone, M. Cossi, *J. Phys. Chem. A* **1998**, *102*, 1995–2001.

[S12] A. A. Auer, V. A. Tran, B. Sharma, G. L. Stoychev, D. Marx, F. Neese, *Mol. Phys.* **2020**, *118*, e1797916.

[S13] R. J. Gómez-Piñeiro, D. A. Pantazis, M. Orio, *ChemPhysChem* **2020**, *21* (24), 2667–2679.

[S14] M. J. Frisch, G. W. Trucks, H. B. Schlegel, G. E. Scuseria, M. A. Robb, J. R. Cheeseman, G. Scalmani, V. Barone, G. A. Petersson, H. Nakatsuji, X. Li, M. Caricato, A. V. Marenich, J. Bloino, B. G. Janesko, R. Gomperts, B. Mennucci, H. P. Hratchian, J. V. Ortiz, A. F. Izmaylov, J. L. Sonnenberg, D. Williams-Young, F. Ding, F. Lipparini, F. Egidi, J. Goings, B. Peng, A. Petrone, T. Henderson, D. Ranasinghe, V. G. Zakrzewski, J. Gao, N. Rega, G. Zheng, W. Liang, M. Hada, M. Ehara, K. Toyota, R. Fukuda, J. Hasegawa, M. Ishida, T. Nakajima, Y. Honda, O. Kitao, H. Nakai, T. Vreven, K. Throssell, J. A. Montgomery, J. E. Peralta, F. Ogliaro,

M. J. Bearpark, J. J. Heyd, E. N. Brothers, K. N. Kudin, V. N. Staroverov, T. A. Keith, R. Kobayashi, J. Normand, K. Raghavachari, A. P. Rendell, J. C. Burant, S. S. Iyengar, J. Tomasi, M. Cossi, J. M. Millam, M. Klene, C. Adamo, R. Cammi, J. W. Ochterski, R. L. Martin, K. Morokuma, O. Farkas, J. B. Foresman, D. J. Fox, *Gaussian 16 Rev. A.03*, Wallingford CT, **2016**.

[S15] (a) A. D. Becke, *J. Chem. Phys.*, **1993,** *98,* 5648-5652. (b) J. P. Perdew, J. A. Chevary, S. H. Vosko, K. A. Jackson, M. R. Pederson, D. J. Singh, C. Fiolhais, *Phys. Rev. B* **1992,** *46*, 6671-6687. (c) F. Weigend, R. Ahlrichs, *Phys. Chem. Chem. Phys.* **2005**, *7,* 3297-305. (d) F. Weigend, *Phys. Chem. Chem. Phys.*, **2006**, *8* 1057-1065.

[S16] S. Grimme, S. Ehrlich, L. Goerigk, *J. Comp. Chem.* **2011**, *32*, 1456 – 1465.

[S17] NBO 7.0. E. D. Glendening, J. K. Badenhoop, A. E. Reed, J. E. Carpenter, J. A. Bohmann, C. M. Morales, P. Karafiloglou, C. R. Landis, and F. Weinhold, Theoretical Chemistry Institute, University of Wisconsin, Madison, WI (2018).

[S18] N. van Eikema Hommes, Molecule, Erlangen, 2018.

[S19] T. A. Keith, AIMAll (Version 17.11.14), TK Gristmill Software, Overland Park KS USA, **2017.** R. F. W. Bader, *Chem. Rev*. **1991**, 91, 893–928.

[S20] G. Wang, J. E. Walley, D. A. Dickie, S. Pan, G. Frenking, R. J. Gilliard, *J. Am. Chem. Soc.* **2020**, *142*, 4560-4564.

[S21] C. Czernetzki, M. Arrowsmith, F. Fantuzzi, A. Gärtner, T. Tröster, I. Krummenacher, F. Schorr, H. Braunschweig, *Angew. Chem. Int. Ed.* **2021**, *60*, 20776–20780.

[S22] K. B. Wiberg, P. R. Rablen, *J. Org. Chem.* **2018**, *83*, 15463-15469.

[S23] Rigaku Oxford Diffraction, **2020**, CrysAlisPro Software system, version 1.171.40.84a, Rigaku Corporation, Oxford, UK.

[S24] O. V. Dolomanov, L. J. Bourhis, R.J. Gildea, J. A. K. Howard and H. Puschmann, *J. Appl. Cryst.*, 2009, **42**, 339–341.

[S25] G. M. Sheldrick, *Acta Cryst. A,* 2015, **71**, 3–8.

[S26] G. M. Sheldrick, *Acta Cryst. C,* 2015, **71**, 3–8.

[S27] A. Thorn, B. Dittrich and G. M. Sheldrick, *Acta Cryst. A,* 2012, **68**, 448–451.
